# Palladium-Catalyzed C2 Functionalization of an Iodinated Tryptophan Scaffold for Fluorescent Probe Discovery

**DOI:** 10.1021/acs.joc.6c00381

**Published:** 2026-04-23

**Authors:** Eoghan J. McArthur, Leung Yiu Wong, Valeria K. Burianova, Andrew Sutherland

**Affiliations:** School of Chemistry, 3526 University of Glasgow, The Joseph Black Building, Glasgow G12 8QQ, U.K.

## Abstract

Fluorescent unnatural amino acids are important tools for peptide and biomolecular imaging, yet the development of new probes that combine strong emission with minimal structural perturbation remains a challenge. Herein, we report a versatile strategy for the diversification of tryptophan based on a stable and isolable C2-iodinated building block that is readily compatible with palladium-catalyzed cross-coupling reactions. This approach allows for the installation of alkynyl and aryl substituents at the C2 position, providing rapid access to a range of unnatural tryptophan derivatives and peptides in good to high yields. Photophysical studies revealed that aryl-substituted tryptophan analogues exhibit strong fluorescence, high quantum yields, and environment-sensitive emission. Strong fluorescence was retained upon incorporation into a tryptophan-containing dipeptide, highlighting the potential of these relatively small C2-substituted analogues as imaging probes for biomolecular imaging applications.

## Graphical abstract

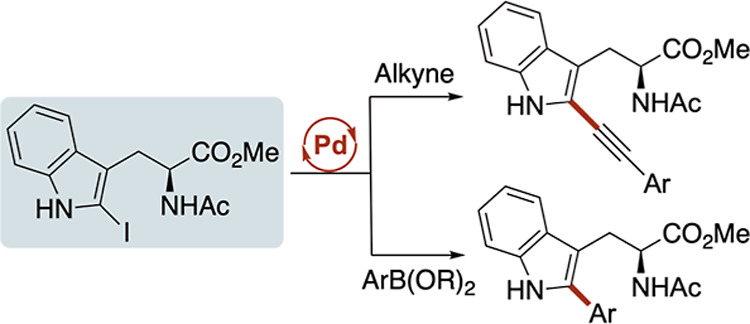

## Introduction

Fluorescence spectroscopy has become an important tool for investigating biological processes with high sensitivity and spatiotemporal resolution, enabling detailed examination of the protein structure, dynamics, and intermolecular interactions.^1^ The ability to report on local environments in real time has made fluorescence-based methods particularly valuable for studying complex and heterogeneous biological systems.^2,3^ In parallel, the incorporation of unnatural amino acids bearing extended π-conjugated structures into peptides and proteins has gained significant attention as a strategy to expand the photophysical capabilities of biomolecules.^4^ These residues can function as minimally perturbing, genetically, or chemically encodable fluorophores with emission properties that are tuned to their local surroundings. Compared with externally attached dyes, unnatural amino acids offer advantages in positional specificity and structural integration, while extended conjugation enables red-shifted absorption and emission, enhanced brightness, and tunable environmental properties.^5^ Consequently, unnatural amino acids have become valuable tools for fluorescence spectroscopy, enabling the molecular-level resolution of biological processes.

As the most fluorescent of the natural amino acids, tryptophan (**1**) has commonly been used for fluorescent probe development (Figure 1a).^6^ In particular, substitution at the C2 position of the indole ring has emerged as a useful strategy to access π-conjugated analogues such as **2–4**, with increased fluorescence intensity, red-shifted excitation and emission wavelengths, and improved photostability.^7–9^ The most widely employed strategy for C2 arylation of tryptophan- and tryptophan-based peptides relies on palladium-catalyzed C–H activation (Figure 1b). Early contributions by Albericio, Lavilla, and co-workers utilized aryl iodides as coupling partners in the presence of stoichiometric AgBF_4_ under microwave heating (90–150 °C).^10^ To eliminate the need for silver salts, Fairlamb and co-workers demonstrated that aryl boronic acids (5 equiv) could serve as effective coupling partners for tryptophan C2 arylation.^7^ While electron-rich boronic acids were prone to homocoupling, other aryl boronic acids coupled efficiently using Cu­(OAc)_2_ as the oxidant under mild conditions (40 °C). Seeking more general methods that require fewer equivalents of the coupling partner, subsequent studies have focused on more reactive, charged electrophiles. Fairlamb^11^ and Ackermann^12^ independently showed that diaryliodonium salts enabled efficient palladium-catalyzed C2 arylation at low catalyst loadings under mild conditions. Both groups further explored alternative charged coupling partners. Fairlamb and co-workers reported tosic acid-promoted, room-temperature C2 arylation using aryldiazonium salts,^13^ whereas Ackermann and co-workers employed thianthrenium salts to access sterically congested tryptophan peptide**–**drug conjugates.^14^ More recently, De Vos and co-workers revisited aryl boronic acids with the goal of mitigating palladium catalyst deactivation while employing water as the solvent and air as the oxidant.^15^ The use of a 4,5-diazafluoren-9-one ligand was found to stabilize the palladium catalytic system, enabling efficient C2 arylation of tryptophan- and tryptophan-based peptides under mild conditions.

**Figure 1:**
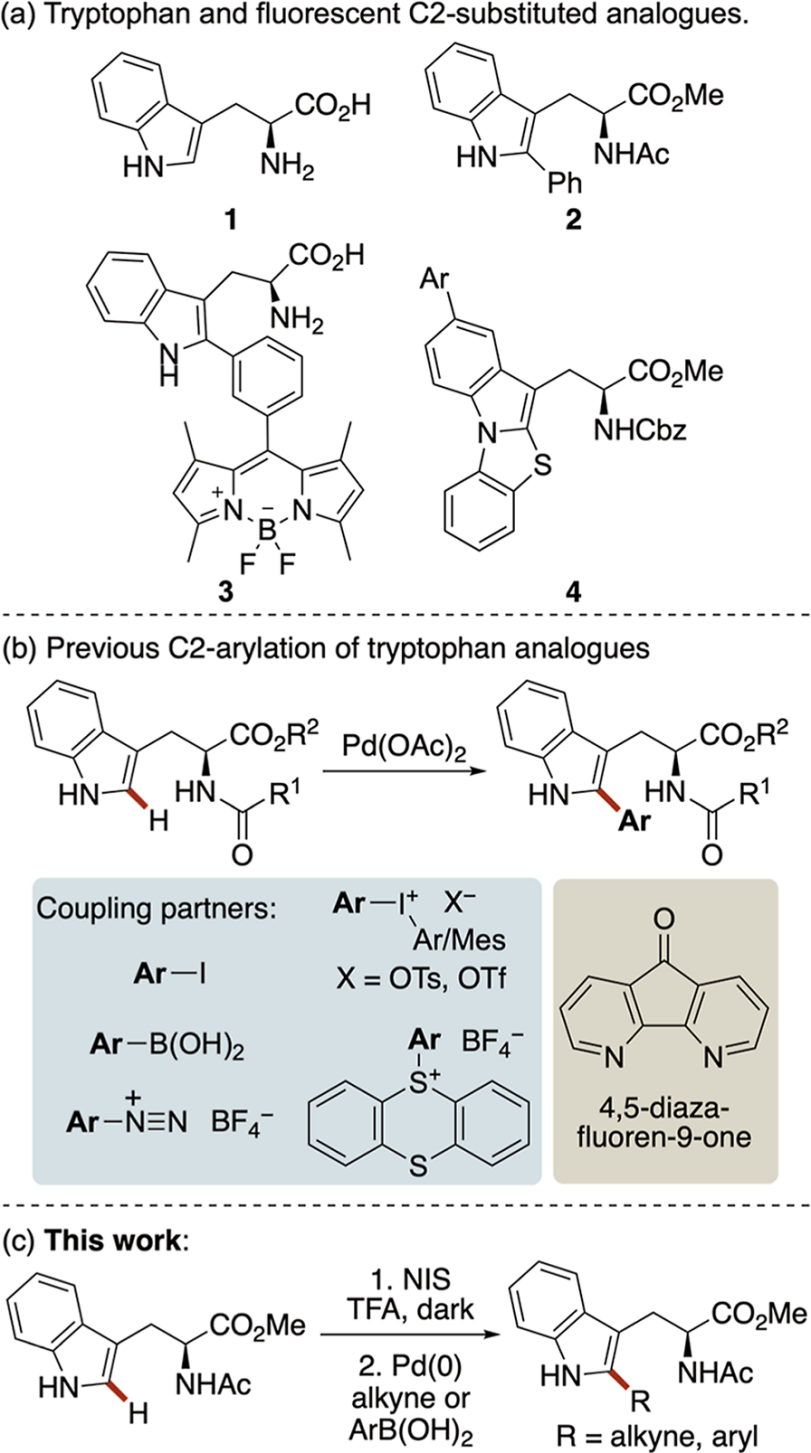
l-Tryptophan, fluorescent analogues, and methods for C2-substitution.

As part of a program aimed at discovering novel fluorescent amino acids, we sought to develop a general strategy for C2 functionalization of tryptophan that would enable the incorporation of not only aryl-conjugated groups but also rigid alkynes. Although the steps required two steps, we proposed that a broad range of C2-conjugated tryptophan analogues could be accessed via a halogenated intermediate. Here, we report the efficient synthesis of a C2-iodotryptophan and demonstrate its utility for diversification of the indole scaffold (Figure 1c). In addition, the photophysical properties of the resulting compounds are described and show that many C2-aryl analogues exhibit strong fluorescence and environmental sensitivity, including pH-dependent and solvatochromic emission.

## Results and Discussion

Reactions of tryptophan derivatives with iodinating reagents are often problematic due to competing oxidative degradation and cyclization pathways.^16^ These challenges have been addressed through acyl protection of the indole nitrogen, as demonstrated by Snider and Evano, who employed mercury­(II) trifluoroacetate and iodine to access 2-iodotryptophans in high yield.^17^ To avoid both indole protection and the use of mercury salts, we initially examined a reported C2-bromination using NBS and dibenzoyl peroxide.^18^ However, treatment of *N*-acetyl-l-tryptophan methyl ester (**5**) with these reagents afforded a mixture of products, with the C5-brominated derivative isolated as the major component in 33% yield (Table 1, entry 1). Given our prior work on iron­(III)-catalyzed electrophile activation for the functionalization of *N*-heterocycles, including tryptophan derivatives,^19^ we next evaluated Lewis acid activation of *N*-iodosaccharin. Whereas FeCl_3_ showed no reaction (entry 2), the super Lewis acid Fe­(NTf_2_)_3_ led to the formation of non-isolable products (entry 3). Sanford and co-workers prepared 2-iodotryptophan **6** as an intermediate of a two-step iodination and radiocyanation process.^20^ The iodination reaction was conducted by using NIS under acidic conditions and in the absence of light. Although **6** was not purified and isolated, this showed that direct preparation was possible. Consistent with this precedent, reaction of **5** with NIS in a 10:1 mixture of CH_2_Cl_2_/TFA initially afforded low isolable yields of **6** due to incomplete conversion and challenging separation from the starting material (entry 4). An increase in NIS loading (1.8 equiv) led to improved conversion and a higher isolated yield of 55% (entry 5). The amount of NIS could be reduced by batch addition (1.0 and then 0.4 equiv), which also resulted in a cleaner reaction and an optimized yield of 72% (entry 6). Gram-scale reactions using this approach produced **6** in similar yields. It should be noted that 2-iodotryptophan **6** was found to be a stable, isolable solid that could be purified by standard silica gel chromatography and amenable to long-term storage (>6 months at 6 °C).

**Table 1:**
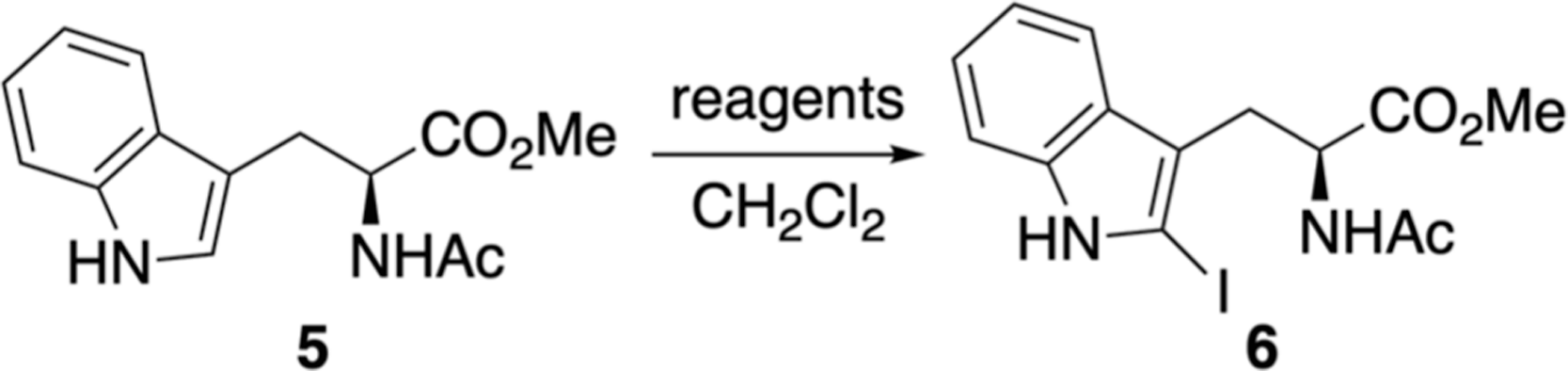
C2-Iodination of Tryptophan Derivative **5**

entry	reagents	temp (°C)	time (h)	yield (%)
1^a^	NBS, (PhCO_2_)_2_	40	1	33^b^
2^c^	NISac, FeCl_3_ (5 mol %)	rt	4	0
3	NISac, Fe(NTf_2_)_3_ (5 mol %)	40	1.5	0
4^d^	NIS (1 equiv), TFA	rt	2	25
5^d^	NIS (1.8 equiv), TFA	rt	1.5	55
6^d^	NIS (1.4 equiv), TFA	rt	1	72

a Dichloroethane was used as the solvent.

b Product was C5-bromide.

c Chloroform was used as the solvent.

d Reaction was done in the dark.

Having developed an efficient and scalable synthesis of 2-iodotryptophan derivative **6**, the application of this as a building block for palladium-catalyzed cross-coupling reactions was investigated (Scheme 1a). The Sonogashira reaction with a range of arylacetylenes of varying electronics under standard conditions gave coupled products **7a–7d** in 62**–**74% yields. Only minimal optimization was required; complete conversion was achieved by batch addition (×2) of both the palladium and copper catalysts. Evaluation of the photophysical properties of arylalkynes **7a–7d** revealed low quantum yields (vide infra), prompting a shift in focus toward the synthesis of C2-aryl analogues from iodotryptophan **6**. These targets were accessed via the Suzuki–Miyaura reaction with various aryl boronic acids and esters using the Buchwald precatalyst XPhos Pd G2 (2**–**3 mol %).^21,22^ Rapid reactions (1.2 to 3 h) were observed for both electron-rich and electron-deficient aryl boronic acids, and similarly, fast coupling was achieved with more sterically demanding boronates bearing biphenyl, dibenzothiophene, or carbazole substituents. Overall, the reactions proceeded cleanly, generating a small library of C2-aryl tryptophans **8a–8k** in 55**–**83% yields.

**Scheme 1:**
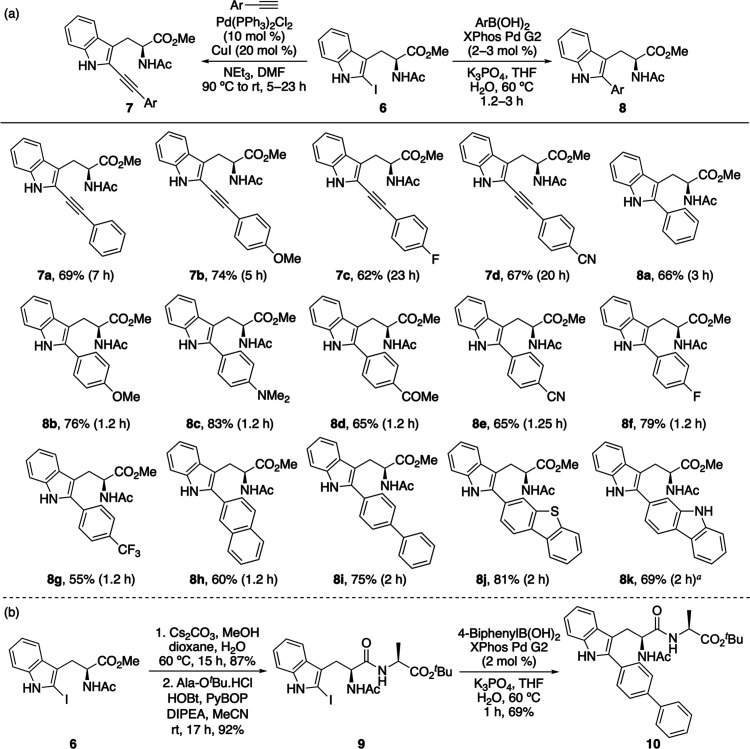
(a) Palladium-Catalyzed Synthesis of C2-Alkynyl and C2-Aryl Tryptophan Derivatives. (b) Late-Stage Suzuki–Miyaura Coupling Reaction Using Iodotryptophan-Containing Dipeptide **9**

a Prepared from the BPin ester.

As part of the evaluation of iodotryptophan **6** as a synthetic intermediate, we sought to demonstrate its utility as a peptide building block capable of undergoing late-stage cross-coupling reactions. As a proof-of-concept experiment, dipeptide **9** was prepared as a model substrate for such reactions (Scheme 1b). Ester hydrolysis of **6** using cesium carbonate gave the corresponding carboxylic acid in 87% yield. Subsequent amide bond formation with *tert*-butyl alaninate, employing HOBt and PyBOP as coupling agents, produced dipeptide **9** in 92% yield. With this substrate in hand, a Suzuki**–**Miyaura cross-coupling reaction with 4-biphenylboronic acid, using XPhos Pd G2 (2 mol %) under standard conditions, gave coupled product **10** in 69% yield. These results demonstrate that iodotryptophan **6** is a viable building block for peptide synthesis and is compatible with late-stage Suzuki**–**Miyaura cross-coupling reactions.

The photophysical properties of the C2-substituted tryptophans were then measured (Table 2).^23^ Although the extended conjugation of the arylalkynes led to red-shifted absorption and emission relative to l-tryptophan (**1**),^24^ the markedly reduced quantum yields rendered these less bright than the parent amino acid.^25^ In contrast, the majority of C2-aryl tryptophans displayed high quantum yields. The pronounced difference between the alkynyl and aryl series is exemplified by *p*-MeO-phenyl structural analogues **7b** and **8b**, which differ by 2 orders of magnitude in quantum yield. The C2-aryl tryptophans exhibit excellent brightness for small-molecule fluorophores, with values up to 32-fold stronger than those of l-tryptophan **1**. Although electron-deficient analogues such as **8e** and **8g** showed interesting properties, including red-shifted emission and large Stokes shifts, subsequent studies focused on further analysis of the brightest amino acids, *p*-dimethylaminophenyl **8c**, biphenyl **8i**, and carbazole **8k**.

**Table 2:** Photophysical Data of Selected α-Amino Acids

amino acid	λ_Abs_ (nm)^a^	ε (cm^–1^ M^–1^)	λ_Em_ (nm)^a^	Φ_F_ ^b^	brightness (cm^–1^ M^–1^)
**1**	279	5600	348	0.20	1120
**7b**	324	40,500	386	0.010	405
**8b**	304	10,900	362	1.0	10,900
**8c**	320	32,300	386	0.83	26,809
**8e**	330	18,000	441	0.73	13,180
**8g**	312	16,000	405	0.76	12,160
**8i**	318	35,300	417	1.0	35,300
**8k**	330	26,800	393	0.83	22,244

a Spectra were recorded at 3.5–10 μM in MeCN.

b Quantum yields (Φ_F_) were determined using l-tryptophan and anthracene as standards.

As the brightest amino acid, a detailed study of the photophysical properties of biphenyl **8i** was conducted (Figure 2).^23^ In a range of solvents with varying polarity, biphenyl **8i** demonstrated pronounced solvatochromism: while absorption spectra were similar across a range of solvents, the emission maxima underwent a bathochromic shift with increasing solvent polarity (Figure 2b). For example, the emission maximum shifted from 395 nm in toluene to 440 nm in water. Lipophilic amino acid fluorophores can exhibit markedly different emission intensities in hydrophobic versus hydrophilic environments and are therefore useful for probing lipid-rich regions such as cell membranes.^4,5^ Although sensitive to changes in solvent polarity, biphenyl **8i** displayed a comparable emission intensity in organic solvents such as ethyl acetate and toluene relative to water (Figure S18). To further investigate the solvatochromic behavior of the C2-aryl tryptophan analogues, carbazole **8k**, bearing a more polar side chain, was also examined (Figure S19). Similar solvatochromic behavior was observed; however, the bathochromic shift in emission maximum from toluene (384 nm) to water (403 nm) was smaller than that observed for biphenyl **8i**. In water, the relative emission intensity of **8k** decreased to approximately 60% of that observed in toluene. These results indicate that although both compounds retain relatively strong emission in aqueous media, precluding their use as membrane probes, amino acids such as biphenyl **8i** may serve as general solvatochromic probes, exhibiting emission wavelength changes across different polarity environments.

**Figure 2:**
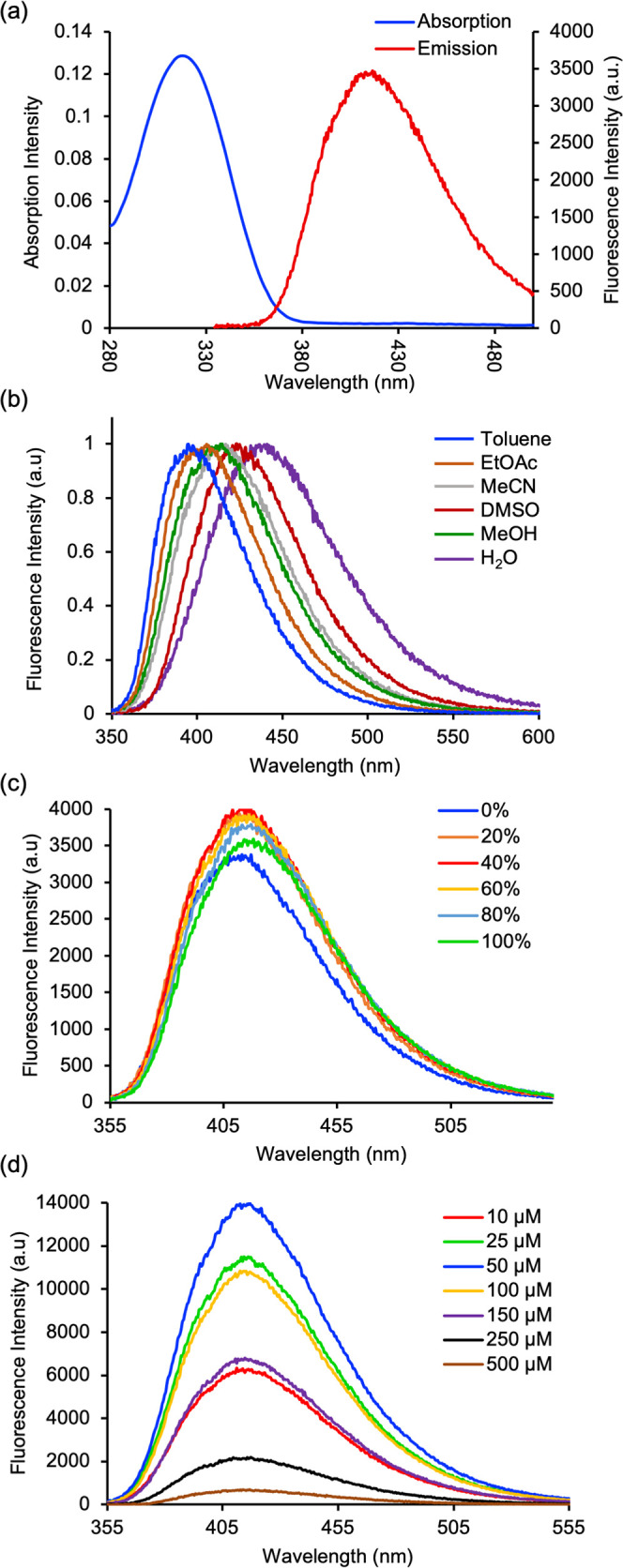
(a) Absorption and emission spectra of **8i**, recorded at 5 μM in MeCN. (b) Normalized fluorescence spectra of **8i** in various solvents (5 μM). (c) Emission spectra of **8i** in mixtures of 0**–**100% ethylene glycol in MeOH (5 μM). (d) Emission spectra of **8i** at various concentrations in MeCN.

The relative conformation of biaryl systems is known to modulate excited-state behavior through control of π-conjugation and nonradiative decay pathways.^2,3^ As such, biphenyl **8i** was investigated by using a viscosity-dependent fluorescence study. As shown in Figure 2c, the emission profile of **8i** exhibits negligible changes in both wavelength and relative intensity upon increasing solvent viscosity from methanol (η = 0.59 mPa s) to ethylene glycol (η = 13.5 mPa s). In systems in which excited-state planarization or intramolecular rotation governs emission, increased viscosity typically suppresses nonradiative relaxation and enhances fluorescence. The absence of such viscosity dependence for **8i** suggests that torsional relaxation about the biaryl bond is not a dominant deactivation pathway in the excited state and that the emissive state is not strongly coupled to conformational reorganization between twisted and planar geometries. Given the extended polyaromatic framework of biphenyl **8i**, concentration-dependent studies were performed to probe intermolecular interactions (Figure 2d). A progressive increase in fluorescence intensity was observed as the concentration was raised from 10 to 50 μM, consistent with minimal aggregation-induced quenching. At higher concentrations (250 μM), significant attenuation of emission was observed, likely due to π**–**π stacking interactions that facilitate nonradiative decay via excimer formation. It should be noted that this concentration is substantially higher than those typically employed in biological imaging, indicating that aggregation-associated quenching is unlikely to be a limiting factor for biphenyl **8i**. Collectively, these photophysical data indicate that biphenyl **8i** exhibits an emissive excited state that is insensitive to viscosity while remaining responsive to solvent polarity. The combination of conformationally decoupled emission, resistance to aggregation-induced quenching at working concentrations, and high brightness supports the potential of **8i** as a robust solvatochromic fluorophore suitable for imaging applications.

Following a detailed analysis of the photophysical properties of amino acid **8i**, the potential of this class of compounds as pH-responsive probes was evaluated. Based on its pendant dimethylaminophenyl substituent, amino acid **8c** was selected for further study. To enable investigation of its pH-dependent behavior, **8c** was converted to the corresponding free amino acid (Figure 3a). Ester hydrolysis using cesium carbonate, followed by deprotection of the amino group under acidic conditions, afforded amino acid **11** in an excellent yield. The pH-dependent photophysical properties of amino acid **11** were subsequently examined in methanol over a range of pH values. While the absorption spectra remained largely unchanged between pH 3 and 8 (Figure 3b), consistent with minimal perturbation of the ground-state electronic structure, the emission spectra exhibited a marked response (Figure 3c). Under basic to neutral conditions, the unprotonated dimethylamino group acts as an electron donor, facilitating strong charge-transfer emission. Upon protonation, the electron-donating ability of the dimethylamino group is significantly suppressed, with an 11.5-fold decrease in fluorescence intensity. The pronounced decrease in emission intensity upon protonation indicates that amino acid **11** functions as a “turn-off” fluorescent probe under acidic conditions, highlighting its potential application as a pH-responsive reporter.

**Figure 3:**
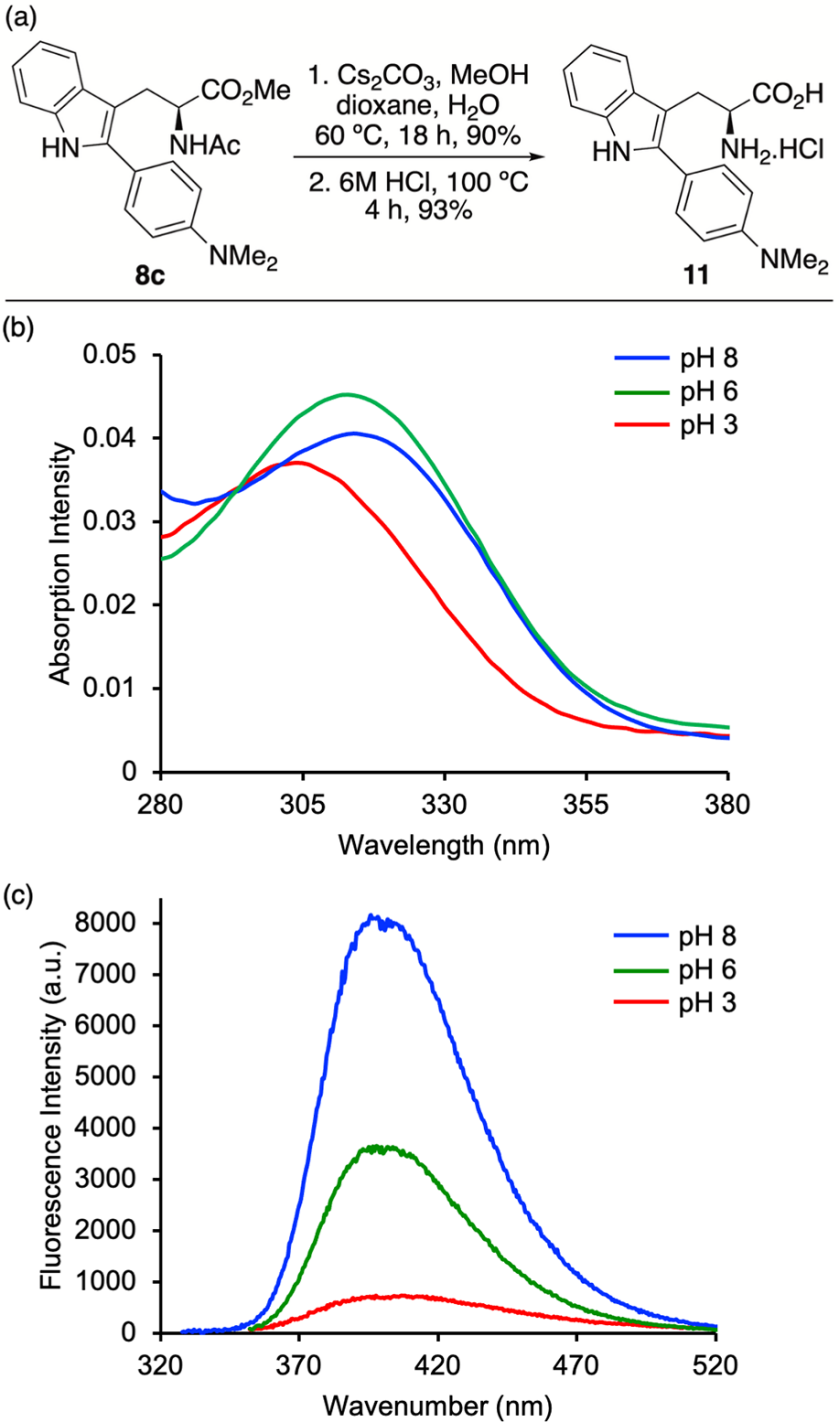
(a) Synthesis of deprotected amino acid **11**. (b) Absorption and (c) emission spectra of **11** at various pH in MeOH (5 μM).

To demonstrate the application of C2-aryl tryptophans for peptide synthesis and to assess their photophysical behavior in the presence of fluorescent proteinogenic amino acids, a model dipeptide was synthesized from biphenyl analogue **8i** and tryptophan (Figure 4a). Ester hydrolysis of **8i** using cesium carbonate gave carboxylic acid **12**, which was coupled with tryptophan methyl ester by using HOBt and PyBOP as coupling agents. This gave dipeptide **13** in 61% yield. Excitation of dipeptide **13** at wavelengths corresponding to tryptophan (280 nm) and biphenyl amino acid **8i** (320 nm) produced well-defined emission spectra (Figure 4b), which closely matched that of **8i**. These results demonstrate that C2-aryl tryptophans can function as relatively small fluorophores without interference or quenching from structurally related fluorescent amino acids. In a similar manner, excitation of dipeptide **10** produced an emission spectrum consistent with that of amino acid **8i** (Figure S16), indicating that structurally distinct amino acids (e.g., alanine) also do not interfere.

**Figure 4:**
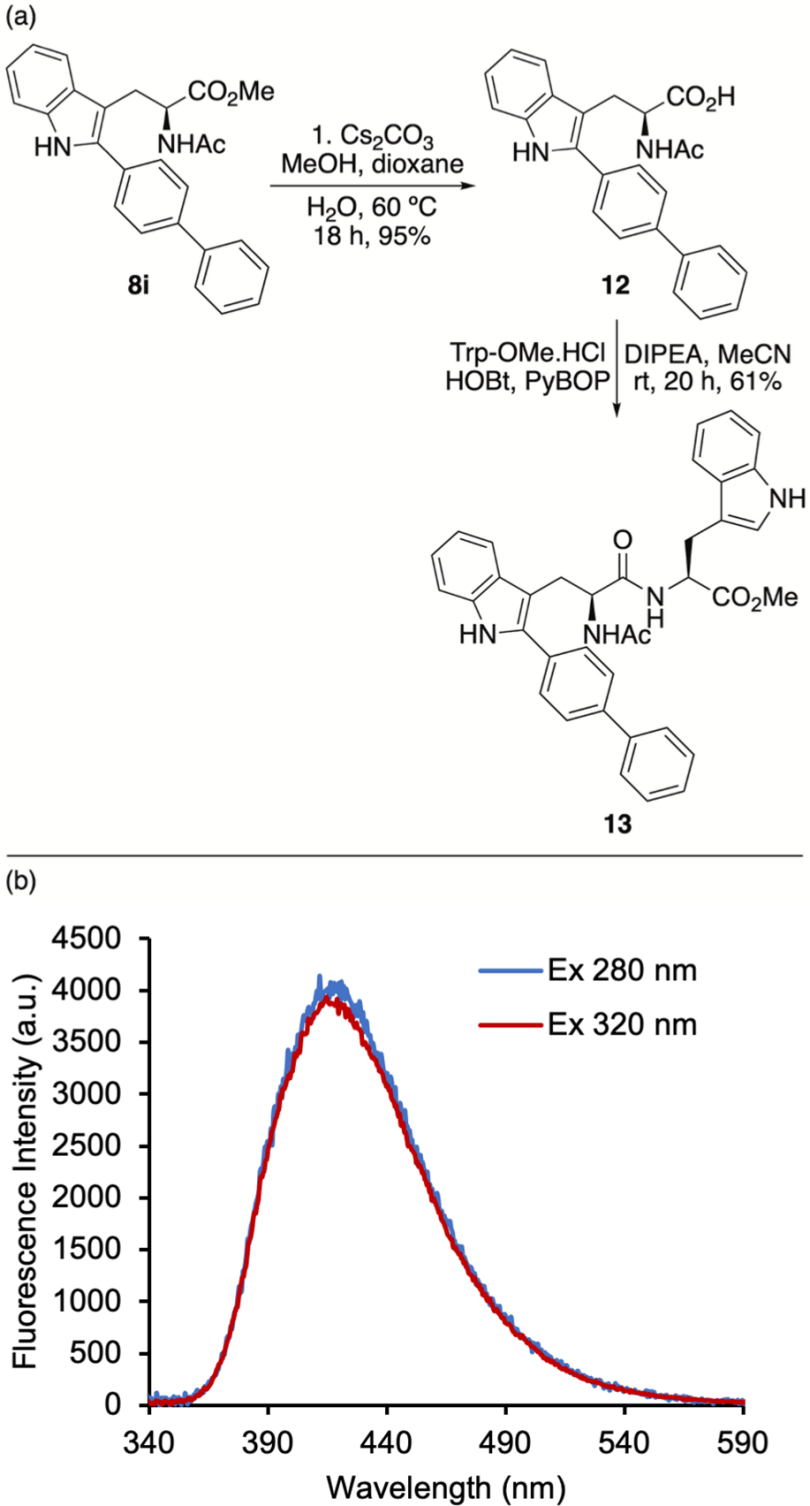
(a) Synthesis of dipeptide **13**. (b) Emission spectra of dipeptide **13** (5 μM in MeCN).

## Conclusions

In conclusion, we have developed a versatile strategy for the diversification of tryptophan based on a C2-iodinated building block that is stable, isolable, and readily amenable to palladium-catalyzed cross-coupling reactions. This approach enables the efficient incorporation of alkynyl and aryl substituents at the C2 position, providing rapid access to a diverse set of noncanonical tryptophan derivatives in 55**–**83% yields. Incorporation of iodotryptophan **6** into a dipeptide followed by a successful Suzuki–Miyaura reaction further demonstrates the synthetic utility of this building block, its compatibility with peptide synthesis, and subsequent late-stage cross-coupling reactions. Photophysical evaluation identified aryl-substituted analogues as particularly bright fluorophores with pronounced environment sensitivity. Importantly, the preservation of strong emission upon peptide incorporation highlights the potential of these modified tryptophans with relatively small C2-substituents as minimally perturbing fluorescent probes for peptide and biomolecular imaging applications.

## Experimental Section

All reagents and starting materials were obtained from commercial sources and used as received. Reactions were performed in air unless otherwise mentioned. All reactions performed at elevated temperatures were heated by using an oil bath. Brine refers to a saturated aqueous solution of sodium chloride. Flash column chromatography was performed using silica gel 60 (40**–**63 μm). Aluminum-backed plates precoated with silica gel 60F_254_ were used for thin layer chromatography and were visualized with a UV lamp or by staining with potassium permanganate, vanillin, or ninhydrin. ^1^H NMR spectra were recorded on a NMR spectrometer at either 400 or 500 MHz, and data are reported as follows: chemical shift in ppm relative to the solvent as an internal standard (CHCl_3_, δ 7.26 ppm; CH_3_OH, δ 3.31 ppm; DMSO_,_ δ 2.50), multiplicity (s = singlet, d = doublet, t = triplet, q = quartet, m = multiplet or overlap of nonequivalent resonances, integration). ^13^C NMR spectra were recorded on an NMR spectrometer at either 101 or 126 MHz, and data are reported as follows: chemical shift in ppm relative to tetramethylsilane or the solvent as an internal standard (CDCl_3_, δ 77.2 ppm; CD_3_OD, δ 49.0 ppm; DMSO-*d*_6,_ δ 39.5), multiplicity with respect to hydrogen (deduced from DEPT experiments, C, CH, CH_2_ or CH_3_). Infrared spectra were recorded on an FTIR spectrometer; wavenumbers are indicated in cm^–1^. Mass spectra were recorded using electrospray techniques. HRMS spectra were recorded using quadrupole time-of-flight (Q-TOF) mass spectrometers. Melting points are uncorrected. Optical rotations were determined as solutions irradiating with the sodium D line (λ = 589 nm) using a polarimeter. [α]_D_ values are given in units of 10^–1^ deg cm^–1^ g^–1^. UV–vis and fluorescence spectra were recorded on a fluorescence and absorbance spectrometer. Absorbance spectra were recorded with an integration time of 0.05 s and a band-pass of 5 nm. Fluorescence spectra were recorded with an excitation and emission band-pass of 5 nm, an integration time of 2 s, and with detector accumulations set to 1. Quantum yields were determined using a comparative method against two standards.^1,26^ Anthracene (Φ = 0.27, in ethanol) and l-tryptophan (Φ = 0.14 in methanol) were used as the standard references.

### (2*S*)-*N*-Acetyltryptophan Methyl Ester (**5**).^7^

Methyl l-tryptophanate hydrochloride (0.255 g, 1.00 mmol) was suspended in anhydrous tetrahydrofuran (10 mL) under argon and cooled to 0 °C. Triethylamine (0.104 mL, 1.10 mmol) was added dropwise, followed by acetic anhydride (0.146 mL, 1.05 mmol). The mixture was heated to 80 °C and stirred for 2 h. The reaction mixture was quenched with water (30 mL) and extracted with ethyl acetate (3 × 30 mL). The combined organic layers were washed with 1 M hydrochloric acid solution (6 mL), saturated aqueous solutions of sodium bicarbonate solution (6 mL), and brine (6 mL). The organic layer was dried (MgSO_4_), filtered, and concentrated in vacuo to give (2*S*)-*N*-acetyltryptophan methyl ester (**5**) as a white solid (0.237 g, 91%). Mp 148–151 °C (lit.^7^ 156**–**157 °C); [α]_D_^17^ +83.4 (*c* 0.1, CHCl_3_); ^1^H NMR (400 MHz, CDCl_3_) δ 8.22 (br s, 1H), 7.53 (d, *J* = 7.8 Hz, 1H), 7.36 (d, *J* = 7.8 Hz, 1H), 7.20 (t, *J* = 7.8 Hz, 1H), 7.12 (t, *J* = 7.8 Hz, 1H), 6.97 (d, *J* = 2.5 Hz, 1H), 5.98 (d, *J* = 7.5 Hz, 1H), 4.96 (ddd, *J* = 7.5, 5.5, 5.3 Hz, 1H), 3.70 (s, 3H), 3.36 (dd, *J* = 14.7, 5.5 Hz, 1H), 3.30 (dd, *J* = 14.7, 5.3 Hz, 1H), 1.96 (s, 3H); ^13^C­{^1^H} NMR (101 MHz, CDCl_3_) δ 172.6, 169.8, 136.3, 127.9, 122.8, 122.4, 119.9, 118.7, 111.4, 110.3, 53.2, 52.5, 27.7, 23.4; MS (ESI) *m/z:* 261 (M + H^+^, 100).

### (2*S*)-*N*-Acetyl-5′-bromotryptophan Methyl Ester.^27^

(2*S*)-*N*-Acetyltryptophan methyl ester (**5**) (0.100 g, 0.314 mmol) was dissolved in anhydrous 1,2-dichloroethane (3 mL) under argon. *N*-Bromosuccinimide (0.0890 g, 0.408 mmol) and benzoyl peroxide (0.00400 g, 0.0160 mmol) were then added to the solution. The mixture was stirred at 40 °C for 2 h. The reaction mixture was concentrated in vacuo. Purification by flash chromatography eluting with 33% ethyl acetate in hexane gave (2*S*)-*N*-acetyl-5′-bromotryptophan methyl ester as a yellow oil (0.0407 g, 33%). Spectroscopic data were consistent with the literature.^27^ [α]_D_^17^ +76.5 (*c* 0.1, CHCl_3_); ^1^H NMR (400 MHz, CDCl_3_) δ 8.29 (br s, 1H), 7.64–7.62 (m, 1H), 7.26 (dd, *J* = 8.5, 1.5 Hz, 1H), 7.22 (d, *J* = 8.5 Hz, 1H), 6.98 (d, *J* = 2.0 Hz, 1H), 6.04 (d, *J* = 7.7 Hz, 1H), 4.94 (ddd, *J* = 7.7, 5.3, 5.0 Hz, 1H), 3.72 (s, 3H), 3.31 (dd, *J* = 14.8, 5.3 Hz, 1H), 3.25 (dd, *J* = 14.8, 5.0 Hz, 1H), 2.00 (s, 3H); ^13^C­{^1^H} NMR (101 MHz, CDCl_3_) δ 172.4, 169.9, 134.8, 129.6, 125.2, 124.1, 121.5, 113.1, 112.9, 110.1, 53.1, 52.6, 27.7, 23.4; MS (ESI) *m/z:* 339 (M + H^+^, 100).

### (2*S*)-*N*-Acetyl-2′-iodotryptophan Methyl Ester (**6**)

(2*S*)-*N*-Acetyltryptophan methyl ester (**5**) (0.975 g, 3.75 mmol) was dissolved in a mixture of dichloromethane (114 mL) and trifluoroacetic acid (11 mL). *N*-Iodosuccinimide (0.843 g, 3.75 mmol) was then added to the solution in the dark. The mixture was stirred at room temperature for 0.5 h, and another portion of *N*-iodosuccinimide was added (0.338 g, 1.50 mmol). The reaction mixture was stirred for 0.5 h. The reaction mixture was washed with 2 M sodium thiosulfate solution (2 × 40 mL), saturated sodium hydrogen carbonate solution (2 × 40 mL), and brine (40 mL). The organic layer was dried (MgSO_4_) and concentrated in vacuo. Purification by flash chromatography eluting with 10**–**20% ethyl acetate in dichloromethane gave (2*S*)-*N*-acetyl-2′-iodotryptophan methyl ester (**6**) as a white solid (1.05 g, 72%). Mp 52**–**55 °C; IR (neat) 3259, 2949, 2361, 1731, 1649, 1520, 1434, 1338, 1211, 741 cm^–1^; [α]_D_^20^ +37.5 (*c* 0.1, CHCl_3_); ^1^H NMR (400 MHz, CDCl_3_) δ 8.16 (br s, 1H), 7.51 (d, *J* = 7.8 Hz, 1H), 7.29 (d, *J* = 7.8 Hz, 1H), 7.16–7.06 (m, 2H), 6.01 (d, *J* = 8.1 Hz, 1H), 4.94 (ddd, *J* = 8.1, 6.0, 5.6 Hz, 1H), 3.70 (s, 3H), 3.26 (dd, *J* = 14.7, 6.0 Hz, 1H), 3.22 (dd, *J* = 14.7, 5.6 Hz, 1H), 1.97 (s, 3H); ^13^C­{^1^H} NMR (101 MHz, CDCl_3_) δ 172.4, 170.1, 139.0, 127.7, 122.5, 120.0, 117.7, 116.1, 110.8, 79.8, 52.8, 52.7, 29.6, 23.5; HRMS (ESI) *m/z*: [M + Na]^+^ calcd for C_14_H_15_IN_2_O_3_Na 409.0020; found 409.0026.

### Methyl (2*S*)-2-Acetylamino-3-(2′-phenylethynyl-1′*H*-indol-3′-yl)­propanoate (**7a**)

To an oven-dried microwave vial were added (2*S*)-*N*-acetyl-2′-iodotryptophan methyl ester (**6**) (100 mg, 0.259 mmol), phenylacetylene (28.4 μL, 0.259 mmol), bis­(triphenylphosphine)­palladium­(II) dichloride (9.09 mg, 0.0130 mmol), and copper­(I) iodide (4.93 mg, 0.0259 mmol). The microwave vial was sealed and placed under an argon atmosphere. To the vial was added a degassed mixture of triethylamine (10.5 mL) and *N*,*N*-dimethylformamide (4.5 mL). The reaction mixture was stirred at 90 °C for 0.5 h, then cooled to room temperature, and stirred for 3 h. Following the addition of the second batch of bis­(triphenylphosphine)­palladium­(II) dichloride (9.09 mg, 0.0130 mmol) and copper­(I) iodide (4.93 mg, 0.0259 mmol), the reaction mixture was stirred at 90 °C for 0.5 h, then allowed to cool to room temperature, and stirred for 3 h. The reaction mixture was concentrated in vacuo, and the resulting residue was dissolved in ethyl acetate (20 mL) and washed with water (2 × 20 mL) and brine (20 mL). The combined organic layers were dried (MgSO_4_) and concentrated in vacuo. Purification by flash chromatography eluting with 50–60% ethyl acetate in hexane gave methyl (2*S*)-2-acetylamino-3-(2′-phenylethynyl-1′*H*-indol-3′-yl)­propanoate (**7a**) as a pale-yellow solid (63.6 mg, 69%). Mp 58–61 °C; IR (neat) 3270, 2920, 1733, 1649, 1518, 1489, 1436, 1215, 743, 689 cm^–1^; [α]_D_^20^ +56.9 (*c* 0.1, CHCl_3_); ^1^H NMR (400 MHz, CDCl_3_) δ 8.68 (s, 1H), 7.57–7.53 (m, 2H), 7.51 (d, *J* = 8.0 Hz, 1H), 7.39–7.34 (m, 3H), 7.30 (d, *J* = 8.0 Hz, 1H), 7.23 (ddd, *J* = 8.0, 6.9, 0.8 Hz, 1H), 7.12 (ddd, *J* = 8.0, 6.9, 0.8 Hz, 1H), 6.22 (d, *J* = 8.0, 1H), 5.00 (ddd, *J* = 8.0, 5.5, 5.0 Hz, 1H), 3.63 (s, 3H), 3.51 (dd, *J* = 14.5, 5.5 Hz, 1H), 3.46 (dd, *J* = 14.5, 5.0 Hz, 1H), 1.92 (s, 3H); ^13^C­{^1^H} NMR (101 MHz, CDCl_3_) δ 172.3, 170.0, 136.1, 131.4, 128.8, 128.7, 127.4, 123.9, 122.5, 120.4, 119.0, 118.2, 116.1, 111.2, 95.2, 81.1, 53.0, 52.6, 27.8, 23.3; HRMS (ESI) *m*/*z*: [M + Na]^+^ calcd for C_22_H_20_N_2_O_3_Na 383.1366; found 383.1363.

### Methyl (2*S*)-2-Acetylamino-3-(2′-[4″-methoxyphenyl]­ethynyl-1′*H*-indol-3′-yl)­propanoate (**7b**)

To an oven-dried microwave vial were added (2*S*)-*N*-acetyl-2′-iodotryptophan methyl ester (**6**) (100 mg, 0.259 mmol), 4-ethynylmethoxybenzene (33.6 μL, 0.259 mmol), bis­(triphenylphosphine)­palladium­(II) dichloride (9.09 mg, 0.0259 mmol), and copper­(I) iodide (4.93 mg, 0.0518 mmol). The microwave vial was sealed and placed under an argon atmosphere. To the vial was added a degassed mixture of triethylamine (10.5 mL) and *N*,*N*-dimethylformamide (4.5 mL). The reaction mixture was stirred at 90 °C for 5 h. Following the addition of the second batch of *bis*(triphenylphosphine)­palladium­(II) dichloride (9.09 mg, 0.0130 mmol) and copper­(I) iodide (4.93 mg, 0.0259 mmol), the reaction mixture was stirred at 90 °C for 18 h. The reaction mixture was concentrated in vacuo, and the resulting residue was dissolved in ethyl acetate (20 mL) and washed with water (2 × 20 mL) and brine (20 mL). The combined organic layers were dried (MgSO_4_) and concentrated in vacuo. Purification by flash chromatography eluting with 50% ethyl acetate in hexane gave methyl (2*S*)-2-acetylamino-3-(2’-[4″-methoxyphenyl]­ethynyl-1′*H*-indol-3′-yl)­propanoate (**7b**) as a light brown solid (75.0 mg, 74%). Mp 61**–**67 °C; IR (neat) 3252, 2950, 1735, 1651, 1604, 1505, 1436, 1245, 1171, 1027, 831, 741 cm^–1^; [α]_D_^23^ +49.6 (*c* 0.1, CHCl_3_); ^1^H NMR (400 MHz, CDCl_3_) δ 8.53 (br s, 1H), 7.53–7.46 (m, 3H), 7.29 (d, *J* = 8.0 Hz, 1H), 7.21 (ddd, *J* = 8.0, 7.1, 1.1 Hz, 1H), 7.11 (ddd, *J* = 8.0, 7.1, 1.1 Hz, 1H), 6.88 (d, *J* = 8.9 Hz, 2H), 6.21 (d, *J* = 7.9 Hz, 1H), 4.98 (dt, *J* = 7.9, 5.5 Hz, 1H), 3.82 (s, 3H), 3.63 (s, 3H), 3.48 (dd, *J* = 14.5, 5.5 Hz, 1H), 3.44 (dd, *J* = 14.5, 5.5 Hz, 1H), 1.91 (s, 3H); ^13^C­{^1^H} NMR (101 MHz, CDCl_3_) δ 172.3, 169.9, 160.1, 136.0, 133.0, 127.5, 123.7, 120.4, 118.9, 118.7, 115.5, 114.5, 114.4, 111.1, 95.2, 79.7, 55.4, 53.0, 52.6, 27.7, 23.4; HRMS (ESI) *m*/*z*: [M + H]^+^ calcd for C_23_H_22_N_2_O_4_H 391.1652; found 391.1649.

### Methyl (2*S*)-2-Acetylamino-3-(2′-[4″-fluorophenyl]­ethynyl-1′*H*-indol-3′-yl)­propanoate (**7c**)

To an oven-dried microwave vial were added (2*S*)-*N*-acetyl-2′-iodotryptophan methyl ester (**6**) (100 mg, 0.259 mmol), 1-ethynyl-4-fluorobenzene (29.7 μL, 0.259 mmol), *bis*(triphenylphosphine)­palladium­(II) dichloride (9.09 mg, 0.0130 mmol), and copper­(I) iodide (4.93 mg, 0.0259 mmol). The microwave vial was sealed and placed under an argon atmosphere. To the vial was added a degassed mixture of triethylamine (10.5 mL) and *N*,*N*-dimethylformamide (4.5 mL). The reaction mixture was stirred at 90 °C for 0.5 h, then cooled to 70 °C, and stirred for 3 h. Following the addition of the second batch of *bis*(triphenylphosphine)­palladium­(II) dichloride (9.09 mg, 0.0130 mmol) and copper­(I) iodide (4.93 mg, 0.0259 mmol), the reaction mixture was stirred at 90 °C for 0.5 h, then allowed to cool to 70 °C, and stirred for 19 h. The reaction mixture was concentrated in vacuo, and the resulting residue was dissolved in ethyl acetate (20 mL) and washed with water (2 × 20 mL) and brine (20 mL). The combined organic layers were dried (MgSO_4_) and concentrated in vacuo. Purification by flash chromatography eluting with 35% ethyl acetate in hexane gave methyl (2*S*)-2-acetylamino-3-(2’-[4″-fluorophenyl]­ethynyl-1′*H*-indol-3′-yl)­propanoate (**7c**) as an off-white solid (61.0 mg, 62%). Mp 60**–**65 °C; IR (neat) 3253, 2951, 1733, 1651, 1503, 1220, 835, 741 cm^–1^; [α]_D_^22^ +53.0 (*c* 0.1, CHCl_3_); ^1^H NMR (400 MHz, CDCl_3_) δ 8.35 (br s, 1H), 7.58–7.53 (m, 2H), 7.51 (d, *J* = 8.1 Hz, 1H), 7.31 (d, *J* = 8.1 Hz, 1H), 7.24 (ddd, *J* = 8.1, 7.0, 1.1 Hz, 1H), 7.13 (ddd, *J* = 8.1, 7.0, 1.1 Hz, 1H), 7.11–7.05 (m, 2H), 6.13 (d, *J* = 7.9 Hz, 1H), 4.98 (ddd, *J* = 7.9, 5.6, 5.0 Hz, 1H), 3.64 (s, 3H), 3.49 (dd, *J* = 14.4, 5.6 Hz, 1H), 3.45 (dd, *J* = 14.4, 5.0 Hz, 1H), 1.92 (s, 3H); ^13^C­{^1^H} NMR (101 MHz, CDCl_3_) δ 172.3, 169.8, 162.9 (d, ^1^ *J*_C–F_ = 250.6 Hz), 136.1 (d, ^3^ *J*_C–F_ = 8.4 Hz), 133.4, 127.5, 124.1, 120.6, 119.1, 118.6 (d, ^4^ *J*_C–F_ = 3.5 Hz), 118.1, 116.3, 116.1 (d, ^2^ *J*_C–F_ = 22.2 Hz), 111.2, 94.2, 80.7, 52.9, 52.7, 27.8, 23.4; HRMS (ESI) *m*/*z*: [M + H]^+^ calcd for C_22_H_19_FN_2_O_3_H 379.1452; found 379.1458.

### Methyl (2*S*)-2-Acetylamino-3-(2’-[4″-cyanophenyl]­ethynyl-1′*H*-indol-3′-yl)­propanoate (**7d**)

To an oven-dried microwave vial were added (2*S*)-*N*-acetyl-2′-iodotryptophan methyl ester (**6**) (100 mg, 0.259 mmol), 4-ethynylbenzonitrile (32.9 mg, 0.259 mmol), bis­(triphenylphosphine)­palladium­(II) dichloride (9.09 mg, 0.0130 mmol), and copper­(I) iodide (4.93 mg, 0.0259 mmol). The microwave vial was sealed and placed under an argon atmosphere. To the vial was added a degassed mixture of triethylamine (10.5 mL) and *N*,*N*-dimethylformamide (4.5 mL). The reaction mixture was stirred at 90 °C for 0.5 h, then allowed to cool to room temperature, and stirred for 3 h. Following the addition of the second batch of bis­(triphenylphosphine)­palladium­(II) dichloride (9.09 mg, 0.0130 mmol) and copper­(I) iodide (4.93 mg, 0.0259 mmol), the reaction mixture was stirred at 90 °C for 0.5 h, then allowed to cool to room temperature, and stirred for 16 h. The reaction mixture was concentrated in vacuo, and the resulting residue was dissolved in ethyl acetate (20 mL) and washed with water (2 × 20 mL) and brine (20 mL). The combined organic layers were dried (MgSO_4_) and concentrated in vacuo. Purification by flash chromatography eluting with 45% ethyl acetate in hexane gave methyl (2*S*)-2-acetylamino-3-(2’-[4″-cyanophenyl]­ethynyl-1′*H*-indol-3′-yl)­propanoate (**7d**) as a yellow solid (66.6 mg, 67%). Mp 220–221 °C; IR (neat) 3356, 3311, 2947, 2195, 1737, 1650, 1602, 1547, 1374, 1171, 835, 740 cm^–1^; [α]_D_^23^ +23.9 (*c* 0.1, CHCl_3_); ^1^H NMR (400 MHz, CDCl_3_) δ 8.41 (br s, 1H), 7.65–7.64 (m, 4H), 7.52 (d, *J* = 8.1 Hz, 1H), 7.32 (dt, *J* = 8.1, 1.1 Hz, 1H), 7.27 (ddd, *J* = 8.1, 6.9, 1.1 Hz, 1H), 7.15 (ddd, *J* = 8.1, 6.9, 1.1 Hz, 1H), 6.10 (d, *J* = 7.9 Hz, 1H), 5.02–4.96 (m, 1H), 3.64 (s, 3H), 3.52 (dd, *J* = 14.3, 5.8 Hz, 1H), 3.47 (dd, *J* = 14.3, 4.8 Hz, 1H), 1.91 (s, 3H); ^13^C­{^1^H} NMR (101 MHz, CDCl_3_) δ 172.2, 169.7, 136.4, 132.4, 131.7, 127.42, 127.38, 124.7, 120.8, 119.4, 118.5, 118.1, 117.3, 111.9, 111.4, 93.9, 85.5, 52.9, 52.7, 28.0, 23.4; HRMS (ESI) *m*/*z*: [M + H]^+^ calcd for C_23_H_19_N_3_O_3_H 386.1499; found 386.1501.

### Methyl (2*S*)-2-Acetylamino-3-(2′-phenyl-1′*H*-indol-3′-yl)­propanoate (**8a**).^7^

A solution of (2*S*)-*N*-acetyl-2′-iodotryptophan methyl ester (**6**) (0.050 g, 0.13 mmol), phenyl boronic acid (0.024 g, 0.19 mmol), and potassium phosphate (0.055 g, 0.26 mmol) in tetrahydrofuran/water (1:1, 4 mL) in a microwave vial was degassed under argon for 0.1 h. XPhos Pd G2 (0.0020 g, 0.0026 mmol) was added to the solution. The reaction mixture was heated at 60 °C for 3 h. After cooling to room temperature, the reaction mixture was concentrated in vacuo and diluted with water (5 mL). The mixture was extracted with ethyl acetate (3 × 10 mL). The combined organic layers were dried (MgSO_4_), filtered, and concentrated in vacuo. Purification by flash column chromatography eluting with 50% ethyl acetate in hexane gave methyl (2*S*)-2-acetylamino-3-(2′-phenyl-1′*H*-indol-3′-yl)­propanoate (**8a**) as a white solid (0.029 g, 66%). Spectroscopic data were consistent with the literature.^7^ [α]_D_^19^ +65.7 (*c* 0.11, CHCl_3_); ^1^H NMR (400 MHz, CDCl_3_) δ 8.27 (br s, 1H), 7.60–7.54 (m, 3H), 7.47 (t, *J* = 7.6 Hz, 2H), 7.41–7.31 (m, 2H), 7.20 (t, *J* = 7.8 Hz, 1H), 7.14 (t, *J* = 7.8 Hz, 1H), 5.78 (d, *J* = 8.1 Hz, 1H), 4.84 (ddd, *J* = 8.1, 5.5, 5.2 Hz, 1H), 3.57 (dd, *J* = 14.8, 5.5 Hz, 1H), 3.53 (dd, *J* = 14.8, 5.2 Hz, 1H), 3.30 (s, 3H), 1.66 (s, 3H); ^13^C­{^1^H} NMR (101 MHz, CDCl_3_) δ 172.3, 169.7, 136.1, 135.8, 133.3, 129.6, 129.3, 128.4, 128.2, 122.7, 120.2, 119.0, 111.1, 106.9, 52.9, 52.1, 26.7, 23.0; MS (ESI) *m/z:* 359 (M + Na^+^, 100).

### Methyl (2*S*)-2-Acetylamino-3-(2’-[4″-methoxyphenyl]-1′*H*-indol-3′-yl)­propanoate (**8b**).^7^

A solution of (2*S*)-*N*-acetyl-2′-iodotryptophan methyl ester (**6**) (0.060 g, 0.16 mmol), (4-methoxyphenyl)­boronic acid (0.038 g, 0.25 mmol), and potassium phosphate (0.068 g, 0.32 mmol) in tetrahydrofuran/water (1:1, 4 mL) in a microwave vial was degassed under argon for 0.1 h. XPhos Pd G2 (0.0027 g, 0.0030 mmol) was added to the solution. The reaction mixture was heated at 60 °C for 1.2 h. After being cooled to room temperature, the reaction mixture was concentrated in vacuo and diluted with water (6 mL). The mixture was extracted with ethyl acetate (3 × 10 mL). The combined organic layers were dried (MgSO_4_), filtered, and concentrated in vacuo. Purification by flash column chromatography eluting with 30% hexane in ethyl acetate gave methyl (2*S*)-2-acetylamino-3-(2’-[4″-methoxyphenyl]-1′*H*-indol-3′-yl)­propanoate (**8b**) as a white solid (0.043 g, 76%). Mp 200–203 °C (lit.^7^ 202–205 °C); [α]_D_^17^ +45.7 (*c* 0.1, CHCl_3_); ^1^H NMR (400 MHz, CDCl_3_) δ 8.48 (br s, 1H), 7.55 (d, *J* = 7.4 Hz, 1H), 7.44 (d, *J* = 8.3 Hz, 2H), 7.32 (d, *J* = 7.4 Hz, 1H), 7.16 (t, *J* = 7.4, 1H), 7.11 (t, *J* = 7.4 Hz, 1H), 6.94 (d, *J* = 8.4 Hz, 2H), 5.84 (d, *J* = 8.0 Hz, 1H), 4.82 (dt, *J* = 8.0, 5.5 Hz, 1H), 3.82 (s, 3H), 3.48 (d, *J* = 5.5 Hz, 2H), 3.34 (s, 3H), 1.67 (s, 3H); ^13^C­{^1^H} NMR (101 MHz, CDCl_3_) δ 172.4, 169.8, 159.5, 136.1, 135.7, 129.7, 129.5, 125.6, 122.3, 119.9, 118.7, 114.6, 111.0, 106.0, 55.5, 53.0, 52.2, 26.8, 23.0; MS (ESI) *m/z:* 389 (M + Na^+^, 100).

### Methyl (2*S*)-2-Acetylamino-3-(2’-[4″-dimethylaminophenyl]-1′*H*-indol-3′-yl)­propanoate (**8c**)

A solution of (2*S*)-*N*-acetyl-2′-iodotryptophan methyl ester (**6**) (0.065 g, 0.17 mmol), (4-dimethylaminophenyl)­boronic acid (0.042 g, 0.26 mmol), and potassium phosphate (0.078 g, 0.37 mmol) in tetrahydrofuran/water (1:1, 4 mL) in a microwave vial was degassed under argon for 0.1 h. XPhos Pd G2 (0.0028 g, 0.0040 mmol) was added to the solution. The reaction mixture was heated at 60 °C for 1.2 h. After cooling to room temperature, the reaction mixture was concentrated in vacuo and diluted with water (6 mL). The mixture was extracted with ethyl acetate (3 × 10 mL). The combined organic layers were dried (MgSO_4_), filtered, and concentrated in vacuo. Purification by flash column chromatography eluting with a 10% hexane in ethyl acetate gave methyl (2*S*)-2-acetylamino-3-(2’-[4″-dimethylaminophenyl]-1′*H*-indol-3′-yl)­propanoate (**8c**) as an orange solid (0.053 g, 83%). Mp 108–110 °C; IR (neat) 3253, 2950, 1738, 1653, 1609, 1530, 1218, 820, 744 cm^–1^; [α]_D_^19^ +19.3 (*c* 0.1, CHCl_3_); ^1^H NMR (400 MHz, CDCl_3_) δ 8.44 (br s, 1H), 7.53 (d, *J* = 7.7 Hz, 1H), 7.39 (d, *J* = 8.6 Hz, 2H), 7.30 (d, *J* = 7.7 Hz, 1H), 7.19–7.07 (m, 2H), 6.74 (d, *J* = 8.4 Hz, 2H), 5.84 (d, *J* = 8.0 Hz, 1H), 4.82 (dt, *J* = 8.0, 5.6 Hz, 1H), 3.49 (d, *J* = 5.6 Hz, 2H), 3.37 (s, 3H), 2.98 (s, 6H), 1.66 (s, 3H); ^13^C­{^1^H} NMR (101 MHz, CDCl_3_) δ 172.5, 169.8, 150.2, 136.9, 135.6, 129.6, 129.2, 121.8, 120.8, 119.7, 118.4, 112.7, 110.9, 105.2, 53.0, 52.2, 40.5, 26.7, 23.0; HRMS (ESI) *m/z*: [M + H]^+^ calcd for C_22_H_25_N_3_O_3_H 380.1969; found 380.1977.

### Methyl (2*S*)-2-Acetylamino-3-(2’-[4″-acetylphenyl]-1′*H*-indol-3′-yl)­propanoate (**8d**)

A solution of (2*S*)-*N*-acetyl-2′-iodotryptophan methyl ester (**6**) (0.12 g, 0.30 mmol), 4-acetylphenyl boronic acid (0.077 g, 0.47 mmol), and potassium phosphate (0.13 g, 0.60 mmol) in tetrahydrofuran/water (1:1, 8 mL) in a microwave vial was degassed under argon for 0.1 h. XPhos Pd G2 (0.0049 g, 0.0060 mmol) was added to the solution. The reaction mixture was heated at 60 °C for 1.2 h. After being cooled to room temperature, the reaction mixture was concentrated in vacuo and diluted with water (15 mL). The mixture was extracted with ethyl acetate (3 × 20 mL). The combined organic layers were dried (MgSO_4_), filtered, and concentrated in vacuo. Purification by flash column chromatography eluting with a gradient system of 20% to 50% ethyl acetate in diethyl ether gave methyl (2*S*)-2-acetylamino-3-(2’-[4″-acetylphenyl]-1′*H*-indol-3′-yl)­propanoate (**8d**) as a yellow solid (0.074 g, 65%). Mp 172–174 °C; IR (neat) 3316, 3058, 1741, 1679, 1638, 1439, 1180, 988, 744 cm^–1^; [α]_D_^19^ +52.2 (*c* 0.1, CHCl_3_); ^1^H NMR (400 MHz, CDCl_3_) δ 9.13 (br s, 1H), 7.91 (d, *J* = 8.0 Hz, 2H), 7.59 (d, *J* = 8.0 Hz, 2H), 7.56 (d, *J* = 7.7 Hz, 1H), 7.31 (d, *J* = 7.7 Hz, 1H), 7.18 (t, *J* = 7.7 Hz, 1H), 7.11 (t, *J* = 7.7 Hz, 1H), 6.03 (d, *J* = 7.9 Hz, 1H), 4.88–4.80 (m, 1H), 3.56–3.44 (m, 2H), 3.31 (s, 3H), 2.55 (s, 3H), 1.65 (s, 3H); ^13^C­{^1^H} NMR (101 MHz, CDCl_3_) δ 197.7, 172.2, 169.9, 137.9, 136.3, 135.8, 134.6, 129.4, 129.0, 128.1, 123.1, 120.1, 119.0, 111.4, 108.2, 53.0, 52.2, 27.2, 26.7, 22.9; HRMS (ESI) *m*/*z*: [M + Na]^+^ calcd for C_22_H_22_N_2_O_4_Na 401.1472; found 401.1467.

### Methyl (2*S*)-2-Acetylamino-3-(2’-[4″-cyanophenyl]-1′*H*-indol-3′-yl)­propanoate (**8e**)

A solution of (2*S*)-*N*-acetyl-2′-iodotryptophan methyl ester (**6**) (0.12 g, 0.30 mmol), (4-cyanophenyl)­boronic acid (0.066 g, 0.45 mmol), and potassium phosphate (0.13 g, 0.60 mmol) in tetrahydrofuran/water (1:1, 8 mL) in a microwave vial was degassed under argon for 0.1 h. XPhos Pd G2 (0.0050 g, 0.0064 mmol) was added to the solution. The reaction mixture was heated at 60 °C for 1.25 h. Another portion of XPhos Pd G2 (0.0020 g, 0.0025 mmol) was added, and the reaction mixture was left stirring at 60 °C for 2 h. After being cooled to room temperature, the reaction mixture was concentrated in vacuo and diluted with water (10 mL). The mixture was extracted with ethyl acetate (3 × 10 mL). The combined organic layers were dried (MgSO_4_), filtered, and concentrated in vacuo. Purification by flash column chromatography eluting with 20% dichloromethane in ethyl acetate gave methyl (2*S*)-2-acetylamino-3-(2′-[4″-cyanophenyl]-1′*H*-indol-3′-yl)­propanoate (**8e**) as a white solid (0.071 g, 65%). Mp 196–201 °C; IR (neat) 3285, 2952, 2224, 1735, 1650, 1604, 1526, 1437, 1215, 840, 744 cm^–1^; [α]_D_^19^ +49.5 (*c* 0.1, CHCl_3_); ^1^H NMR (400 MHz, CDCl_3_) δ 8.25 (br s, 1H), 7.76 (d, *J* = 8.6 Hz, 2H), 7.71 (d, *J* = 8.6 Hz, 2H), 7.60 (d, *J* = 7.8 Hz, 1H), 7.38 (d, *J* = 7.8 Hz, 1H), 7.28–7.22 (m, 1H), 7.19–7.14 (m, 1H), 5.88 (d, *J* = 7.9 Hz, 1H), 4.86 (ddd, *J* = 7.9, 6.1, 5.3 Hz, 1H), 3.55 (dd, *J* = 14.7, 6.1 Hz, 1H), 3.51 (dd, *J* = 14.7, 5.3 Hz, 1H), 3.34 (s, 3H), 1.73 (s, 3H); ^13^C­{^1^H} NMR (101 MHz, CDCl_3_) δ 172.2, 169.6, 137.8, 136.3, 133.7, 132.9, 129.5, 128.6, 123.8, 120.7, 119.5, 118.6, 111.4, 109.4, 53.0, 52.3, 27.3, 23.1; HRMS (ESI) *m*/*z*: [M + H]^+^ calcd for C_21_H_19_N_3_O_3_H 362.1499; found 362.1500.

### Methyl (2*S*)-2-Acetylamino-3-(2’-[4″-fluorophenyl]-1′*H*-indol-3′-yl)­propanoate (**8f**).^7^

A solution of (2*S*)-*N*-acetyl-2′-iodotryptophan methyl ester (**6**) (0.092 g, 0.24 mmol), (4-fluorophenyl)­boronic acid (0.050 g, 0.36 mmol), and potassium phosphate (0.10 g, 0.49 mmol) in tetrahydrofuran/water (1:1, 8 mL) in a microwave vial was degassed under argon for 0.1 h. XPhos Pd G2 (0.0037 g, 0.0050 mmol) was added to the solution. The reaction mixture was heated at 60 °C for 1.2 h. After it was cooled to room temperature, the reaction mixture was concentrated in vacuo and diluted with water (12 mL). The mixture was extracted with ethyl acetate (3 × 20 mL). The combined organic layers were dried (MgSO_4_), filtered, and concentrated in vacuo. Purification by flash column chromatography eluting with 25% ethyl acetate in dichloromethane, followed by recrystallization from chloroform and hexane, gave methyl (2*S*)-2-acetylamino-3-(2′-[4″-fluorophenyl]-1′*H*-indol-3′-yl)­propanoate (**8f**) as a white solid (0.065 g, 79%). Spectroscopic data were consistent with the literature.^7^ Mp 45–48 °C; [α]_D_^19^ +46.9 (*c* 0.1, CHCl_3_); ^1^H NMR (400 MHz, CDCl_3_) δ 8.49 (br s, 1H), 7.56 (d, *J* = 7.7 Hz, 1H), 7.51–7.45 (m, 2H), 7.33 (d, *J* = 7.7 Hz, 1H), 7.20 (t, *J* = 7.7 Hz, 1H), 7.15–7.06 (m, 3H), 5.85 (d, *J* = 8.0 Hz, 1H), 4.82 (dt, *J* = 8.0, 5.6 Hz, 1H), 3.49 (dd, *J* = 14.8, 5.6 Hz, 1H), 3.44 (dd, *J* = 14.8, 5.6 Hz, 1H), 3.33 (s, 3H), 1.70 (s, 3H); ^13^C­{^1^H} NMR (101 MHz, CDCl_3_) δ 172.3, 169.8, 162.5 (d, ^1^ *J*_CF_ = 249.4 Hz), 135.8, 135.1, 130.2 (d, ^3^ *J*_CF_ = 8.0 Hz), 129.4, 129.3 (d, ^4^ *J*_CF_ = 3.4 Hz), 122.7, 120.1, 118.9, 116.2 (d, ^2^ *J*_CF_ = 21.2 Hz), 111.2, 106.8, 53.0, 52.2, 26.8, 23.0; MS (ESI) *m/z:* 377 (M + Na^+^, 100).

### Methyl (2*S*)-2-Acetylamino-3-(2′-[4″-trifluoromethylphenyl]-1′*H*-indol-3′-yl)­propanoate (**8g**).^7^

A solution of (2*S*)-*N*-acetyl-2′-iodotryptophan methyl ester (**6**) (0.065 g, 0.17 mmol), (4-trifluoromethylphenyl)­boronic acid (0.049 g, 0.26 mmol), and potassium phosphate (0.074 g, 0.35 mmol) in tetrahydrofuran/water (1:1, 4 mL) in a microwave vial was degassed under argon for 0.1 h. XPhos Pd G2 (0.0026 g, 0.0030 mmol) was added to the solution. The reaction mixture was heated at 60 °C for 1.2 h. After cooling to room temperature, the reaction mixture was concentrated in vacuo and diluted with water (6.5 mL). The mixture was extracted with ethyl acetate (3 × 10 mL). The combined organic layers were dried (MgSO_4_), filtered, and concentrated in vacuo. Purification by flash column chromatography eluting with 40% ethyl acetate in hexane gave methyl (2*S*)-2-acetylamino-3-(2′-[4″-trifluoromethylphenyl]-1′*H*-indol-3′-yl)­propanoate (**8g**) as a white solid (0.037 g, 55%). Spectroscopic data were consistent with the literature.^7^ [α]_D_^19^ +67.1 (*c* 0.13, CHCl_3_); ^1^H NMR (400 MHz, CDCl_3_) δ 8.67 (br s, 1H), 7.67–7.61 (m, 4H), 7.58 (d, *J* = 7.8 Hz, 1H), 7.32 (d, *J* = 7.8 Hz, 1H), 7.23 (t, *J* = 7.8 Hz, 1H), 7.15 (t, *J* = 7.8 Hz, 1H), 5.90 (d, *J* = 8.1 Hz, 1H), 4.86 (dt, *J* = 8.1, 5.6 Hz, 1H), 3.55 (dd, *J* = 14.8, 5.6 Hz, 1H), 3.50 (dd, *J* = 14.8, 5.6 Hz, 1H), 3.29 (s, 3H), 1.66 (s, 3H); ^13^C­{^1^H} NMR (101 MHz, CDCl_3_) δ 172.2, 169.8, 136.8, 136.1, 134.3, 129.7 (q, ^2^ *J*_CF_ = 32.7 Hz), 129.5, 128.5, 126.0 (q, ^3^ *J*_CF_ = 3.7 Hz), 123.9 (q, ^1^ *J*_CF_ = 273 Hz), 123.3, 120.4, 119.1, 111.4, 108.1, 53.0, 52.2, 26.9, 23.0; MS (ESI) *m/z:* 405 (M + H^+^, 100).

### Methyl (2*S*)-2-Acetylamino-3-(2′-[2″-naphthyl]-1′*H*-indol-3′-yl)­propanoate (**8h**)

A solution of (2*S*)-*N*-acetyl-2′-iodotryptophan methyl ester (**6**) (0.12 g, 0.30 mmol), 2-naphthylboronic acid (0.079 g, 0.46 mmol), and potassium phosphate (0.13 g, 0.60 mmol) in tetrahydrofuran/water (1:1, 7 mL) in a microwave vial was degassed under argon for 0.1 h. XPhos Pd G2 (0.0048 g, 0.0060 mmol) was added to the solution. The reaction mixture was heated at 60 °C for 1.2 h. After cooling to room temperature, the reaction mixture was concentrated in vacuo and diluted with water (12 mL). The mixture was extracted with ethyl acetate (3 × 20 mL). The combined organic layers were dried (MgSO_4_), filtered, and concentrated in vacuo. Purification by flash column chromatography eluting with 80% dichloromethane in ethyl acetate gave methyl (2*S*)-2-acetylamino-3-(2’-[2″-naphthyl]-1′*H*-indol-3′-yl)­propanoate (**8h**) as a white solid (0.070 g, 60%). Mp 92–94 °C; IR (neat) 3268, 2951, 1735, 1653, 1513, 1435, 1217, 744 cm^–1^; [α]_D_^20^ +61.7 (*c* 0.1, CHCl_3_); ^1^H NMR (400 MHz, CDCl_3_) δ 8.56 (br s, 1H), 8.01 (d, *J* = 1.8 Hz, 1H), 7.91–7.82 (m, 3H), 7.64 (dd, *J* = 8.5, 1.8 Hz, 1H), 7.59 (d, *J* = 7.9 Hz, 1H), 7.55–7.49 (m, 2H), 7.36 (d, *J* = 7.9 Hz, 1H), 7.24–7.18 (m, 1H), 7.15 (t, *J* = 7.9 Hz, 1H), 5.83 (d, *J* = 8.0 Hz, 1H), 4.86 (dt, *J* = 8.0, 5.4 Hz, 1H), 3.61 (dd, *J* = 14.8, 5.4 Hz, 1H), 3.56 (dd, *J* = 14.8, 5.4 Hz, 1H), 3.17 (s, 3H), 1.52 (s, 3H); ^13^C­{^1^H} NMR (101 MHz, CDCl_3_) δ 172.3, 169.7, 136.0, 133.6, 132.8, 130.6, 129.6, 128.9, 128.1, 127.8, 127.2, 126.9, 126.7, 126.0, 122.7, 120.1, 119.0, 111.2, 107.3, 53.0, 52.1, 27.0, 22.9; HRMS (ESI) *m*/*z*: [M + Na]^+^ calcd for C_24_H_22_N_2_O_3_Na 409.1523; found 409.1519.

### Methyl (2*S*)-2-Acetylamino-3-(2’-[biphen-4″-yl]-1′*H*-indol-3′-yl)­propanoate (**8i**)

A solution of (2*S*)-*N*-acetyl-2′-iodotryptophan methyl ester (**6**) (0.050 g, 0.13 mmol), 4-biphenyl boronic acid (0.066 g, 0.45 mmol), and potassium phosphate (0.038 g, 0.19 mmol) in tetrahydrofuran/water (1:1, 4 mL) in a microwave vial was degassed under argon for 0.1 h. XPhos Pd G2 (0.0020 g, 0.0026 mmol) was added to the solution. The reaction mixture was heated at 60 °C for 2 h. After cooling to room temperature, the reaction mixture was concentrated in vacuo and diluted with water (6 mL). The mixture was extracted with ethyl acetate (3 × 10 mL). The combined organic layers were dried (MgSO_4_), filtered, and concentrated in vacuo. Purification by flash column chromatography eluting with 50% ethyl acetate in hexane gave methyl (2*S*)-2-acetylamino-3-(2’-[biphen-4″-yl]-1′*H*-indol-3′-yl)­propanoate (**8i**) as a white solid (0.045 g, 75%). Mp 205–207 °C; IR (neat) 3248, 2952, 1734, 1654, 1435, 1213, 843, 742 cm^–1^; [α]_D_^17^ +43.3 (*c* 0.1, CHCl_3_); ^1^H NMR (400 MHz, CDCl_3_) δ 8.27 (br s, 1H), 7.72 (d, *J* = 8.2 Hz, 2H), 7.68–7.61 (m, 4H), 7.60 (d, *J* = 7.8 Hz, 1H), 7.51–7.45 (m, 2H), 7.42–7.36 (m, 2H) 7.24–7.19 (m, 1H), 7.18–7.13 (m, 1H), 5.81 (d, *J* = 8.0 Hz, 1H), 4.88 (ddd, *J* = 8.0, 5.6, 5.3 Hz, 1H), 3.61 (dd, *J* = 14.9, 5.6 Hz, 1H), 3.57 (dd, *J* = 14.9, 5.2 Hz, 1H), 3.32 (s, 3H), 1.67 (s, 3H); ^13^C­{^1^H} NMR (101 MHz, CDCl_3_) δ 172.3, 169.7, 140.9, 140.3, 135.9, 135.7, 132.2, 129.7, 129.1, 128.7, 127.9, 127.1, 122.8, 120.2, 119.0, 111.1, 107.2, 53.0, 52.2, 26.9, 23.0; HRMS (ESI) *m*/*z*: [M + Na]^+^ calcd for C_26_H_24_N_2_O_3_Na 435.1679; found 435.1678.

### Methyl (2*S*)-2-Acetylamino-3-(2’-[dibenzo­[*b*,*d*]­thiophen-3′’-yl]-1′*H*-indol-3′-yl)­propanoate (**8j**)

A microwave vial charged with (2*S*)-*N*-acetyl-2′-iodotryptophan methyl ester (**6**) (0.10 g, 0.26 mmol), dibenzo­[*b*,*d*]­thiophen-3-ylboronic acid (0.089 g, 0.39 mmol), XPhos Pd G2 (0.0041 g, 0.0052 mmol), and potassium phosphate (0.11 g, 0.52 mmol) was placed under an argon atmosphere. To the vial was added a degassed mixture of tetrahydrofuran/water (1:1, 8.6 mL), and the resulting reaction mixture was degassed under argon for 0.1 h. The reaction mixture was stirred at 60 °C for 2 h. After cooling to room temperature, the reaction mixture was concentrated in vacuo, dissolved in ethyl acetate (20 mL), and washed with water (2 × 10 mL) and brine (10 mL). The organic layer was dried (MgSO_4_), filtered, and concentrated in vacuo. Purification by flash column chromatography eluting with 40–50% ethyl acetate in hexane gave methyl (2*S*)-2-acetylamino-3-(2’-[dibenzo­[b,d]­thiophen-3′’-yl]-1′H-indol-3′-yl)­propanoate (**8j**) as a gray solid (0.094 g, 81%). Mp 94–96 °C; IR (neat) 3256, 2949, 1732, 1651, 1432, 1213, 733 cm^–1^; [α]_D_^20^ +64.3 (c 0.1, CHCl_3_); ^1^H NMR (400 MHz, CDCl_3_) δ 8.55 (s, 1H), 8.15–8.10 (m, 2H) 8.01 (br s, 1H), 7.90–7.84 (m, 1H), 7.61–7.56 (m, 2H), 7.53–7.46 (m, 2H), 7.34 (dt, *J* = 7.1, 0.9 Hz, 1H), 7.21 (td, *J* = 7.1, 0.9 Hz, 1H), 7.15 (td, *J* = 7.1, 0.9 Hz, 1H), 5.86 (d, *J* = 8.0 Hz, 1H), 4.86 (ddd, *J* = 8.0, 5.8, 5.4 Hz, 1H), 3.59 (dd, *J* = 14.9, 5.8 Hz, 1H), 3.55 (dd, *J* = 14.9, 5.4 Hz, 1H), 3.21 (s, 3H), 1.59 (s, 3H); ^13^C­{^1^H} NMR (101 MHz, CDCl_3_) δ 172.3, 169.8, 140.3, 139.9, 136.0, 135.7, 135.08, 135.05, 131.7, 129.6, 127.2, 124.8, 124.7, 123.0, 122.8, 122.3, 122.1, 121.9, 120.2, 119.0, 111.2, 107.4, 53.0, 52.1, 26.9, 23.0; HRMS (ESI) *m*/*z*: [M + Na]^+^ calcd for C_26_H_22_N_2_O_3_SNa 465.1243; found 465.1265.

### Methyl (2*S*)-2-Acetylamino-3-(2’-[carbazole-2″-yl]-1′*H*-indol-3′-yl)­propanoate (**8k**)

A microwave vial charged with (2*S*)-*N*-acetyl-2′-iodotryptophan methyl ester (**6**) (0.10 g, 0.26 mmol), 2-(4,4,5,5-tetramethyl-1,3,2-dioxaborolan-2-yl)-9H-carbazole (0.11 g, 0.39 mmol), XPhos Pd G2 (0.0041 g, 0.0052 mmol), and potassium phosphate (0.11 g, 0.52 mmol) was placed under an argon atmosphere. To the vial was added a degassed mixture of tetrahydrofuran/water (1:1, 8.6 mL), and the resulting reaction mixture was degassed under argon for 0.1 h. The reaction mixture was stirred at 60 °C for 2 h. After cooling to room temperature, the reaction mixture was concentrated in vacuo, dissolved in ethyl acetate (20 mL), and washed with water (2 × 10 mL) and brine (10 mL). The organic layer was dried (MgSO_4_), filtered, and concentrated in vacuo. Purification by flash column chromatography eluting with 20–50% ethyl acetate in chloroform gave a light brown residue, which was recrystallized from chloroform/hexane to give methyl (2*S*)-2-acetylamino-3-(2’-[carbazole-2″-yl]-1′H-indol-3′-yl)­propanoate (**8k**) as a light brown solid (0.076 g, 69%). Mp 99–101 °C; IR (neat) 3390, 2950, 1731, 1651, 1455, 1440, 1235, 1215, 729 cm^–1^; [α]_D_^20^ +63.0 (c 0.1, CHCl_3_); ^1^H NMR (400 MHz, CDCl_3_) δ 8.99 (s, 1H), 8.08 (s, 1H), 7.99 (d, *J* = 7.8 Hz, 1H), 7.87 (d, *J* = 7.9 Hz, 1H), 7.54 (d, *J* = 7.5 Hz, 1H), 7.41 (t, *J* = 7.5 Hz, 1H), 7.28 (d, *J* = 7.9 Hz, 1H), 7.24–7.12 (m, 6H), 5.95 (d, *J* = 7.8 Hz, 1H), 4.81 (dt, *J* = 7.8, 5.4 Hz, 1H), 3.52 (dd, *J* = 14.9, 5.4 Hz, 1H), 3.45 (dd, *J* = 14.9, 5.4 Hz, 1H), 3.12 (s, 3H), 1.37 (s, 3H); ^13^C­{^1^H} NMR (101 MHz, CDCl_3_) δ 172.4, 170.4, 140.2, 139.8, 137.3, 135.8, 130.3, 129.6, 126.2, 122.8, 122.7, 122.3, 120.7, 120.4, 119.9, 119.6, 118.6, 111.4, 111.0, 110.4, 106.3, 53.4, 52.1, 26.7, 22.6; HRMS (ESI) *m*/*z*: [M + Na]^+^ calcd for C_26_H_23_N_3_O_3_Na 448.1632; found 448.1655.

### (2*S*)-2-Acetylamino-3-(2′-iodo-1′*H*-indol-3′-yl)­propanamide- *l* -alanine *tert*-butyl Ester (**9**)

A mixture of methyl (2*S*)-2-acetylamino-3-(2′-iodo-1′*H*-indol-3′-yl)­propanoate (**6**) (0.10 g, 0.26 mmol) and cesium carbonate (0.11 g, 0.34 mmol) was dissolved in methanol (4.3 mL), water (2.2 mL) and dioxane (2.2 mL). The solution was heated at 60 °C for 15 h. After it was cooled to room temperature, the reaction mixture was concentrated in vacuo. After diluting the resultant mixture with water (10 mL), the solution was acidified to pH 1 with 1 M hydrochloric acid. The mixture was extracted with ethyl acetate (3 × 10 mL). The combined organic layers were dried (MgSO_4_), filtered, and concentrated in vacuo to yield (2*S*)-2-acetylamino-3-(2′-iodo-1′*H*-indol-3′-yl)­propanoic acid (0.084 g, 87%). Without further purification, a mixture of (2*S*)-2-acetylamino-3-(2′-iodo-1′*H*-indol-3′-yl)­propanoic acid (0.060 g, 0.16 mmol), l-alanine *tert*-butyl ester hydrochloride (0.032 g, 0.18 mmol), and 1-hydroxybenzotriazole hydrate (0.013 g, 0.081 mmol) were dissolved in acetonitrile (4.0 mL) and stirred at 0 °C for 5 min prior to the addition of *N*,*N*-diisopropylethylamine (0.084 mL, 0.48 mmol) and PyBOP (0.13 g, 0.24 mmol). After a further 0.5 h, the reaction mixture was warmed to room temperature, stirred for 17 h, and then concentrated in vacuo. The reaction mixture was diluted in ethyl acetate (20 mL) and washed with 1 M hydrochloric acid (2 × 10 mL) and then brine (10 mL). The organic layer was dried (MgSO_4_), filtered, and concentrated in vacuo. Purification by flash column chromatography eluting with 30–50% ethyl acetate in chloroform gave (2*S*)-2-acetylamino-3-(2′-iodo-1′*H*-indol-3′-yl)­propanamide- *l* -alanine *tert*-butyl ester (**9**) as a white solid (0.074 g, 92%). Mp 105–108 °C; IR (neat) 3275, 2975, 2918, 1730, 1645, 1516, 1147, 841, 742 cm^–1^; [α]_D_^20^ −17.3 (*c* 0.1, MeOH); ^1^H NMR (400 MHz, CD_3_OD) δ 7.57 (d, *J* = 8.2 Hz, 1H), 7.27 (d, *J* = 8.1 Hz, 1H), 7.04 (ddd, *J* = 8.1, 7.1, 1.3 Hz, 1H), 6.98 (ddd, *J* = 8.2, 7.1, 1.2 Hz, 1H), 4.70 (dd, *J* = 7.3, 6.5 Hz, 1H), 4.22 (q, *J* = 7.2 Hz, 1H), 3.19 (dd, *J* = 14.4, 6.5 Hz, 1H), 3.08 (dd, *J* = 14.4, 7.3 Hz, 1H), 1.90 (s, 3H), 1.42 (s, 9H), 1.26 (d, *J* = 7.2 Hz, 3H); ^13^C­{^1^H} NMR (101 MHz, CD_3_OD) δ 173.1, 172.9, 172.8, 140.6, 128.9, 122.7, 120.2, 118.8, 116.8, 111.6, 82.8, 81.2, 55.1, 50.4, 30.8, 28.2, 22.8, 17.8; HRMS (ESI) *m*/*z*: [M + Na]^+^ calcd for C_20_H_26_IN_3_O_4_Na 522.0860; found 522.0858.

### (2*S*)-2-Acetylamino-3-(2’-[biphen-4″-yl]-1′*H*-indol-3′-yl)­propanamide- *l* -alanine *tert*-butyl Ester (**10**)

To a microwave vial were added (2*S*)-2-acetylamino-3-(2′-iodo-1′*H*-indol-3′-yl)­propanamide- *l* -alanine *tert*-butyl ester (**9**) (0.049 g, 0.098 mmol), 4-biphenyl boronic acid (0.029 g, 0.147 mmol), XPhos Pd G2 (0.002 g, 0.002 mmol), and potassium phosphate (0.042 g, 0.20 mmol). The microwave vial was sealed and placed under an argon atmosphere. To the vial was added a degassed mixture of tetrahydrofuran (1.6 mL) and water (1.6 mL). The reaction mixture was stirred at 60 °C for 1 h. After cooling to room temperature, the reaction mixture was diluted with ethyl acetate (20 mL) and washed with water (2 × 10 mL) and brine (10 mL). The combined organic layers were dried (MgSO_4_), filtered, and concentrated in vacuo. Purification by flash column chromatography eluting with 30% ethyl acetate in dichloromethane gave (2*S*)-2-acetylamino-3-(2’-[biphen-4″-yl]-1′*H*-indol-3′-yl)­propanamide- *l* -alanine *tert*-butyl ester (**10**) as a gray solid (0.036 mg, 69%). Mp 189–192 °C. IR (neat) 3280, 2978, 2931, 1734, 1641, 1541, 1367, 1147, 741 cm^–1^; [α]_D_^20^ −17.3 (*c* 0.1,CHCl_3_); ^1^H NMR (400 MHz, CDCl_3_) δ 8.40 (br s, 1H), 7.75 (d, *J* = 8.0 Hz, 1H), 7.70–7.64 (m, 4H), 7.63–7.60 (m, 2H), 7.46 (t, *J* = 7.5 Hz, 2H), 7.39–7.35 (m, 2H), 7.20 (ddd, *J* 8.1, 7.0, 1.3 Hz, 1H), 7.14 (ddd, *J* = 8.0, 7.0, 1.1 Hz, 1H), 6.30 (d, *J* = 7.4 Hz, 1H), 6.15 (d, *J* = 7.0 Hz, 1H), 4.72 (ddd, *J* = 8.5, 7.4, 5.6 Hz, 1H), 4.07 (pent, *J* = 7.0 Hz, 1H), 3.51 (dd, *J* = 14.4, 5.6 Hz, 1H), 3.34 (dd, *J* = 14.4, 8.5 Hz, 1H), 1.83 (s, 3H), 1.31 (s, 9H), 1.20 (d, *J* = 7.0 Hz, 3H); ^13^C­{^1^H} NMR (101 MHz, CDCl_3_) δ 171.3, 170.4, 169.9, 140.7, 140.4, 136.1, 135.5, 131.8, 129.3, 129.0, 128.3, 127.9, 127.7, 127.1, 122.9, 120.2, 119.3, 111.1, 107.9, 82.0, 54.1, 49.1, 28.4, 27.9, 23.2, 18.7; HRMS (ESI) *m*/*z*: [M + Na]^+^ calcd for C_32_H_35_N_3_O_4_Na 548.2520; found 548.2535.

### (2*S*)-2-Amino-3-(2’-[4″-dimethylaminophenyl]-1′*H*-indol-3′-yl)­propanoic Acid Hydrochloride (**11**)

A mixture of methyl (2*S*)-2-acetylamino-3-(2’-[4″-dimethylaminophenyl]-1′*H*-indol-3′-yl)­propanoate (**8c**) (0.028 g, 0.074 mmol) and cesium carbonate (0.033 g, 0.10 mmol) was dissolved in methanol (0.8 mL), water (1 mL) and dioxane (2 mL). The solution was heated at 60 °C for 18 h. After cooling to room temperature, the reaction mixture was concentrated in vacuo. After diluting the resultant mixture with water (10 mL), the solution was acidified to pH 1 with 1 M hydrochloric acid. The mixture was extracted with 20% isopropyl alcohol in chloroform (3 × 50 mL). The combined organic layers were dried (MgSO_4_), filtered, and concentrated in vacuo. The resultant solids were dissolved in methanol and warmed to 40 °C. Diethyl ether was added dropwise to precipitate a brown solid. After cooling to room temperature, the supernatant was removed. The remaining brown solid was dried in vacuo to give (2*S*)-2-acetylamino-3-(2’-[4″-dimethylaminophenyl]-1′*H*-indol-3′-yl)­propanoic acid (0.025 g, 90%). Without further purification, (2*S*)-2-acetylamino-3-(2’-[4″-dimethylaminophenyl]-1′*H*-indol-3′-yl)­propanoic acid (0.017 g, 0.046 mmol) was suspended in 6 M hydrochloric solution (1.6 mL). The solution was heated under reflux for 4 h. After cooling to room temperature, the reaction mixture was concentrated in vacuo. The residue was dissolved in methanol (0.4 mL) and warmed to 40 °C. Diethyl ether (4 mL) was then added dropwise to give a black precipitate. After cooling to room temperature, the supernatant was removed. The remaining black solid was then dried in vacuo to give (2*S*)-2-amino-3-(2’-[4″-dimethylaminophenyl]-1′*H*-indol-3′-yl)­propanoic acid hydrochloride (**11**) (0.014 g, 93%). Mp > 300 °C (decomposition); IR (neat) 3207, 2919, 1727, 1605, 1523, 1462, 1124 1113, 747 cm^–1^; [α]_D_^19^ +36.7 (*c* 0.1, MeOH); ^1^H NMR (400 MHz, CD_3_OD) δ 7.92–7.78 (m, 4H), 7.68 (d, *J* = 7.8 Hz, 1H), 7.45 (d, *J* = 7.8 Hz, 1H), 7.21 (t, *J* = 7.8 Hz, 1H), 7.13 (t, *J* = 7.8 Hz, 1H), 4.18 (dd, *J* = 8.4, 6.4 Hz, 1H), 3.72 (dd, *J* = 15.2, 6.4 Hz, 1H), 3.48 (dd, *J* = 15.2, 8.4 Hz, 1H), 3.36 (s, 6H); ^13^C­{^1^H} NMR (101 MHz, CD_3_OD) δ 171.4, 143.4, 138.1, 136.0, 135.9, 131.5, 129.6, 123.9, 122.3, 121.0, 119.4, 112.6, 106.5, 54.4, 47.2, 27.3; HRMS (ESI) *m*/*z*: [M + H]^+^ calcd for C_19_H_21_N_3_O_2_H 324.1707; found 324.1710.

### (2*S*)-2-Acetylamino-3-(2’-[biphen-4″-yl]-1′*H*-indol-3′-yl)­propanamide-l-tryptophan Methyl Ester (**13**)

A mixture of methyl (2*S*)-2-acetylamino-3-(2’-[biphen-4″-yl]-1′*H*-indol-3′-yl)­propanoate (**8i**) (0.10 g, 0.24 mmol) and cesium carbonate (0.10 g, 0.32 mmol) was dissolved in methanol (4.0 mL), water (2.0 mL), and dioxane (2.0 mL). The solution was heated at 60 °C for 18 h. After cooling to room temperature, the reaction mixture was concentrated in vacuo. After diluting the resultant mixture with water (10 mL), the solution was acidified to pH 1 with 1 M hydrochloric acid. The mixture was extracted with ethyl acetate (3 × 10 mL). The combined organic layers were dried (MgSO_4_), filtered, and concentrated in vacuo to yield (2*S*)-2-acetylamino-3-(2’-[biphen-4″-yl]-1′*H*-indol-3′-yl)­propanoic acid (**12**) (0.091 g, 95%). Without further purification, a mixture of (2*S*)-2-acetylamino-3-(2′-[biphen-4″-yl]-1′*H*-indol-3′-yl)­propanoic acid (0.057 g, 0.14 mmol), l-tryptophan methyl ester hydrochloride (0.040 g, 0.16 mmol), and 1-hydroxybenzotriazole hydrate (0.011 g, 0.072 mmol) was dissolved in acetonitrile (3.6 mL) and stirred at 0 °C for 5 min prior to the addition of *N*,*N*-diisopropylethylamine (0.075 mL, 0.43 mmol) and PyBOP (0.11 g, 0.22 mmol). After a further 0.5 h, the reaction mixture was warmed to room temperature, stirred for 19 h, and then concentrated in vacuo. The reaction mixture was diluted in ethyl acetate (20 mL) and washed with 1 M hydrochloric acid (2 × 10 mL) and then brine (10 mL). The organic layer was dried (MgSO_4_), filtered, and concentrated in vacuo. Purification by flash column chromatography eluting with 10% toluene in a 1:1 mixture of ethyl acetate/chloroform gave a white solid, which was recrystallized from chloroform/hexane to give (2*S*)-2-acetylamino-3-(2′-[biphen-4″-yl]-1′*H*-indol-3′-yl)­propanamide-l-tryptophan methyl ester (**13**) as a white solid (0.052 g, 61%). Mp 132–134 °C; IR (neat) 3295, 3029, 1736, 1647, 1507, 1486, 1434, 1214, 1008, 845, 741 cm^–1^; [α]_D_^19^ +30.3 (*c* 0.1, CHCl_3_); ^1^H NMR (400 MHz, CDCl_3_) δ 8.54 (s, 1H), 8.15 (d, *J* = 2.0 Hz, 1H), 7.68 (d, *J* = 8.0 Hz, 1H), 7.61–7.55 (m, 6H), 7.47–7.42 (m, 2H), 7.37 (tdd, *J* = 7.3, 2.2, 1.2 Hz, 1H), 7.32 (d, *J* = 8.0 Hz, 1H), 7.26 (d, *J* = 7.8 Hz, 1H), 7.22–7.16 (m, 2H), 7.13–7.06 (m, 2H), 6.97 (ddd, *J* = 7.8, 7.0, 0.8 Hz, 1H), 6.66 (d, *J* = 2.4 Hz, 1H), 6.21 (d, *J* = 7.2 Hz, 1H), 6.16 (d, *J* = 7.7 Hz, 1H), 4.74 (td, *J* = 7.7, 5.8 Hz, 1H), 4.52 (dt, *J* = 7.2, 5.1 Hz, 1H), 3.87 (dd, *J* = 14.7, 5.8 Hz, 1H), 3.37 (dd, *J* = 14.7, 7.7 Hz, 1H), 3.36 (s, 3H), 3.15–3.04 (m, 2H), 1.65 (s, 3H); ^13^C­{^1^H} NMR (101 MHz, CDCl_3_) δ 171.4, 170.9, 170.2, 140.6, 140.2, 136.1, 136.0, 135.6, 131.7, 129.4, 129.0, 128.4, 127.74, 127.70, 127.5, 127.1, 123.3, 122.7, 122.1, 120.2, 119.4, 119.2, 118.4, 111.4, 111.1, 109.3, 107.6, 54.0, 53.2, 52.3, 27.9, 27.4, 23.0; HRMS (ESI) *m*/*z*: [M + Na]^+^ calcd for C_37_H_34_N_4_O_4_Na 621.2472; found 621.2481.

## Supplementary Material



## Data Availability

The data underlying this study are available in the published article and its online Supporting Information.

## Notes

Photophysical data for α-amino acids **7a**–**d**, **8a**–**k**, and **11** and dipeptides **10** and **13**, and ^1^H and ^13^C NMR spectra for all compounds (PDF)
