# Supplementary material for: Palladium-Catalyzed C2 Functionalization of an Iodinated Tryptophan Scaffold for Fluorescent Probe Discovery
Source: J Org Chem. 2026 Apr 23;91(18):6448–59. doi: 10.1021/acs.joc.6c00381 (PMC13162310; doi:10.1021/acs.joc.6c00381)

Supporting Information for:

Palladium-Catalyzed C2 Functionalization of an Iodinated Tryptophan Scaffold for Fluorescent Probe Discovery

*Eoghan J. McArthur, Leung Yiu Wong, Valeria K. Burianova and Andrew Sutherland**

School of Chemistry, The Joseph Black Building, University of Glasgow,

Glasgow G12 8QQ, United Kingdom.

Table of Contents

1. Photophysical Data for α-Amino Acids **7a**–**d**, **8a**–**k**, **11** and Dipeptides **10** and **13** S2–S15

2. ^{1}H and ^{13}C NMR Spectra for all Compounds S16–S59

1. Photophysical Data for α-Amino Acids 7a–d, 8a–k, 11 and Dipeptides 10 and 13.

Table S1. Photophysical Data of All Amino Acids.

amino acid	λ_{Abs} (nm)[a]	ε (cm^{-1} M^{-1})	λ_{Em} (nm)[a]	Φ_F[b]	brightness (cm^{-1} M^{-1})
**7a**	320	30200	404	0.010	350
**7b**	324	40500	386	0.010	405
**7c**	320	32800	381	0.010	328
**7d**	356	32300	490	0.0030	110
**8a**	302	18600	369	0.44	8184
**8b**	304	10900	362	1.0	10900
**8c**	320	32300	386	0.83	26809
**8d**	338	18100	493	0.16	2896
**8e**	330	18000	441	0.73	13180
**8f**	300	18700	367	0.76	14212
**8g**	312	16000	405	0.76	12160
**8h**	318	24600	418	0.40	9940
**8i**	318	35300	417	1.0	35300
**8j**	330	28000	411	0.66	18480
**8k**	330	26800	393	0.83	22244
**11**	324	11800	385	0.42	4900

[a]Spectra were recorded at concentrations of 3.5–10 μM in acetonitrile. [b]Quantum yields (Φ_F) were determined in acetonitrile using anthracene and L-tryptophan as standards.

Figure S1: Absorption and Emission Spectra for **7a** (10 μM). Excitation at 328 nm.

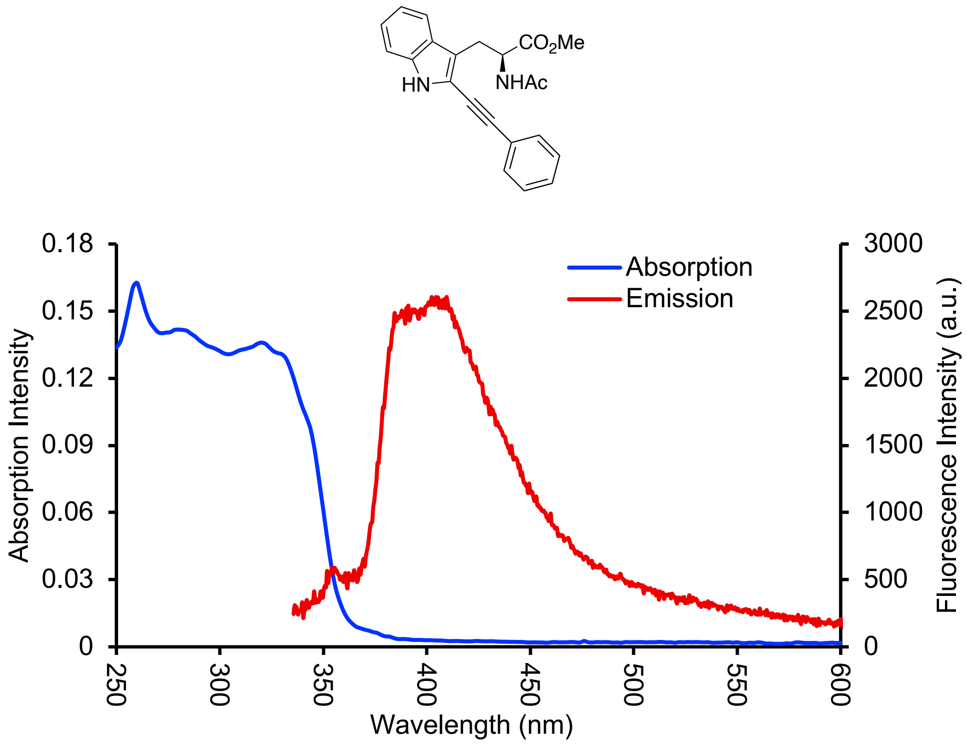

Figure S2: Absorption and Emission Spectra for **7b** (5 μM). Excitation at 340 nm.

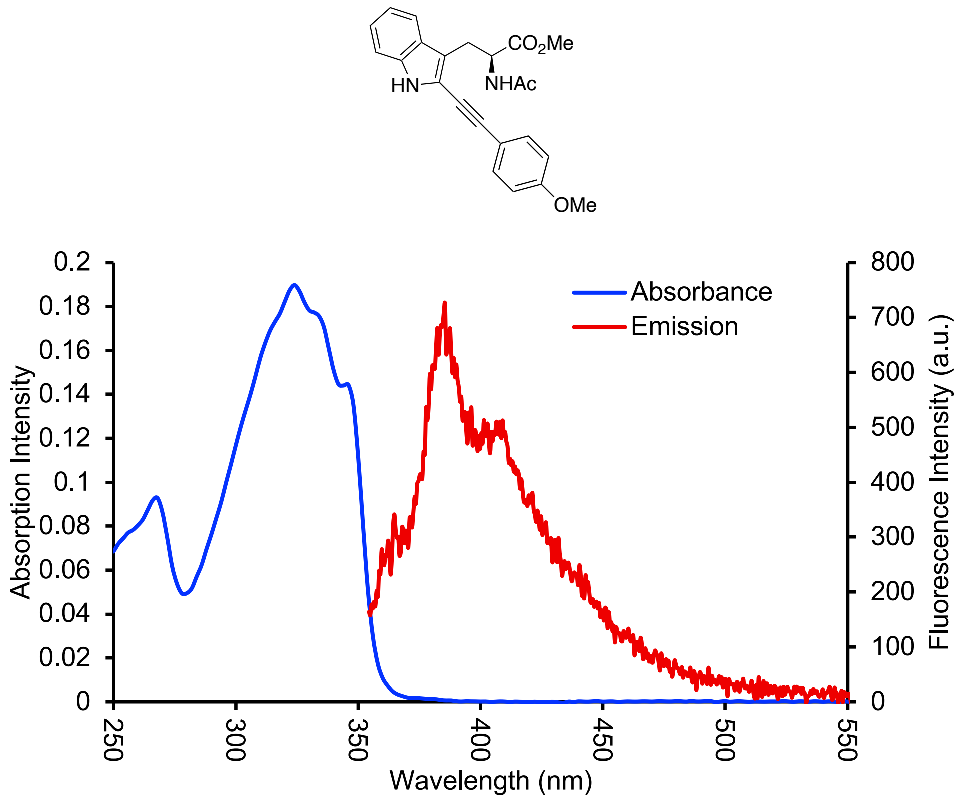

Figure S3: Absorption and Emission Spectra for **7c** (5 μM). Excitation at 340 nm.

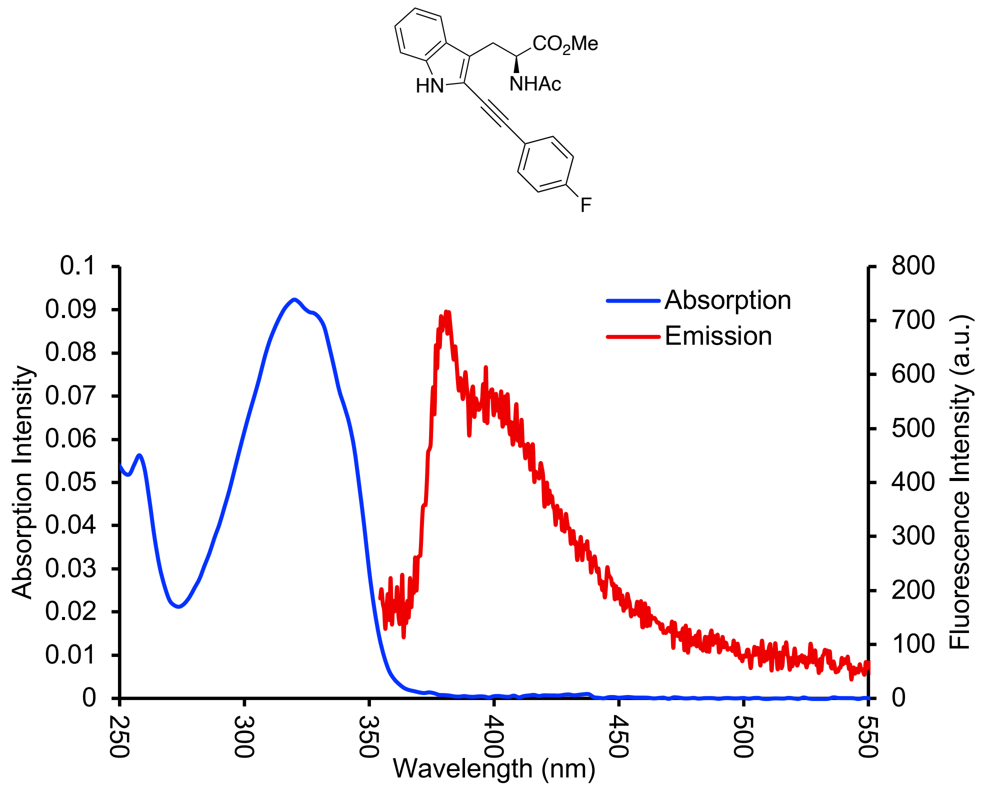

Figure S4: Absorption and Emission Spectra for **7d** (3.5 μM). Excitation at 328 nm.

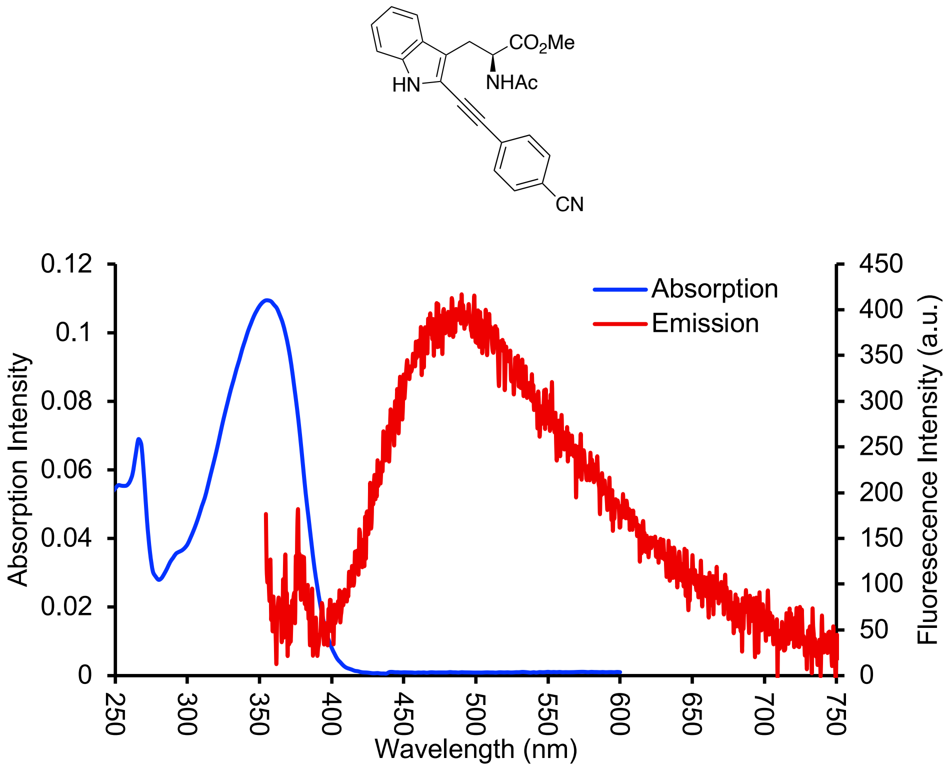

Figure S5: Absorption and Emission Spectra for **8a** (5 μM). Excitation at 310 nm.

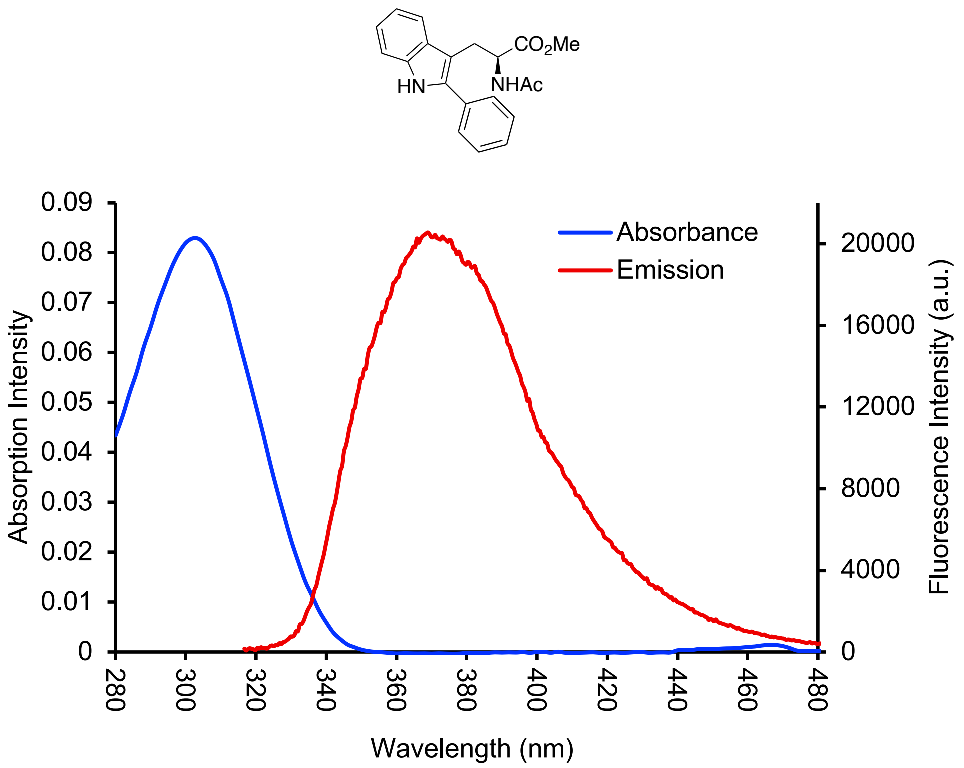

Figure S6: Absorption and Emission Spectra for **8b** (5 μM). Excitation at 316 nm.

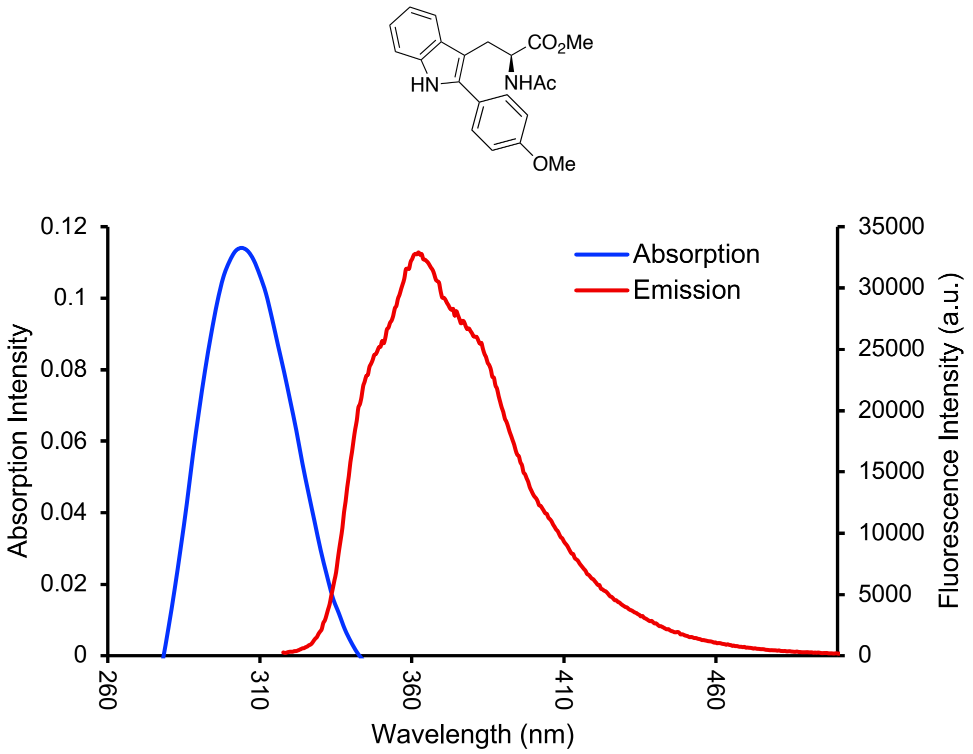

Figure S7: Absorption and Emission Spectra for **8c** (5 μM). Excitation at 316 nm.

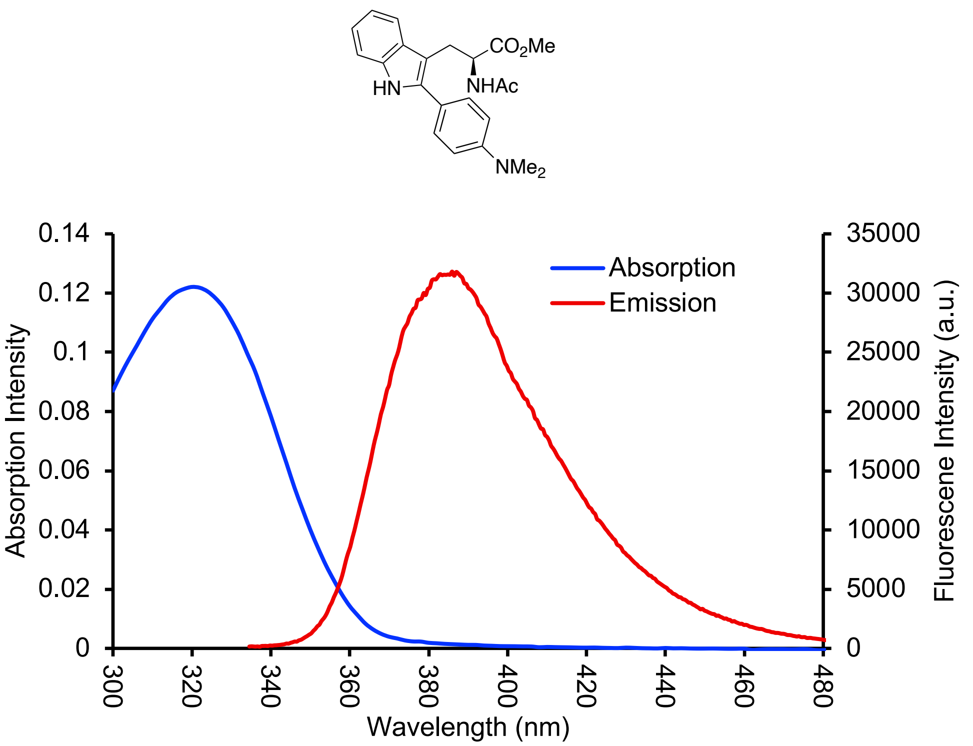

Figure S8: Absorption and Emission Spectra for **8d** (5 μM). Excitation at 338 nm.

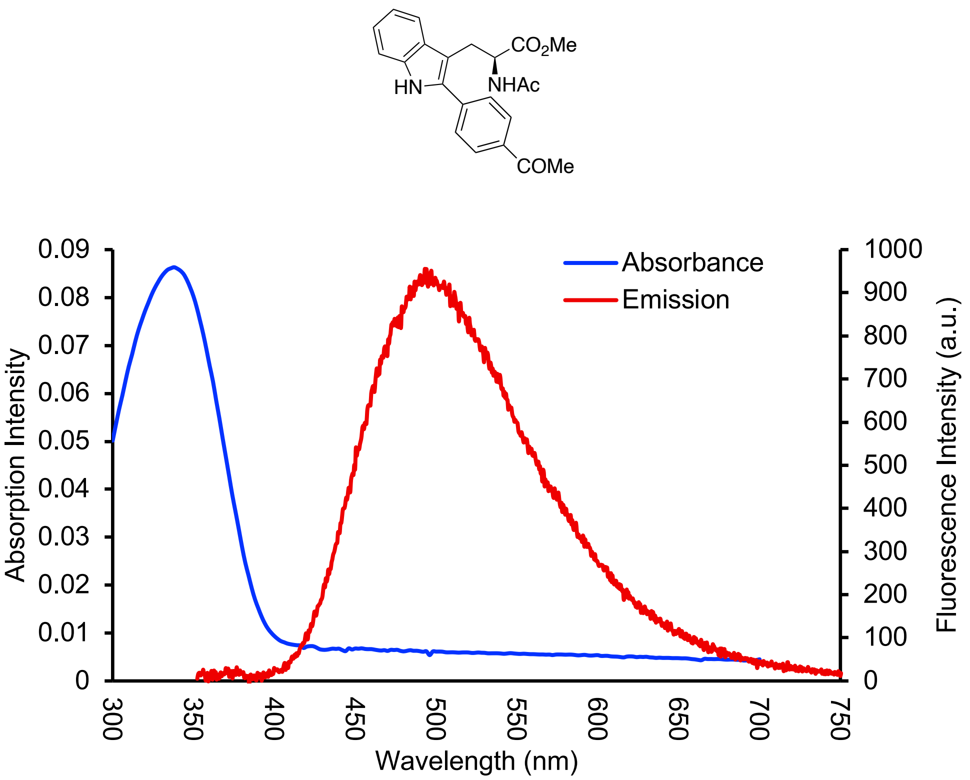

Figure S9: Absorption and Emission Spectra for **8e** (5 μM). Excitation at 338 nm.

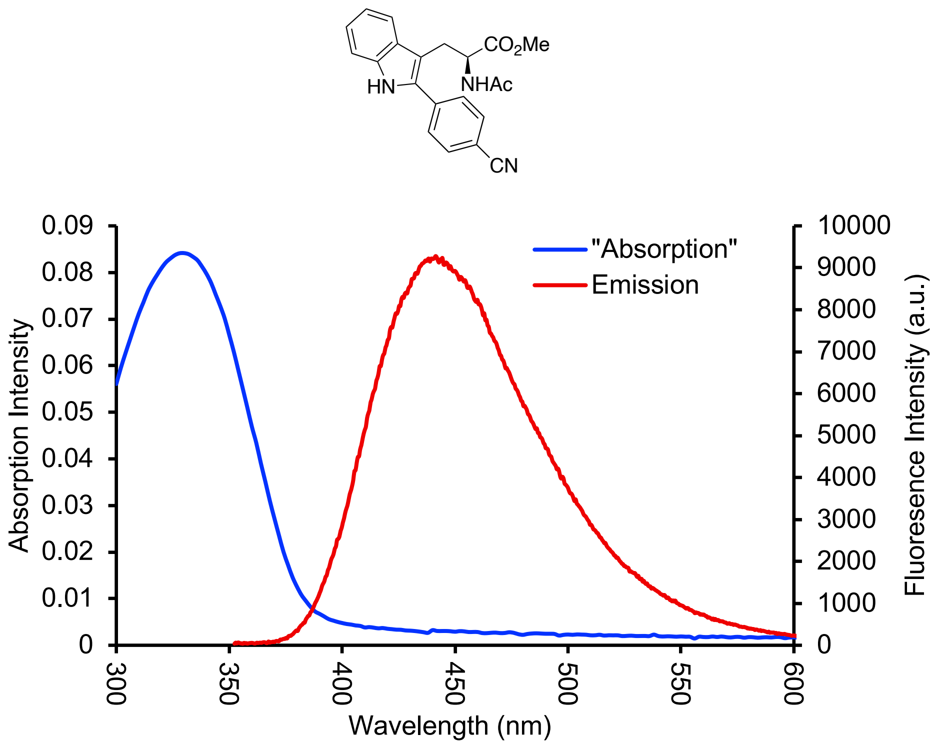

Figure S10: Absorption and Emission Spectra for **8f** (5 μM). Excitation at 310 nm.

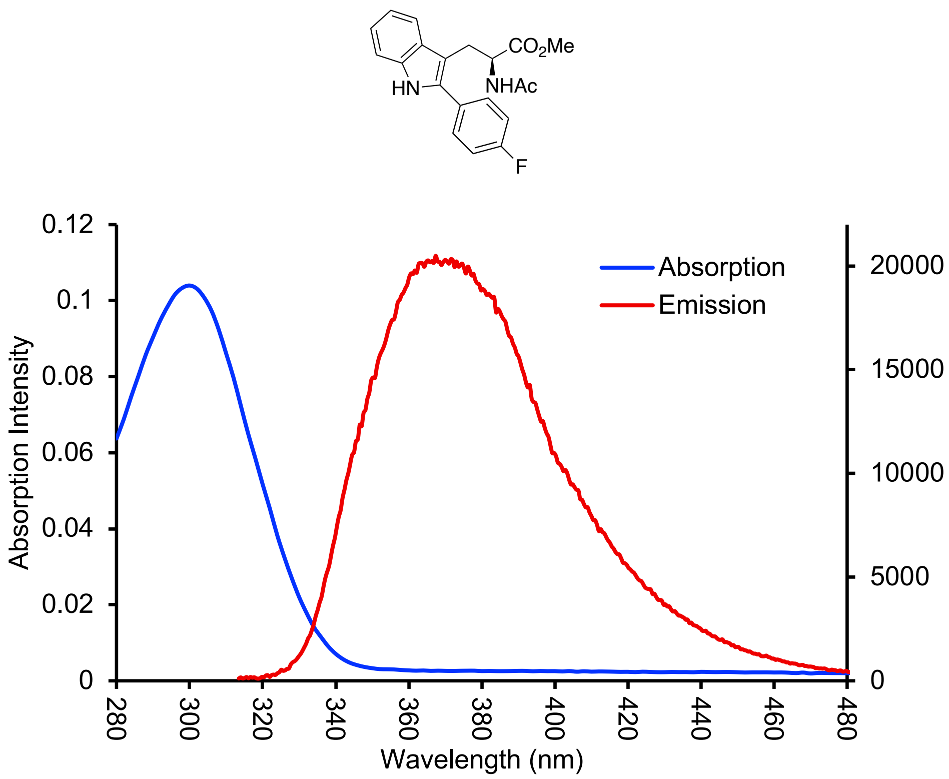

Figure S11: Absorption and Emission Spectra for **8g** (5 μM). Excitation at 310 nm.

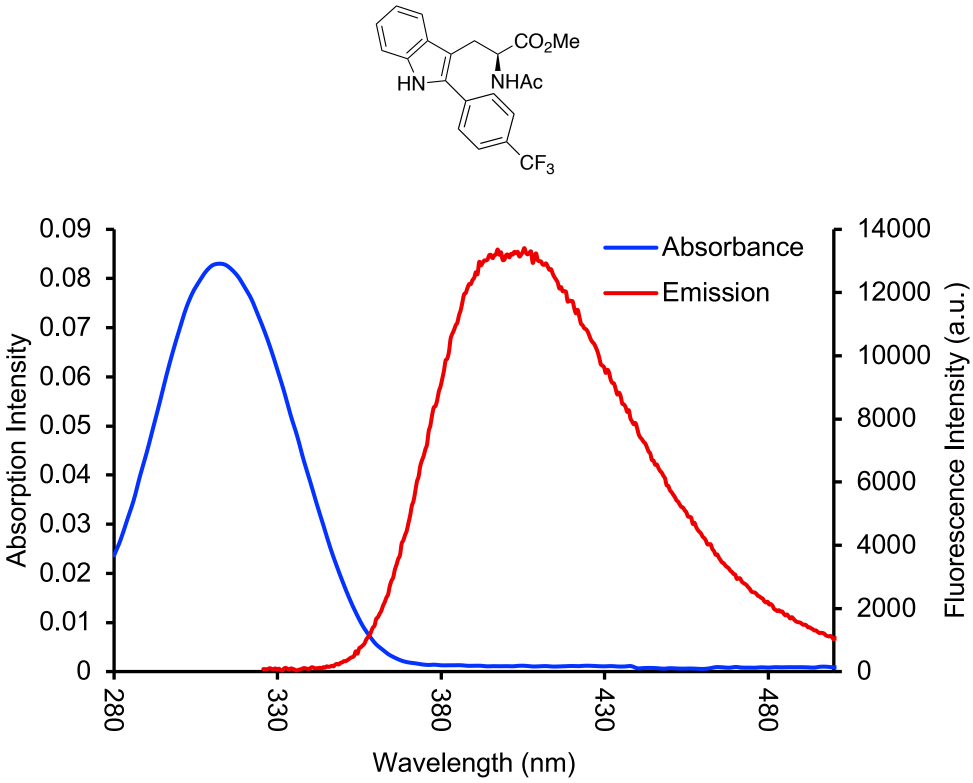

Figure S12: Absorption and Emission Spectra for **8h** (5 μM). Excitation at 316 nm.

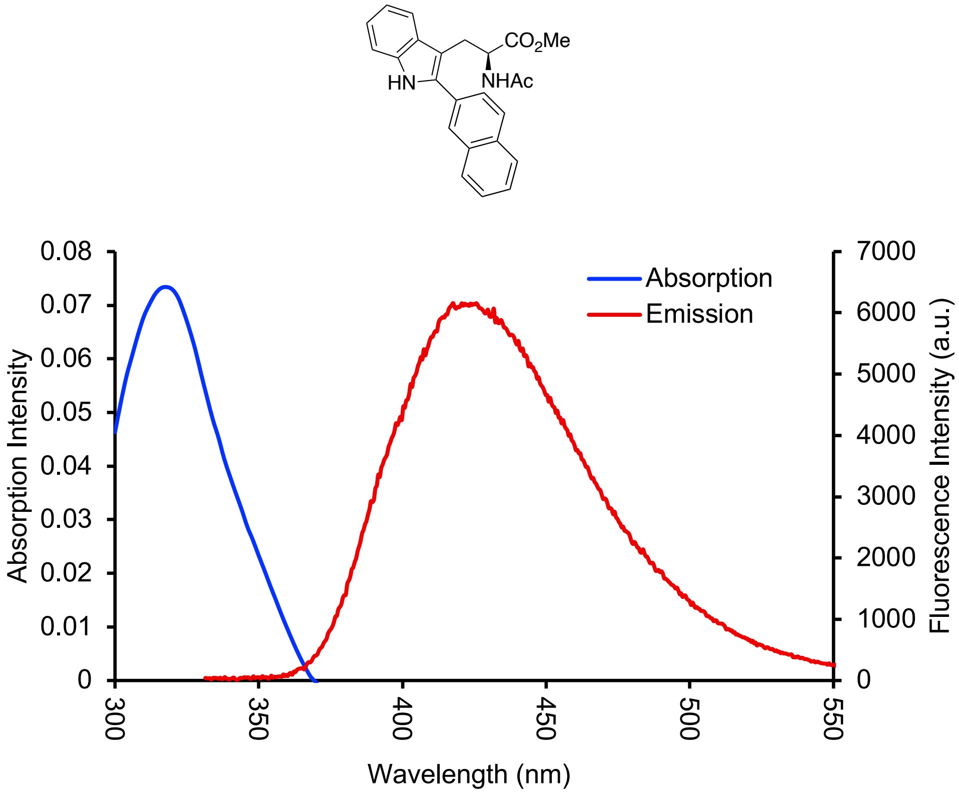

Figure S13: Absorption and Emission Spectra for **8i** (5 μM). Excitation at 320 nm.

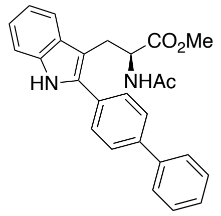

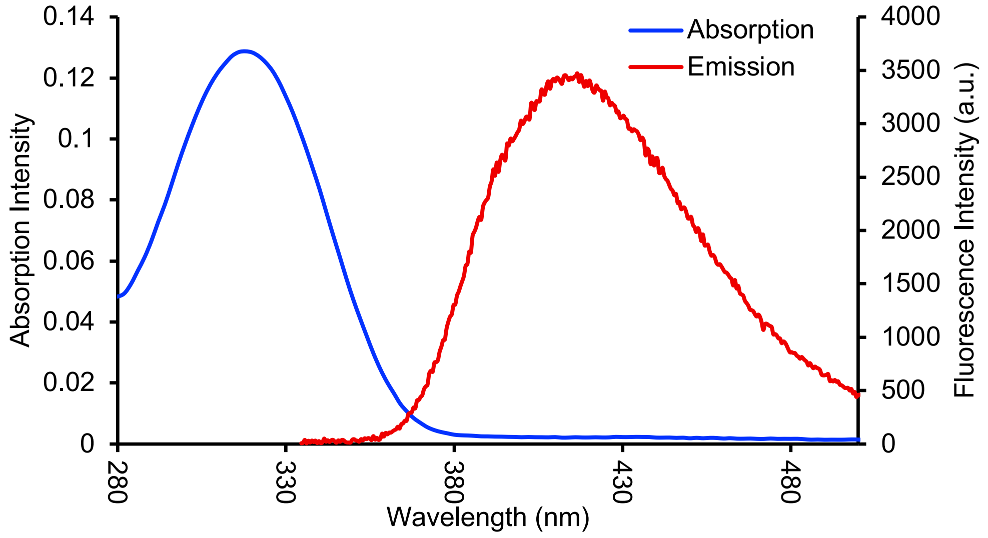

Figure S14: Absorption and Emission Spectra for **8j** (5 μM). Excitation at 330 nm.

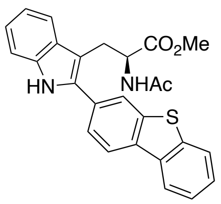

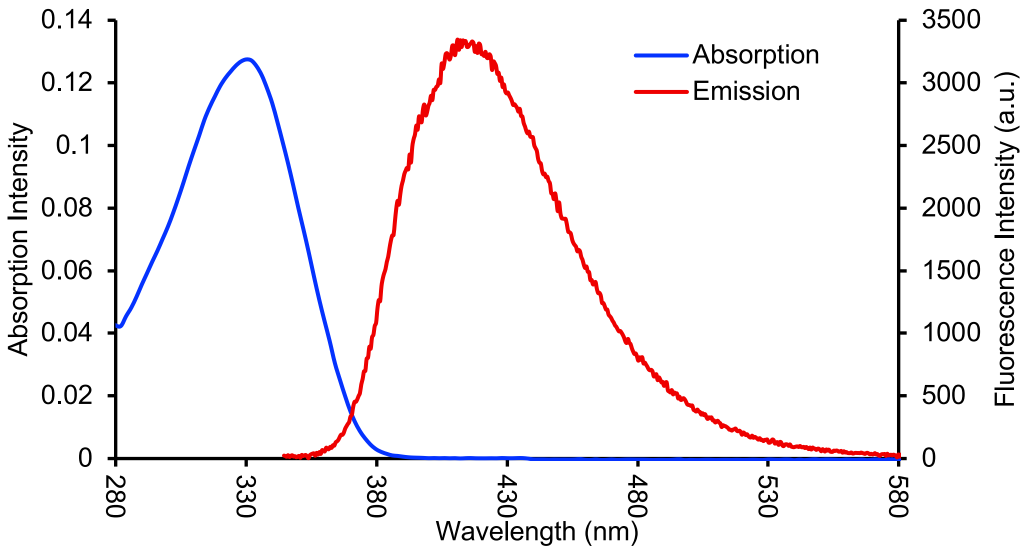

Figure S15: Absorption and Emission Spectra for **8k** (5 μM). Excitation at 330 nm.

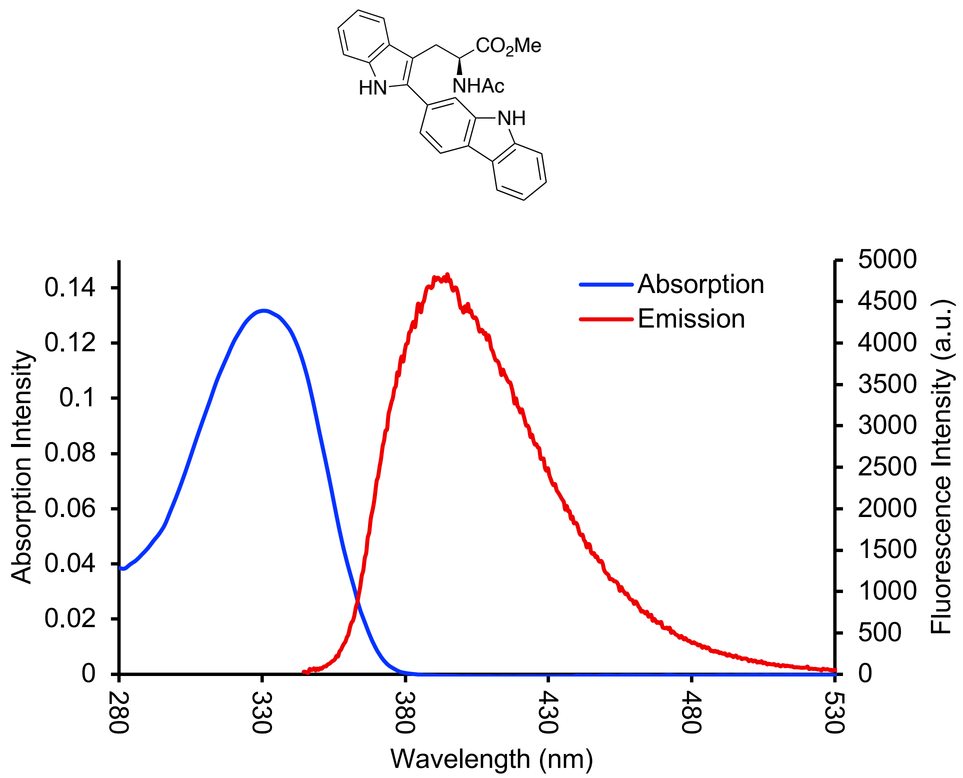

Figure S16: Absorption and Emission Spectra for **10** (5 μM). Excitation at 319 nm.

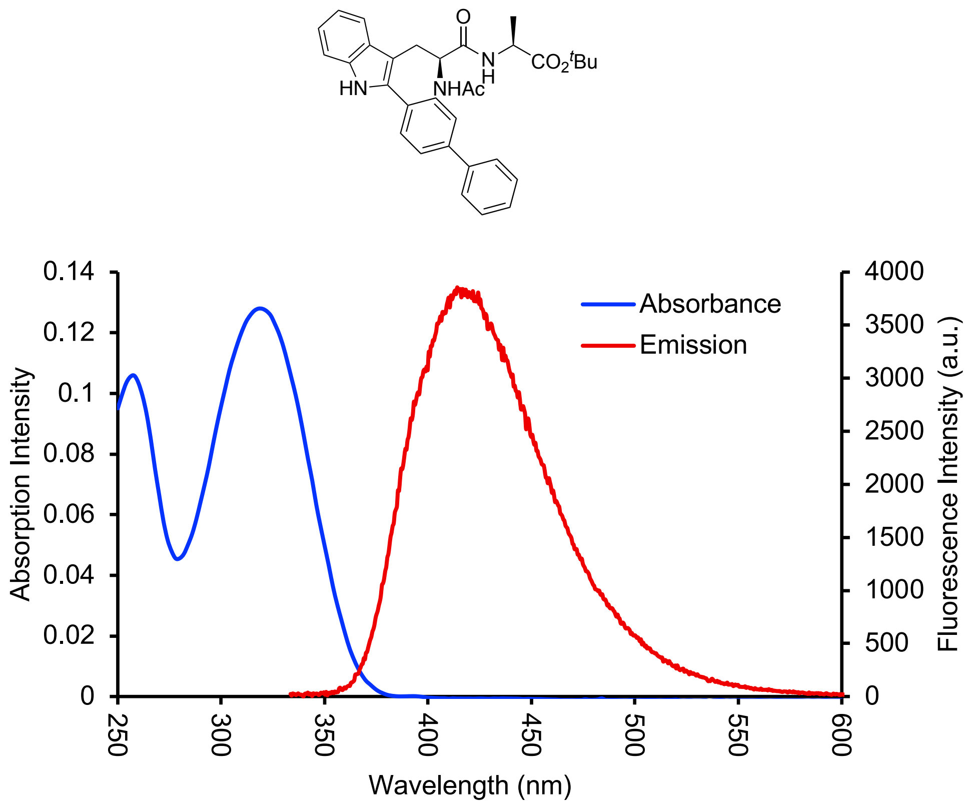

Figure S17: Absorption and Emission Spectra for **13** (5 μM). Excitation at 280 and 320 nm.

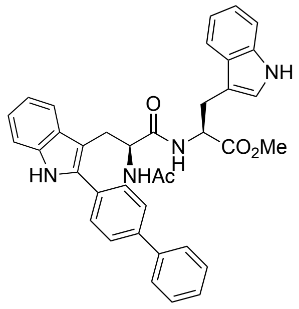

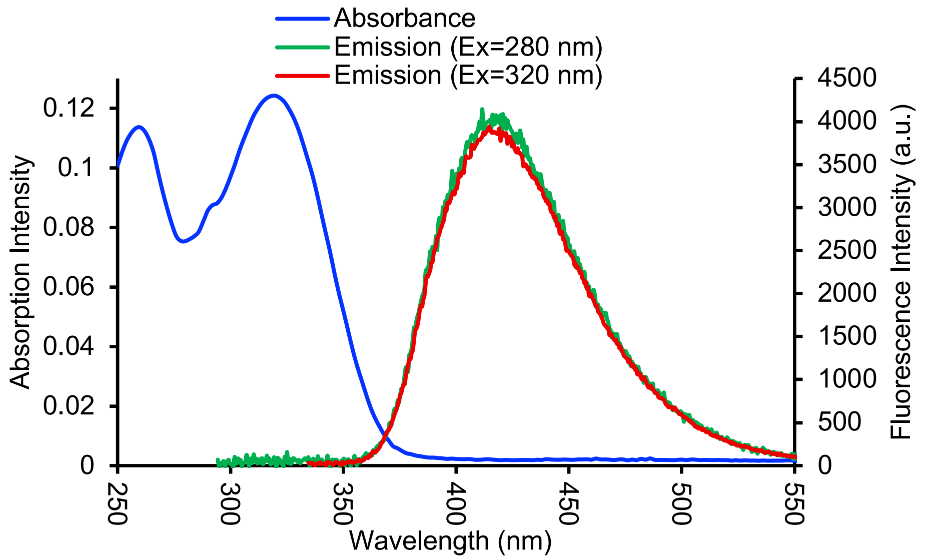

Figure S18: Additional Photophysical Data for **8i**.

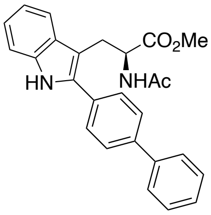

Solvatochromic Study (5 μM):

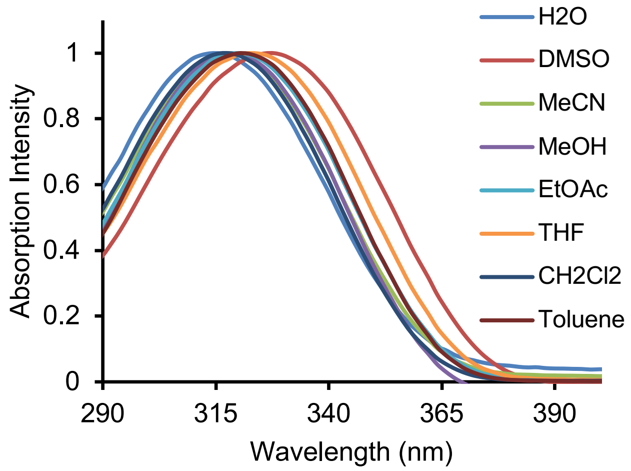

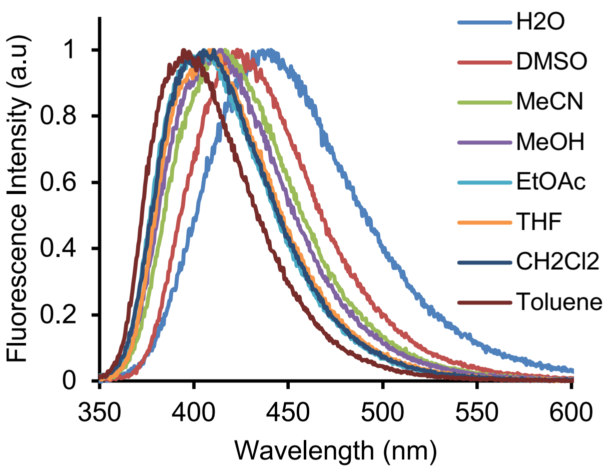

Emission spectra showing relative intensities:

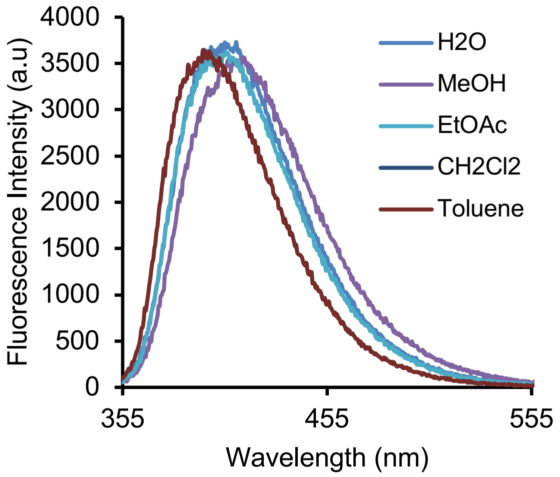

Lippert-Mataga Plot:

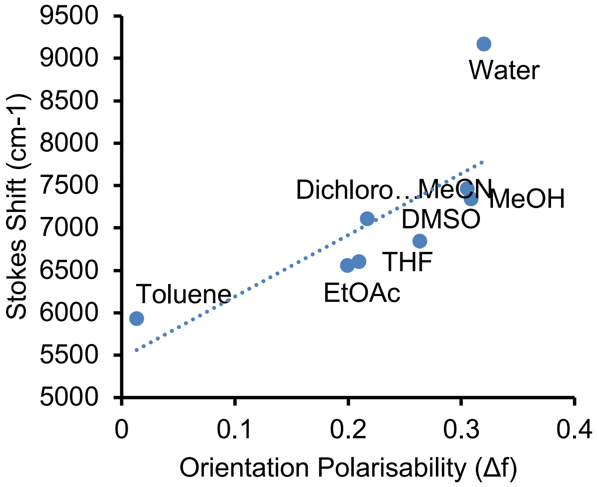

Viscosity study using biphenyl **8i**:

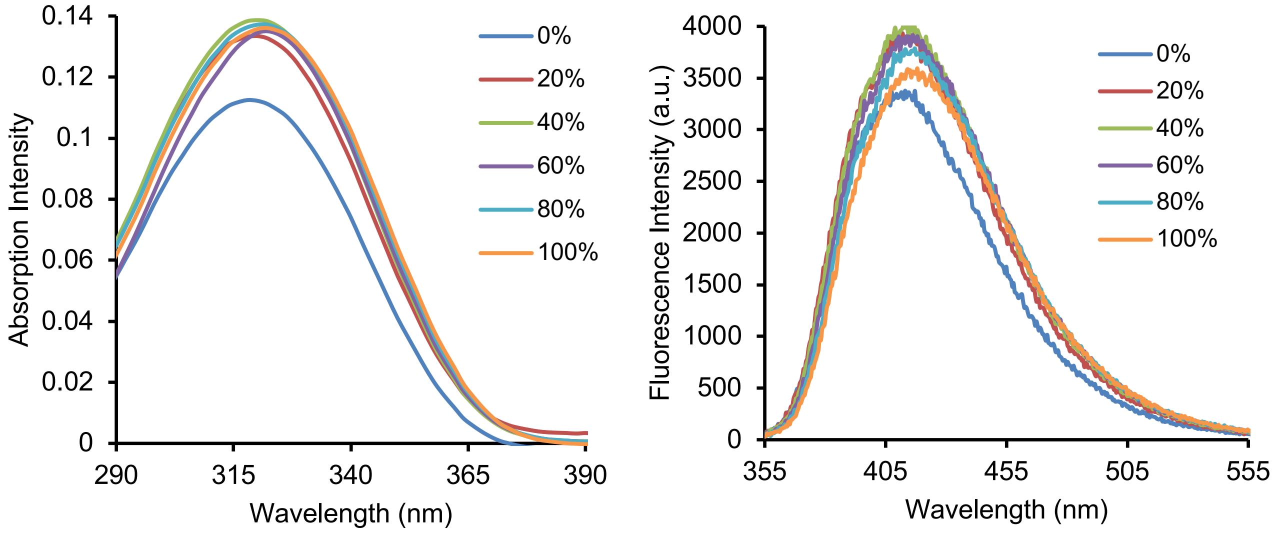

Aggregation study using biphenyl **8i** (absorption data at concentrations of 250 and 500 µM not shown due to detector saturation):

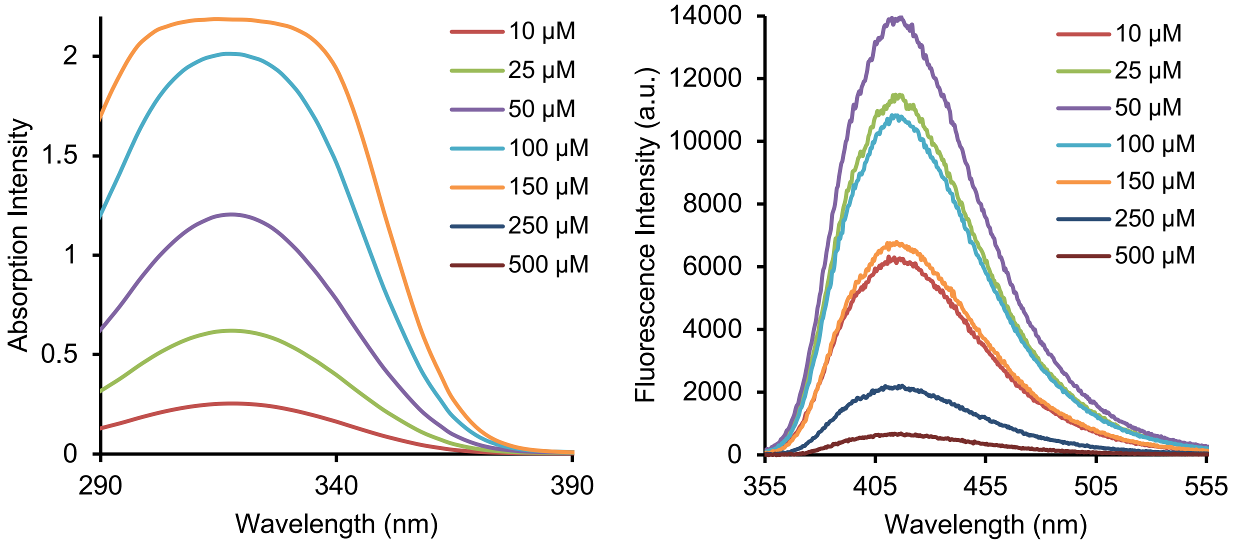

Figure S19: Additional Photophysical Data for **8k**.

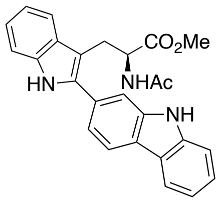

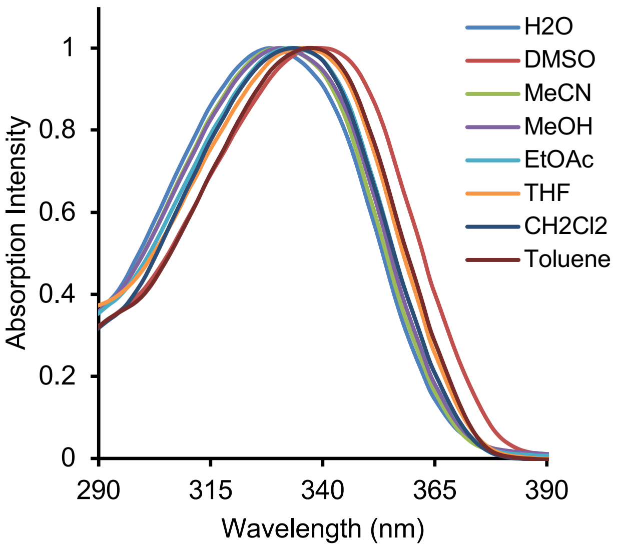

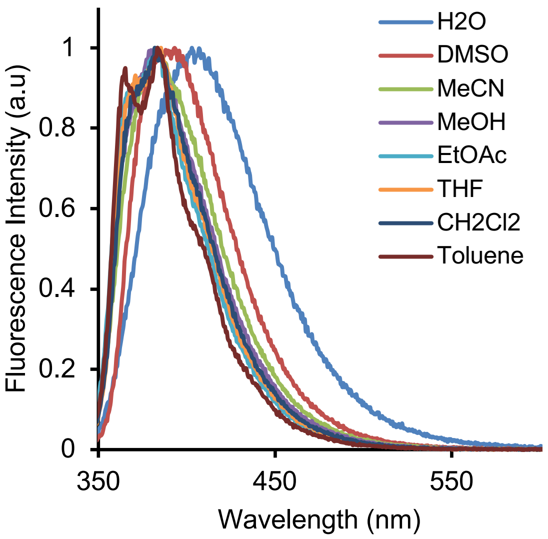

Emission spectra showing relative intensities:

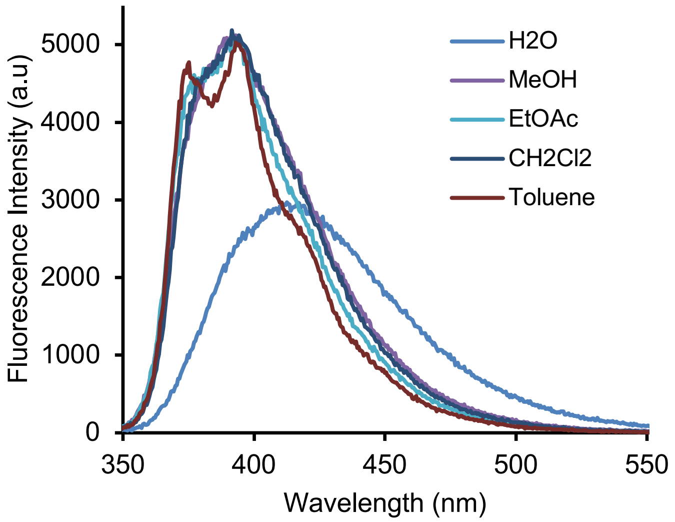

Figure S20: Photophysical Data for **11**.

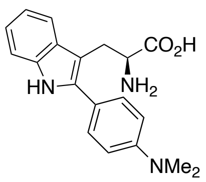

Absorption and Emission Spectra for **11** (5 µM in MeCN). Excitation at 310 nm.

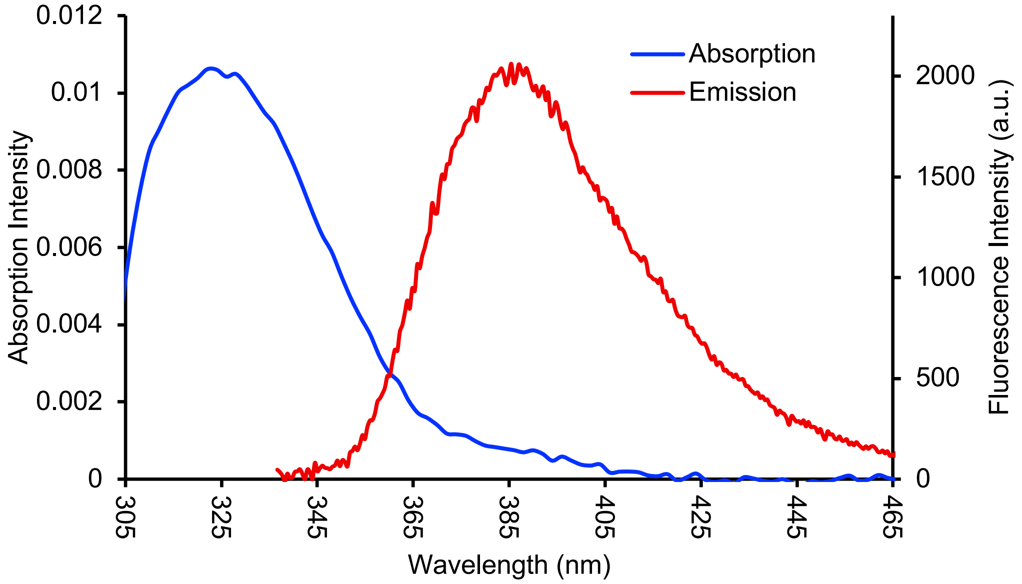

pH Study (5 µM in MeOH):

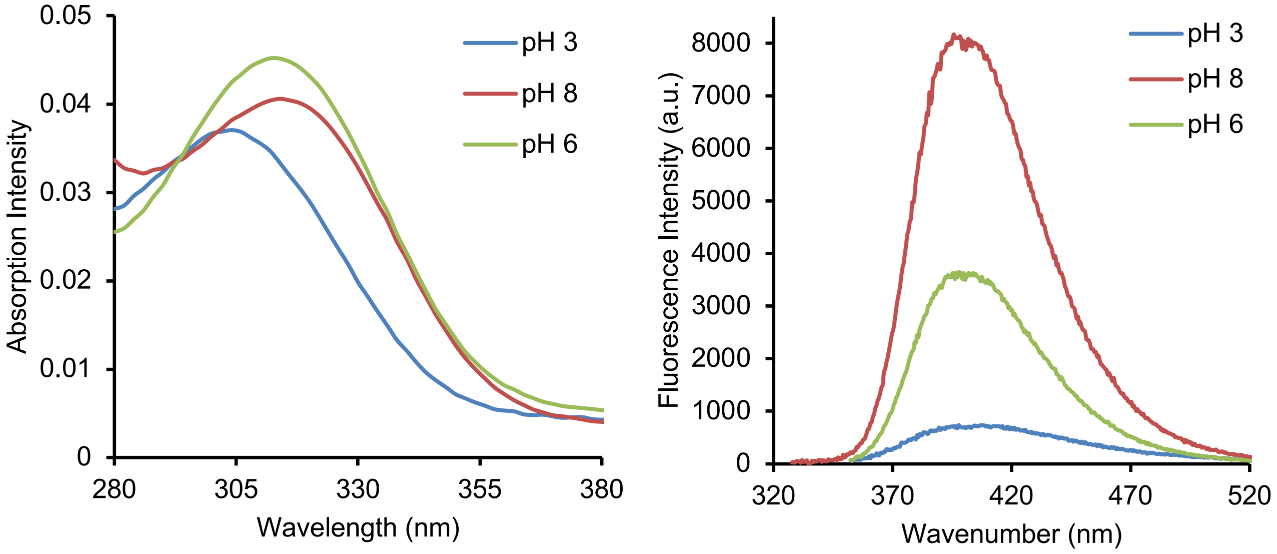

2. ^{1}H and ^{13}C NMR Spectra for all Compounds

^{1}H NMR (400 MHz, $CDCl_3$)

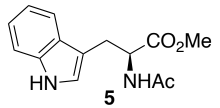

5

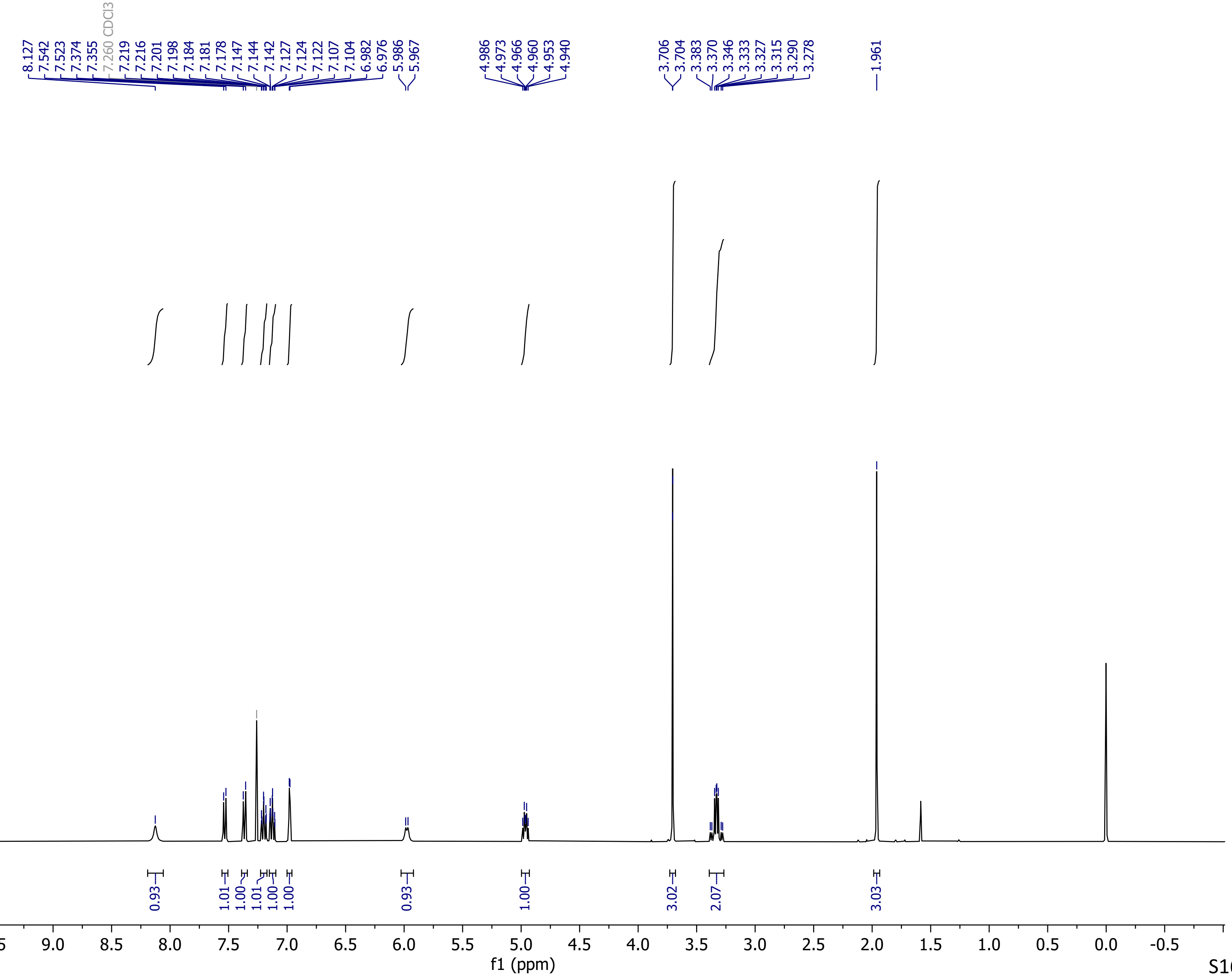

$^{13}C\{^{1}H\}$ NMR (101 MHz, $CDCl_3$)

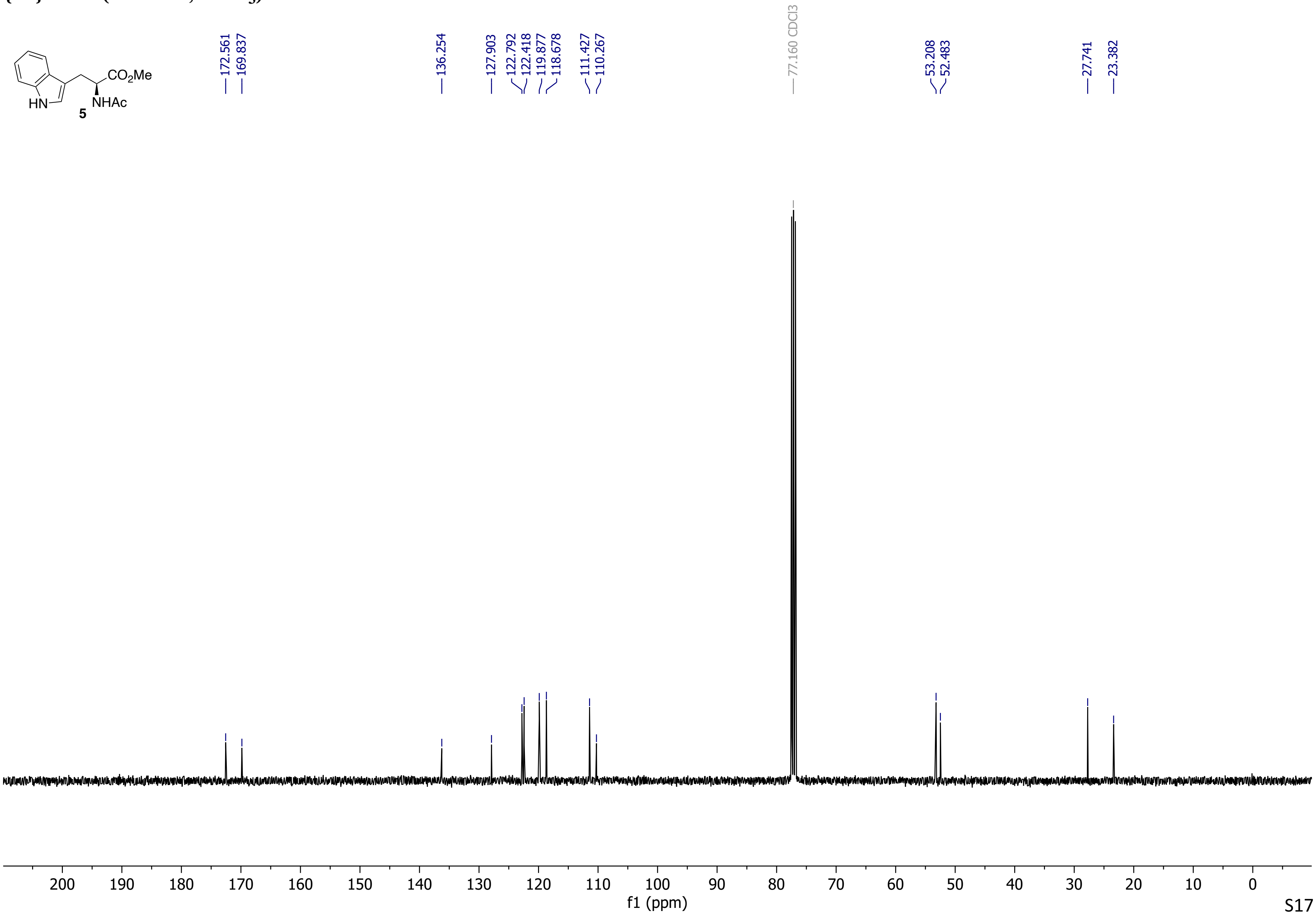

^{1}H NMR (400 MHz, $CDCl_3$)

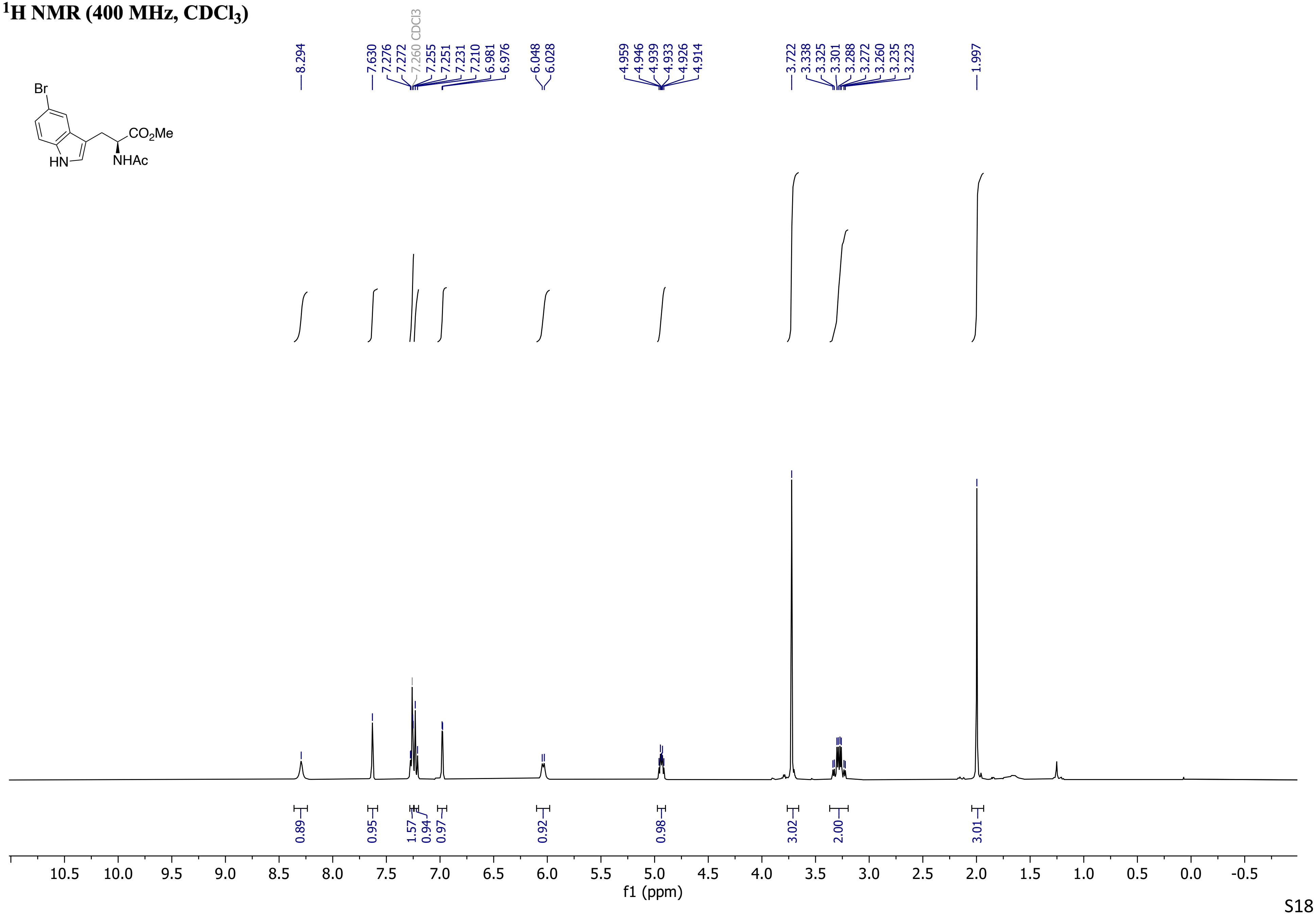

$^{13}C\{^{1}H\}$ NMR (101 MHz, $CDCl_3$)

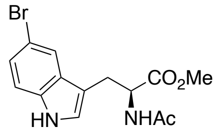

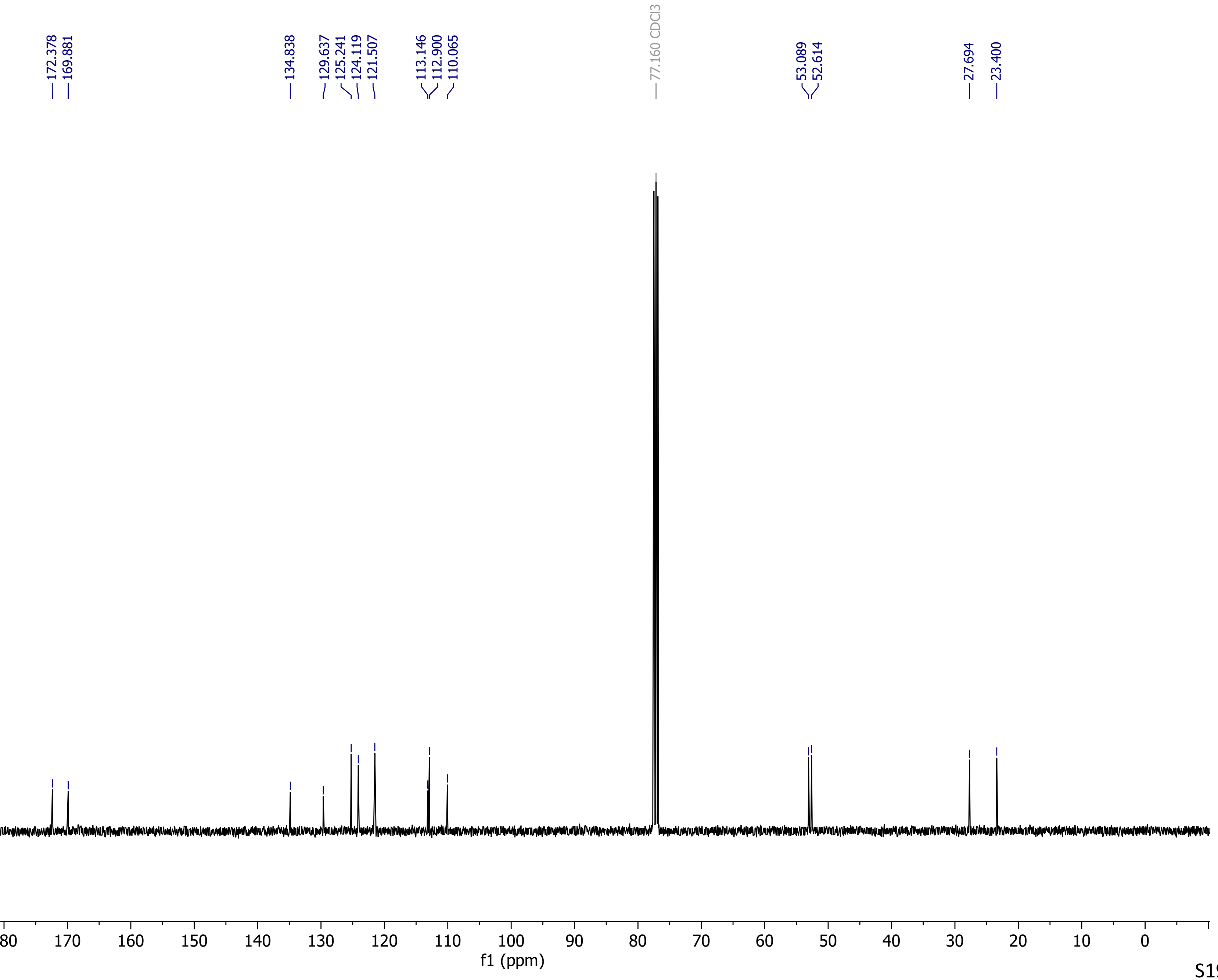

^{1}H NMR (400 MHz, $CDCl_3$)

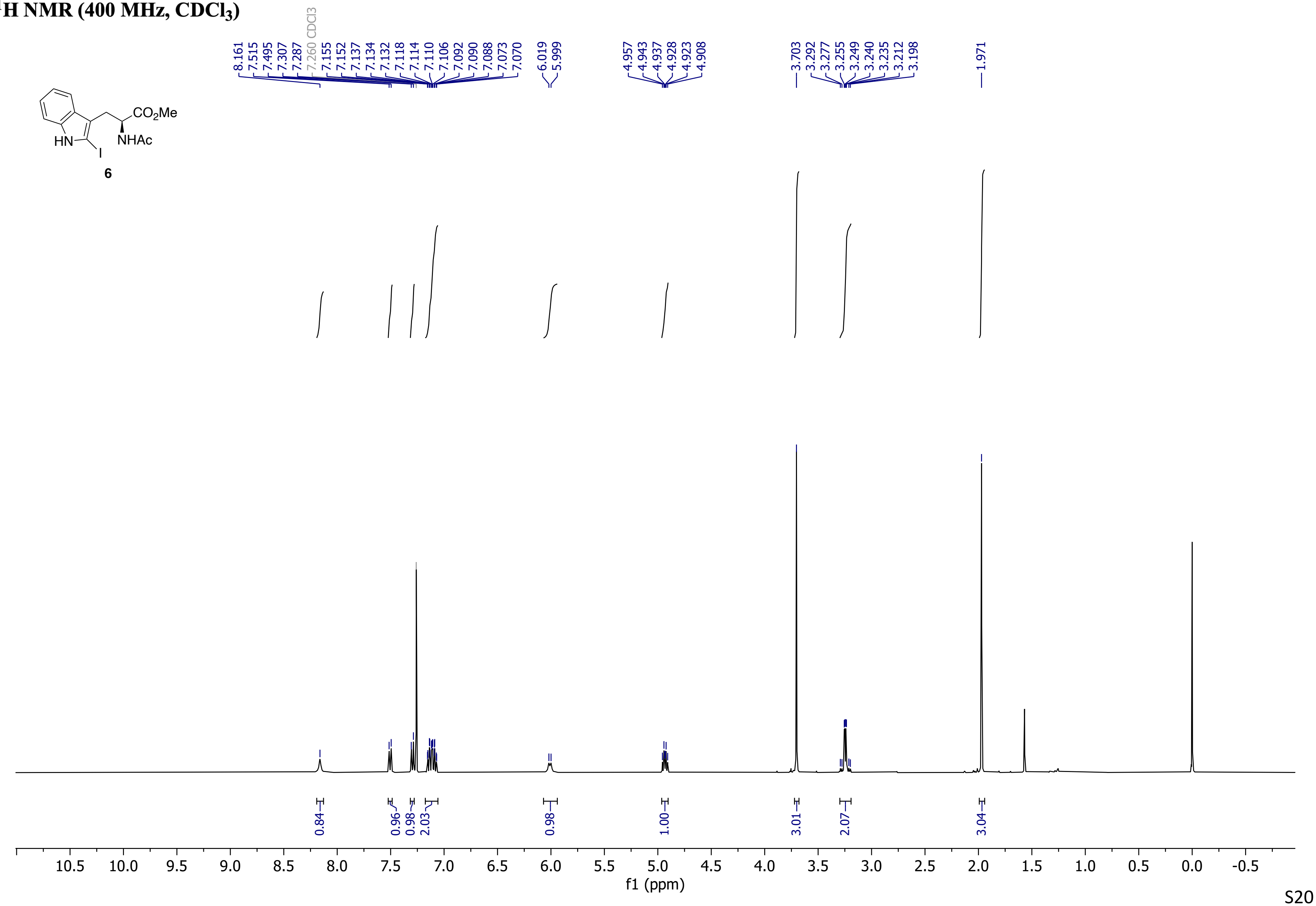

$^{13}C\{^1H\}$ NMR (101 MHz, $CDCl_3$)

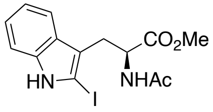

6

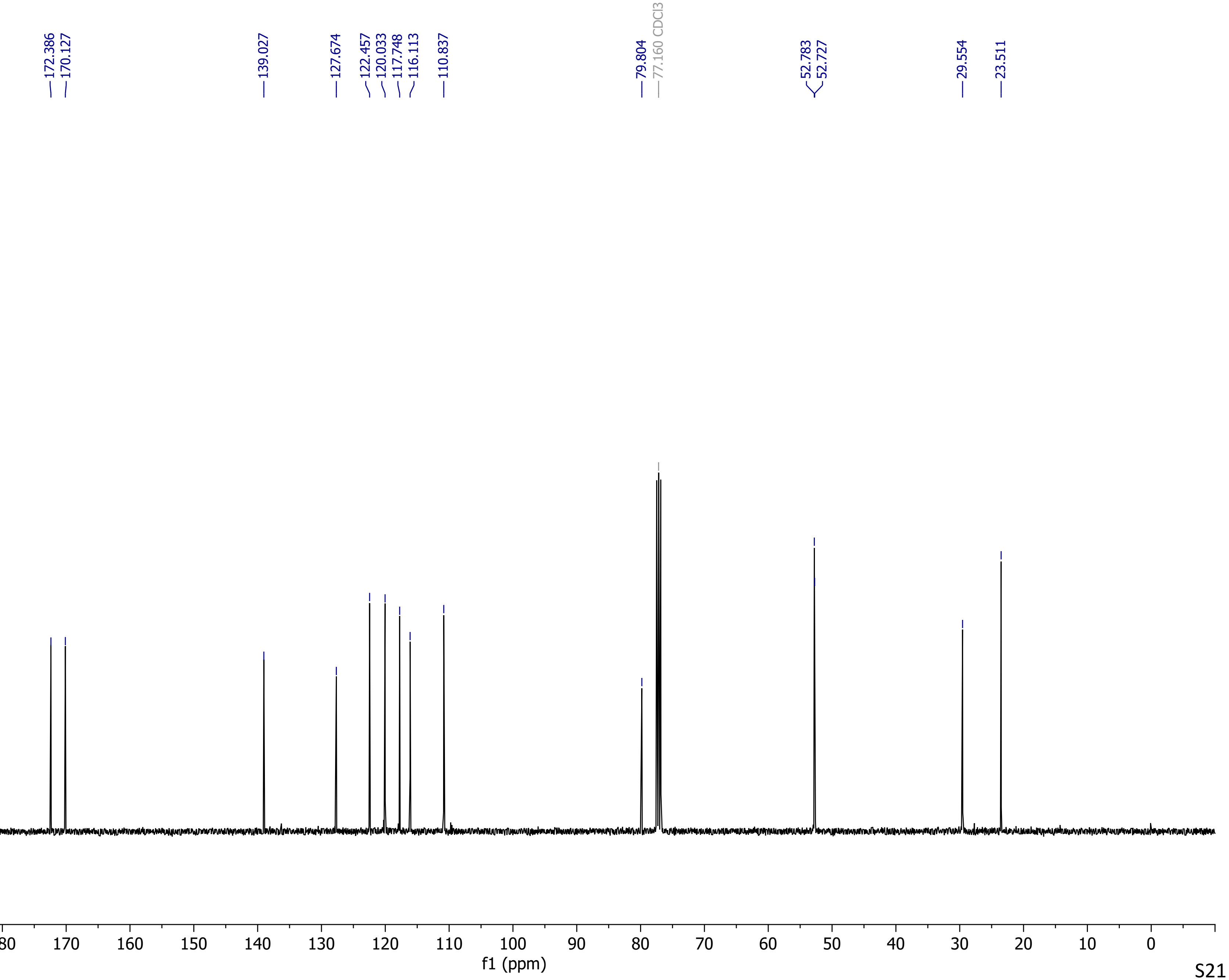

^{1}H NMR (400 MHz, $CDCl_3$)

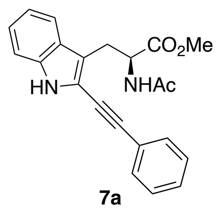

7a

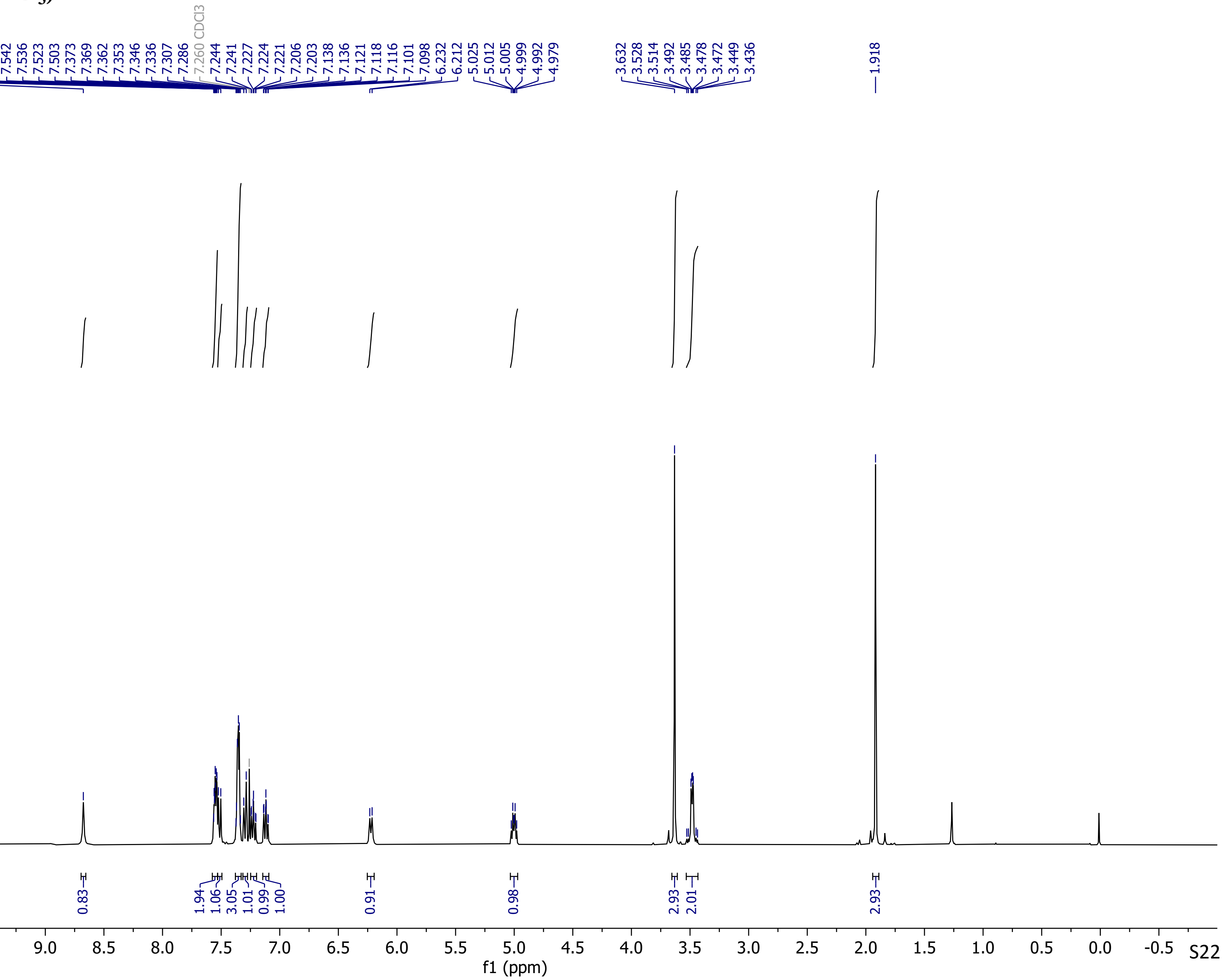

$^{13}C\{^1H\}$ NMR (101 MHz, $CDCl_3$)

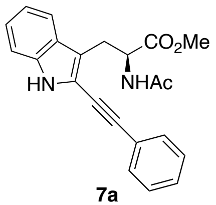

7a

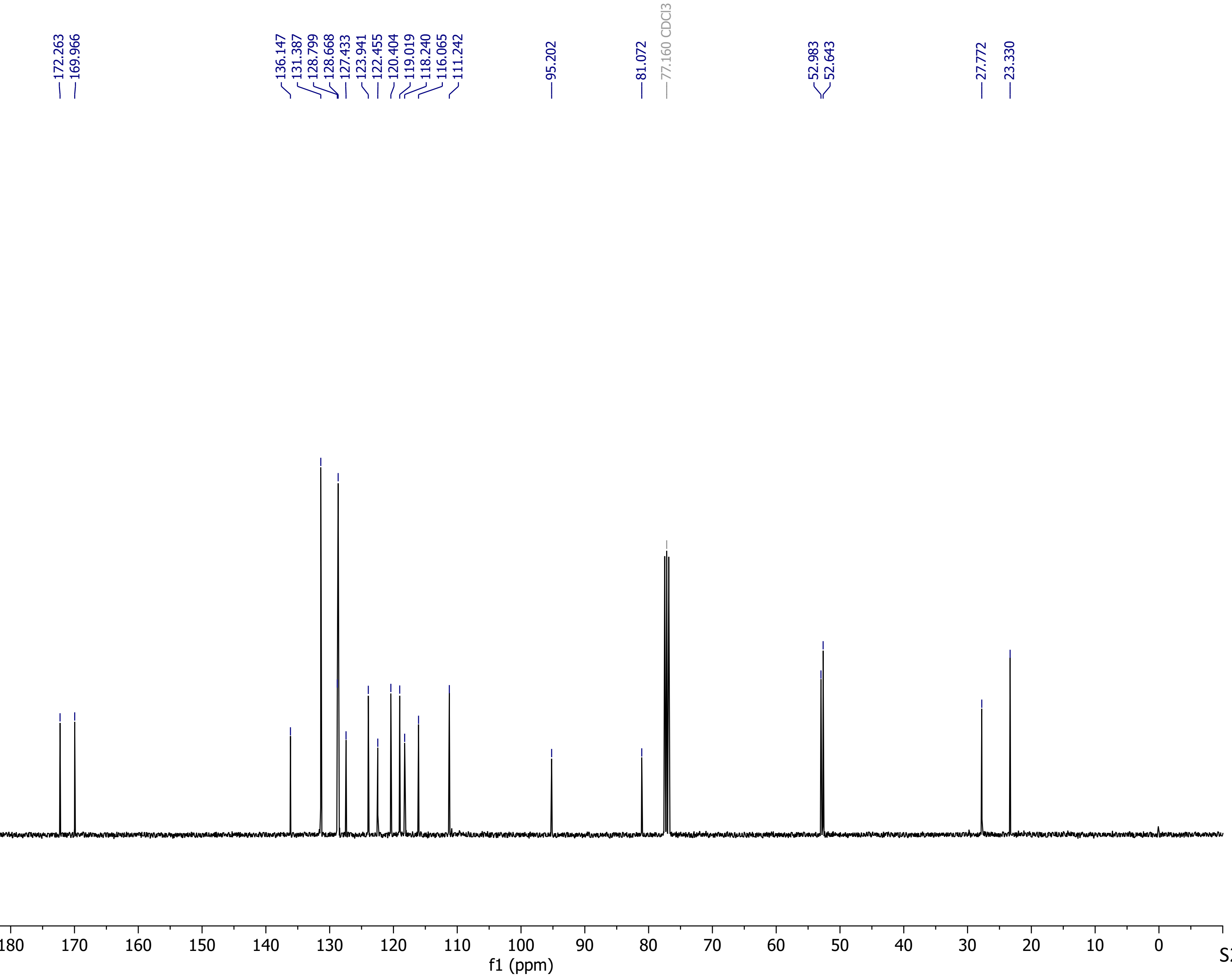

^{1}H NMR (400 MHz, $CDCl_3$)

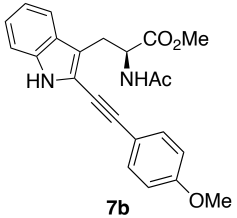

7b

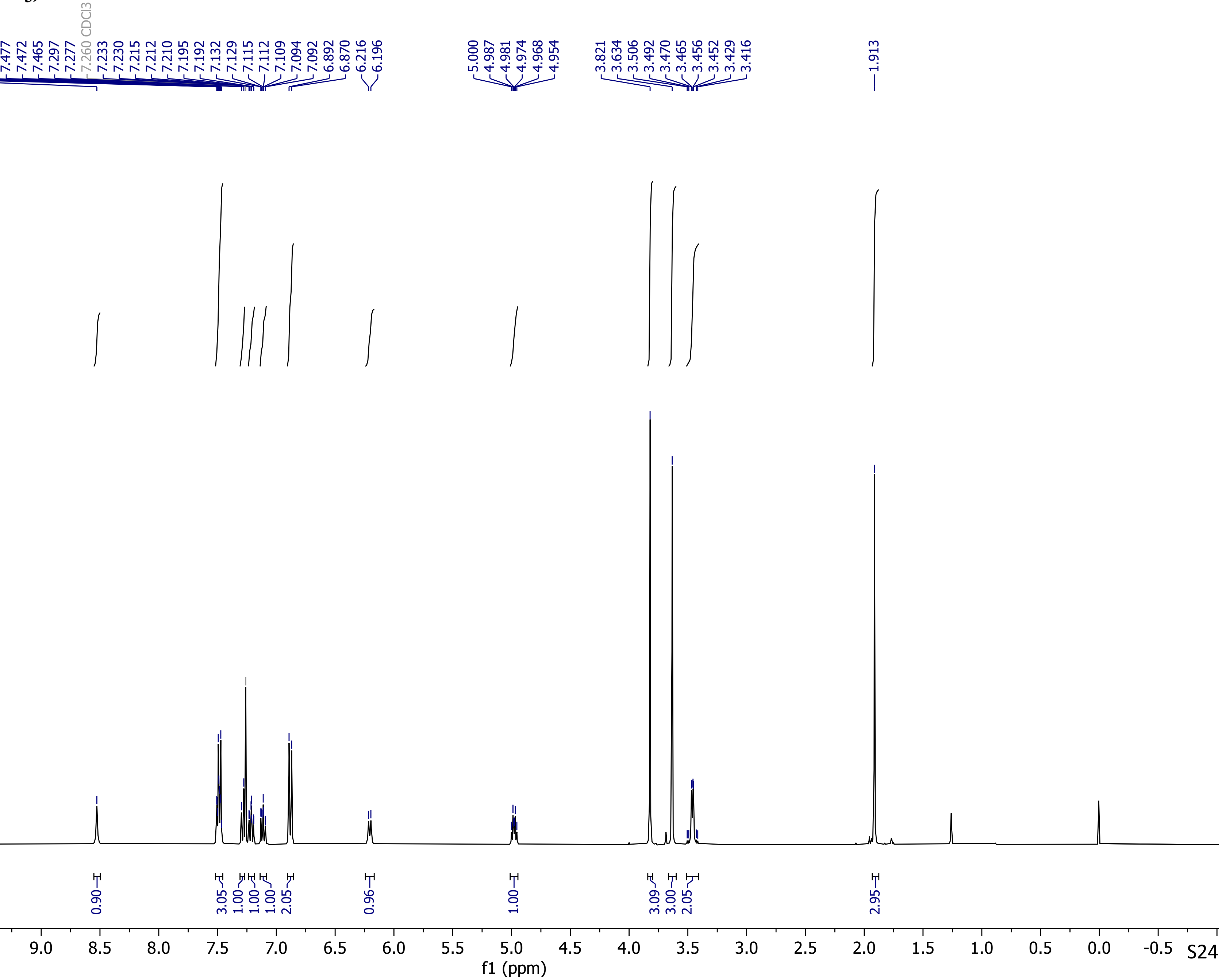

$^{13}C\{^{1}H\}$ NMR (101 MHz, $CDCl_3$)

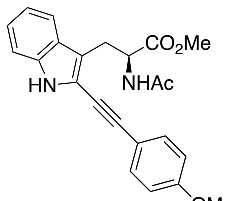

7b

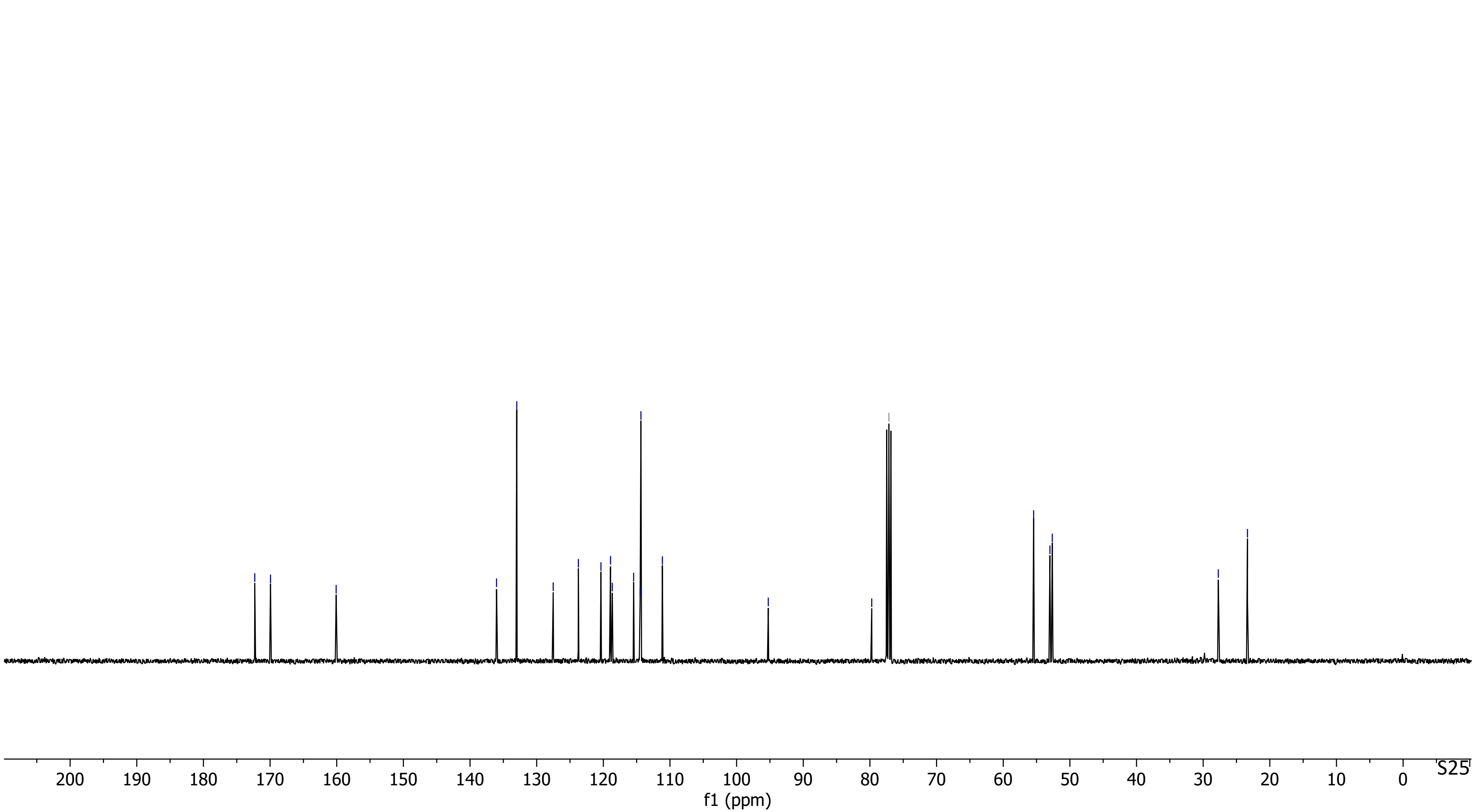

^{1}H NMR (400 MHz, CDCl$_3$)

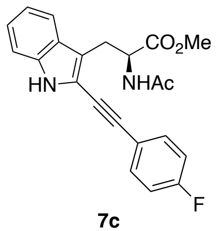

7c

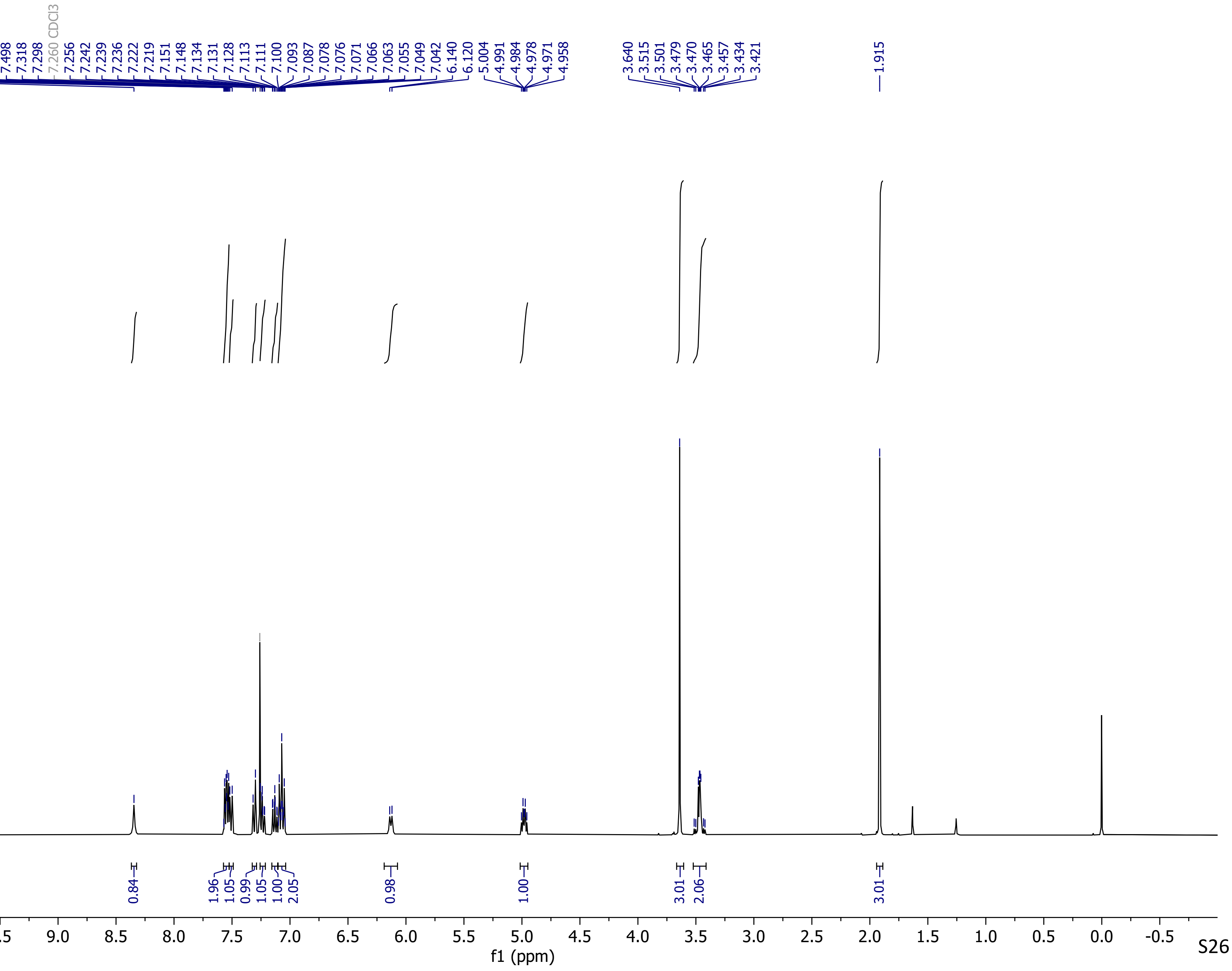

$^{13}C\{^{1}H\}$ NMR (101 MHz, $CDCl_3$)

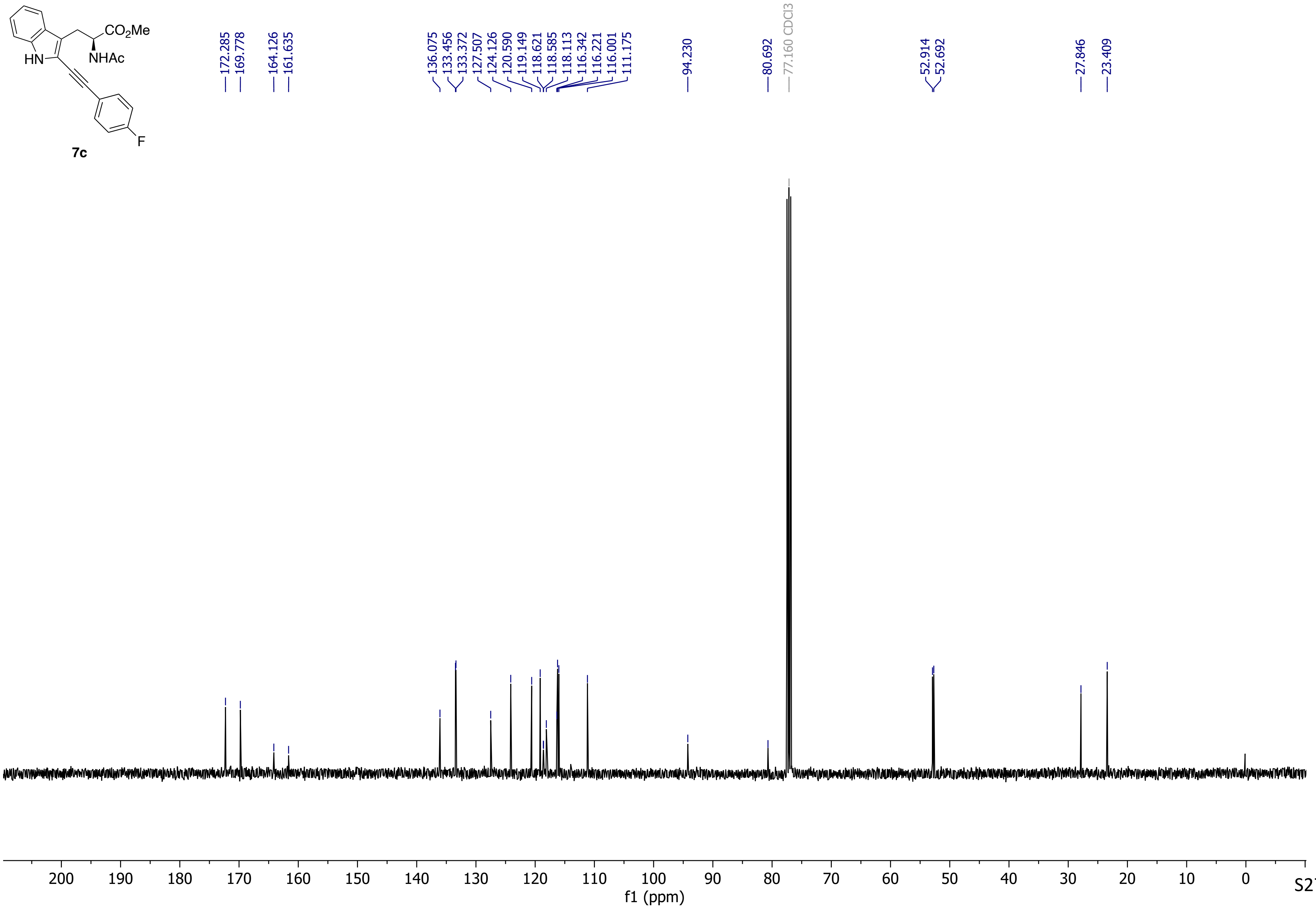

7c

^{1}H NMR (400 MHz, $CDCl_3$)

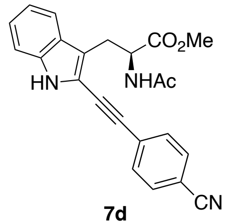

7d

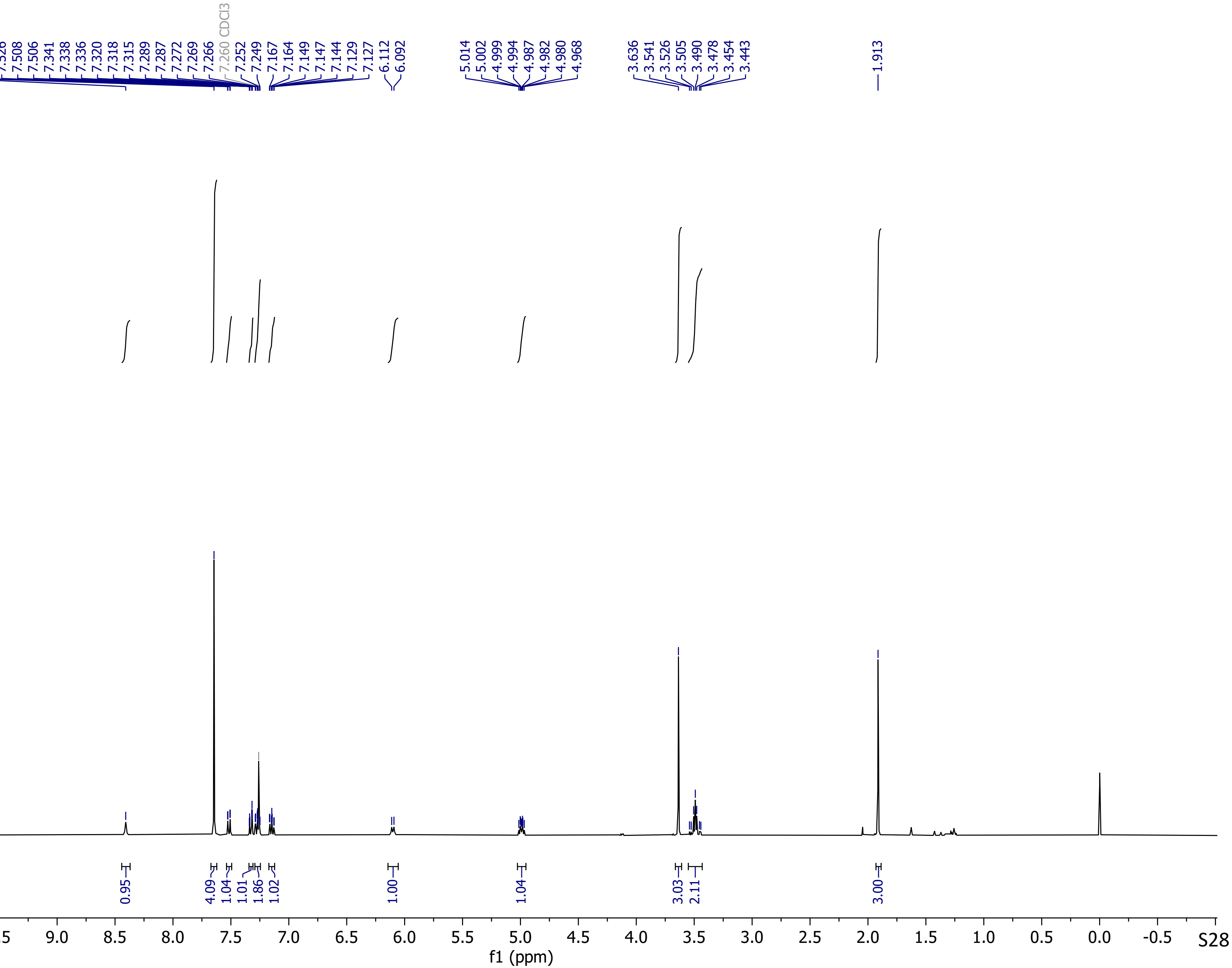

$^{13}C\{^{1}H\}$ NMR (101 MHz, $CDCl_3$)

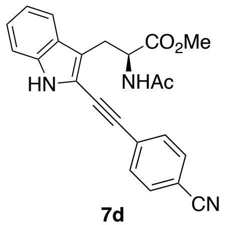

7d

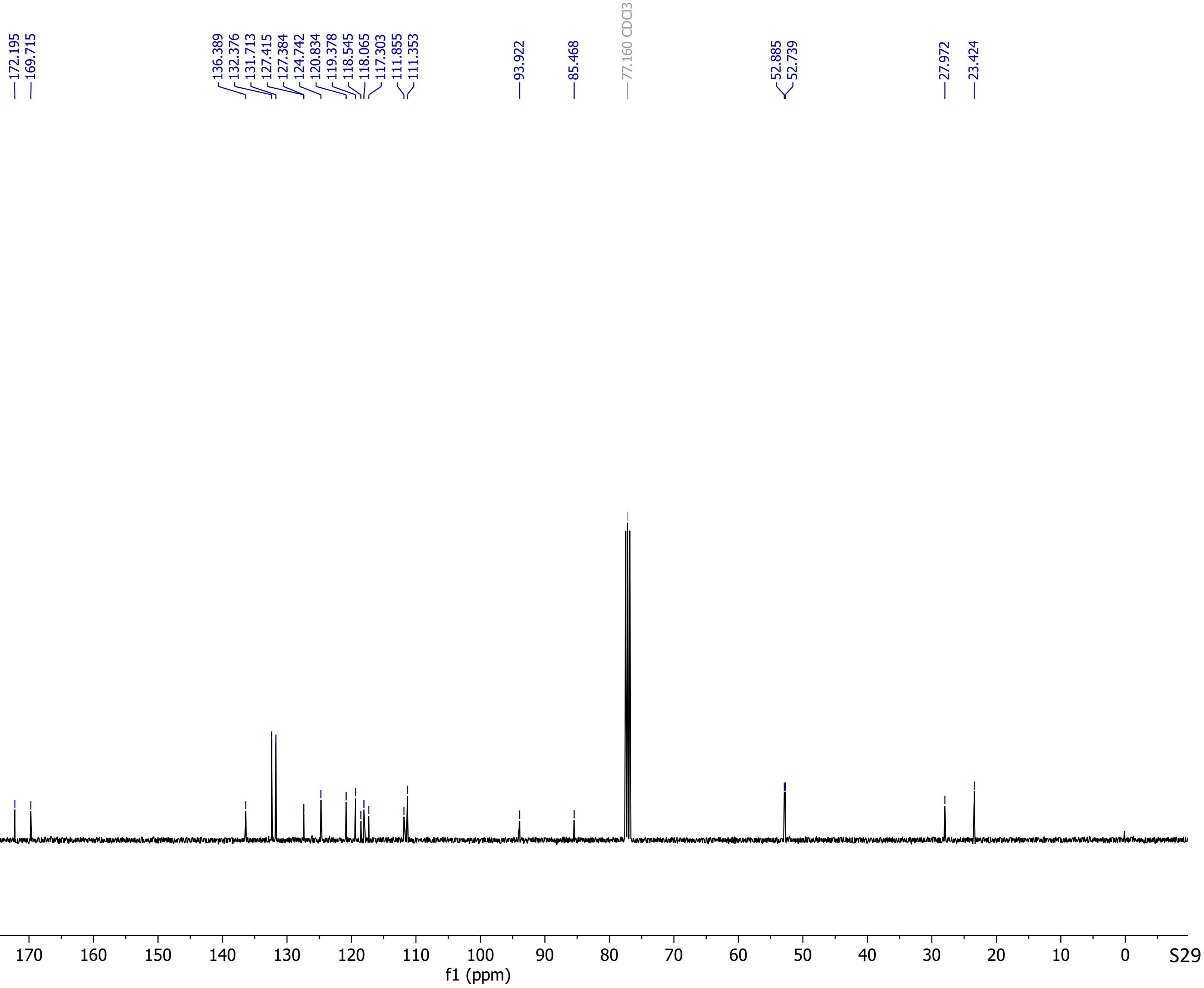

^{1}H NMR (400 MHz, $CDCl_3$)

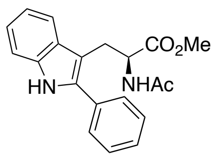

8a

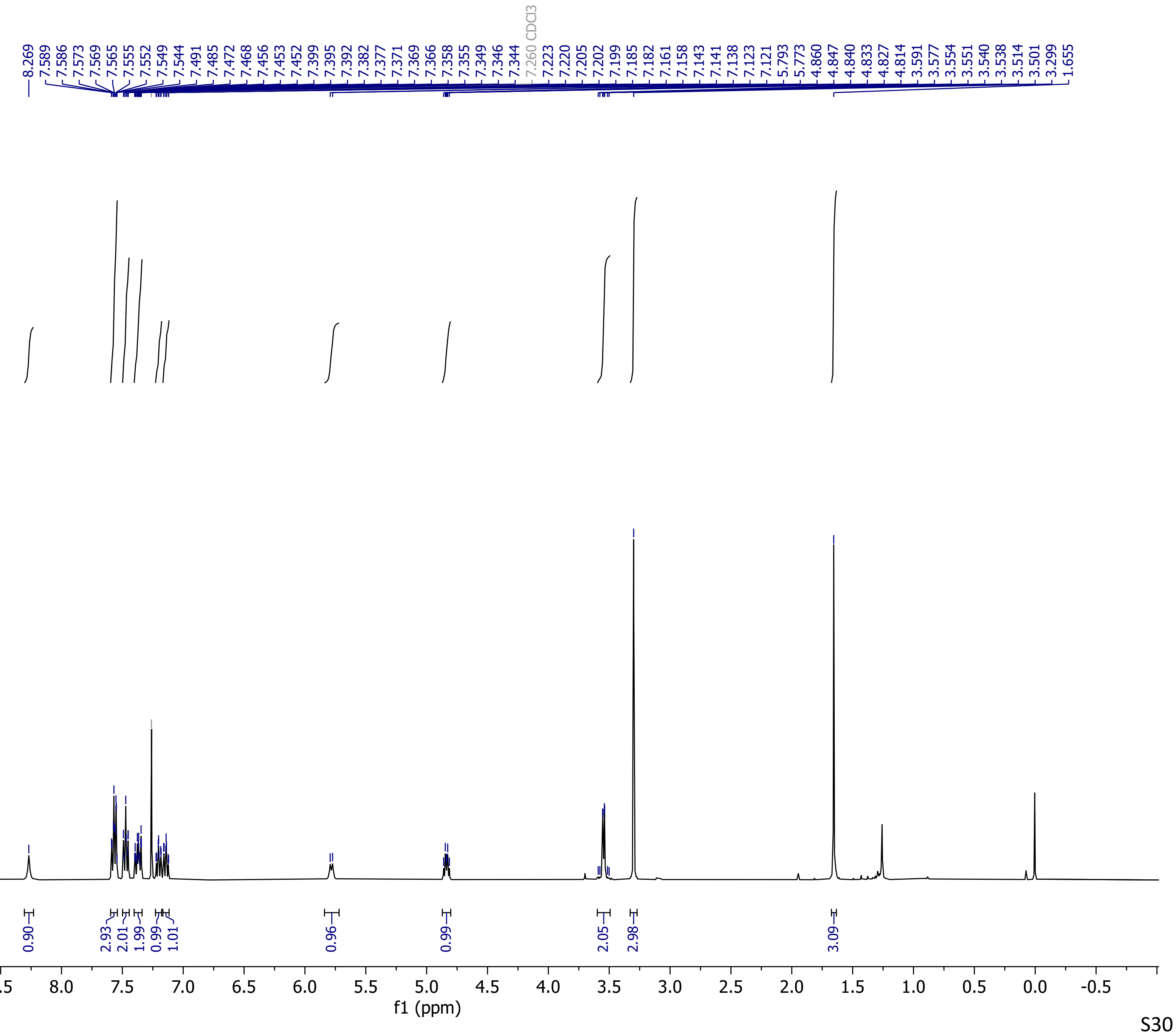

$^{13}C\{^{1}H\}$ NMR (101 MHz, $CDCl_3$)

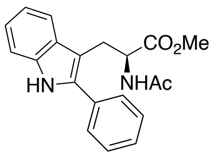

8a

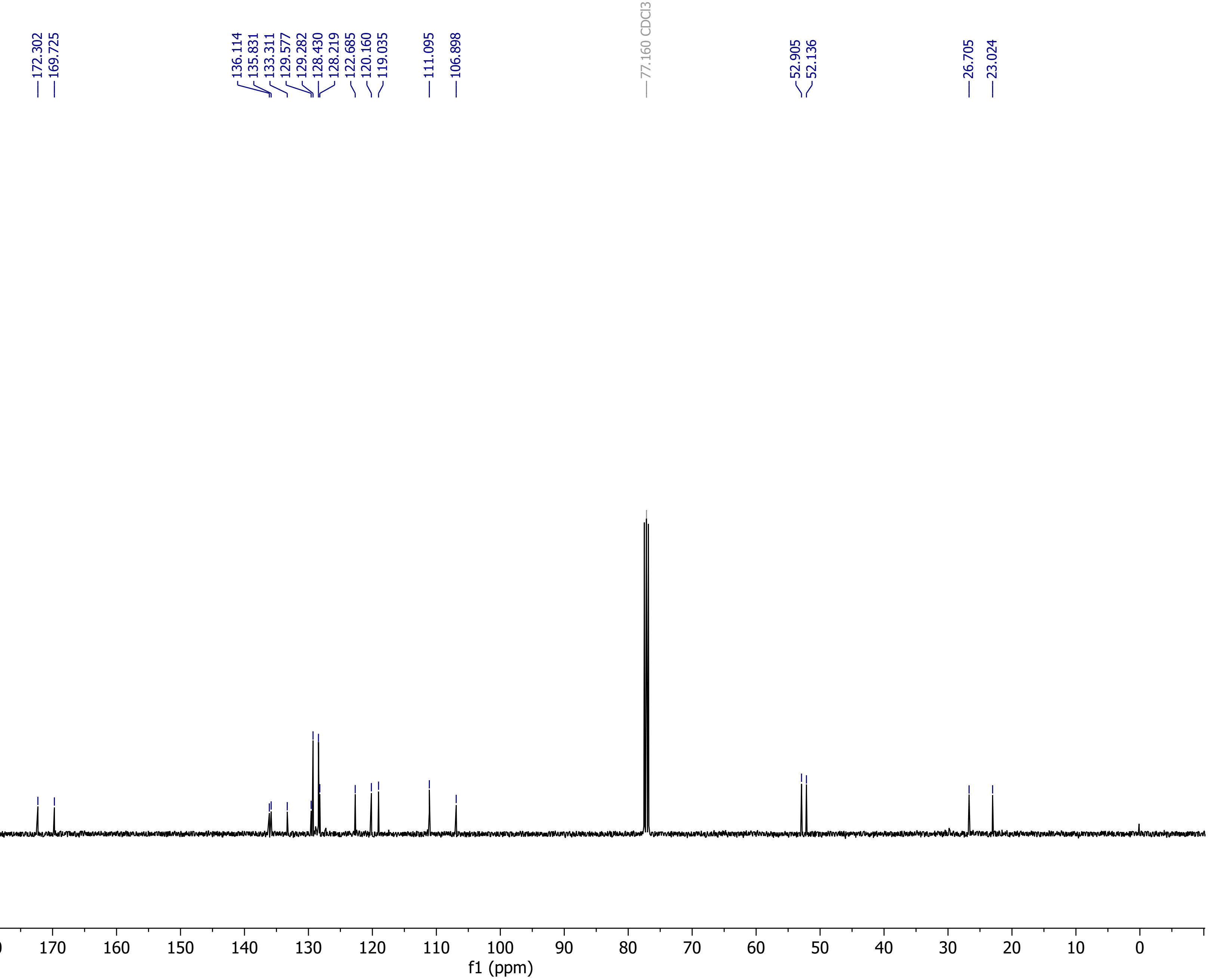

^{1}H NMR (400 MHz, $CDCl_3$)

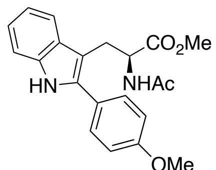

8b

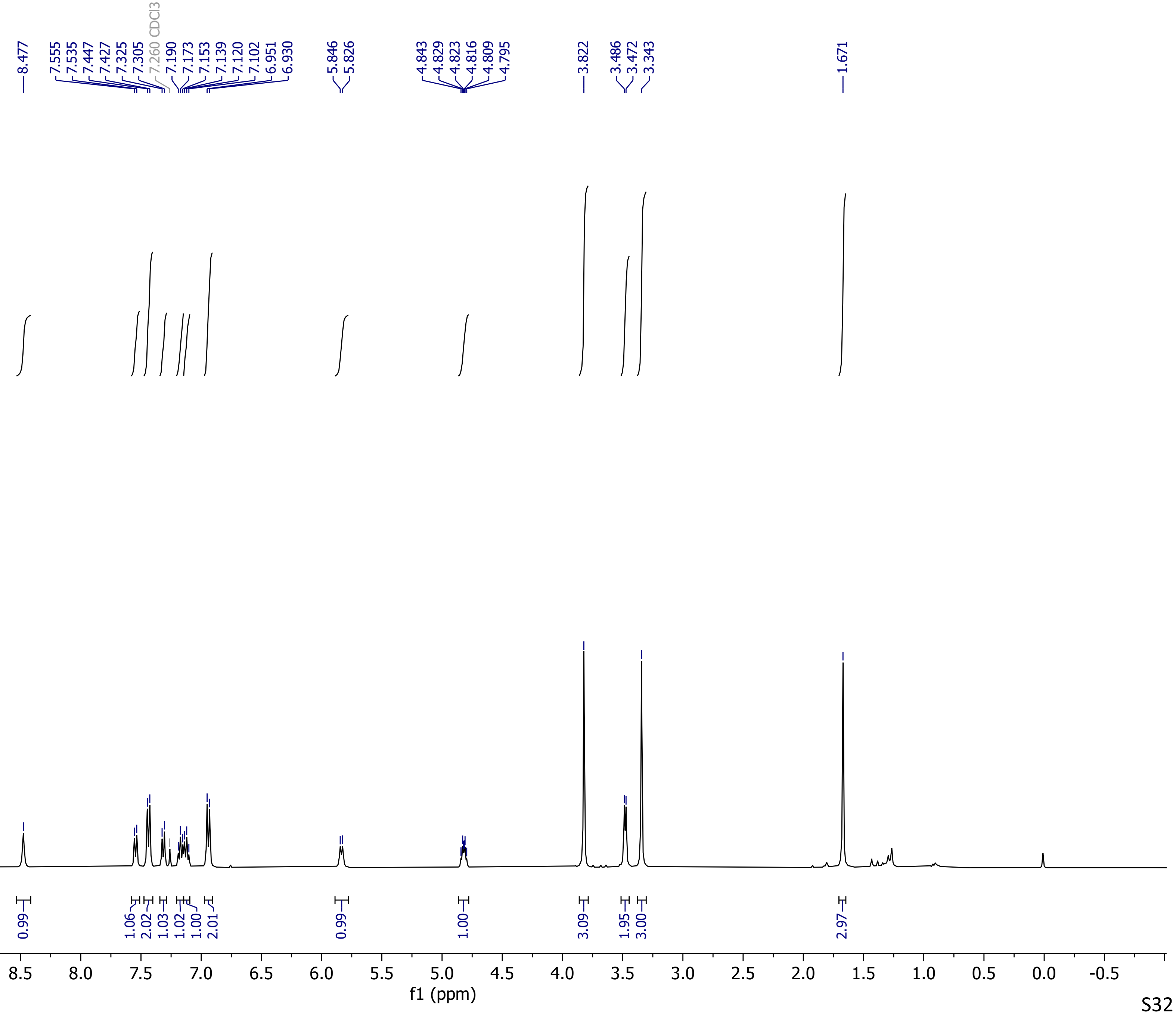

$^{13}C\{^1H\}$ NMR (101 MHz, $CDCl_3$)

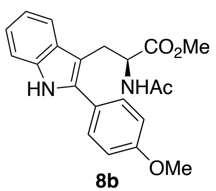

8b

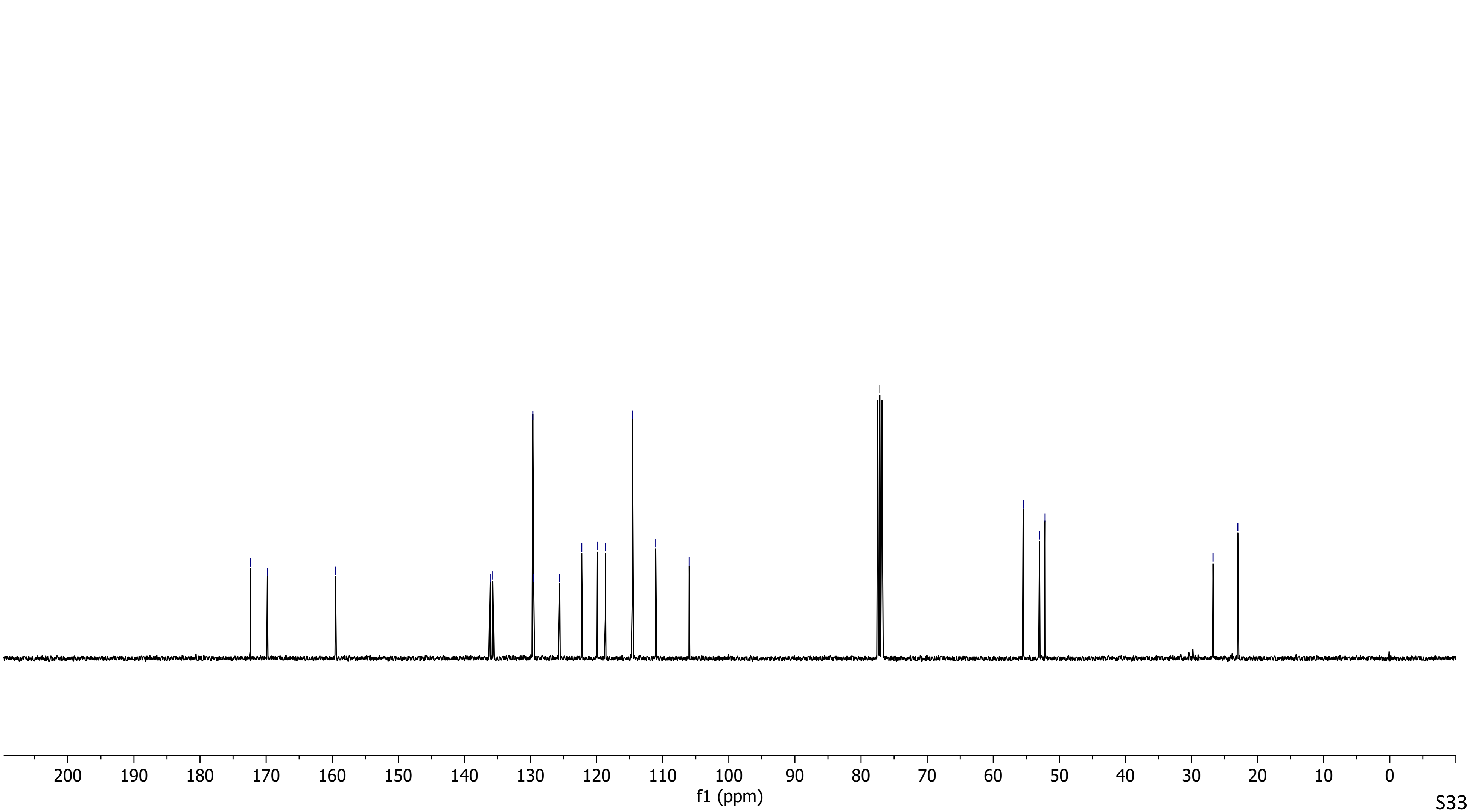

^{1}H NMR (400 MHz, $CDCl_3$)

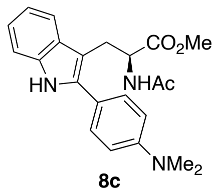

8c

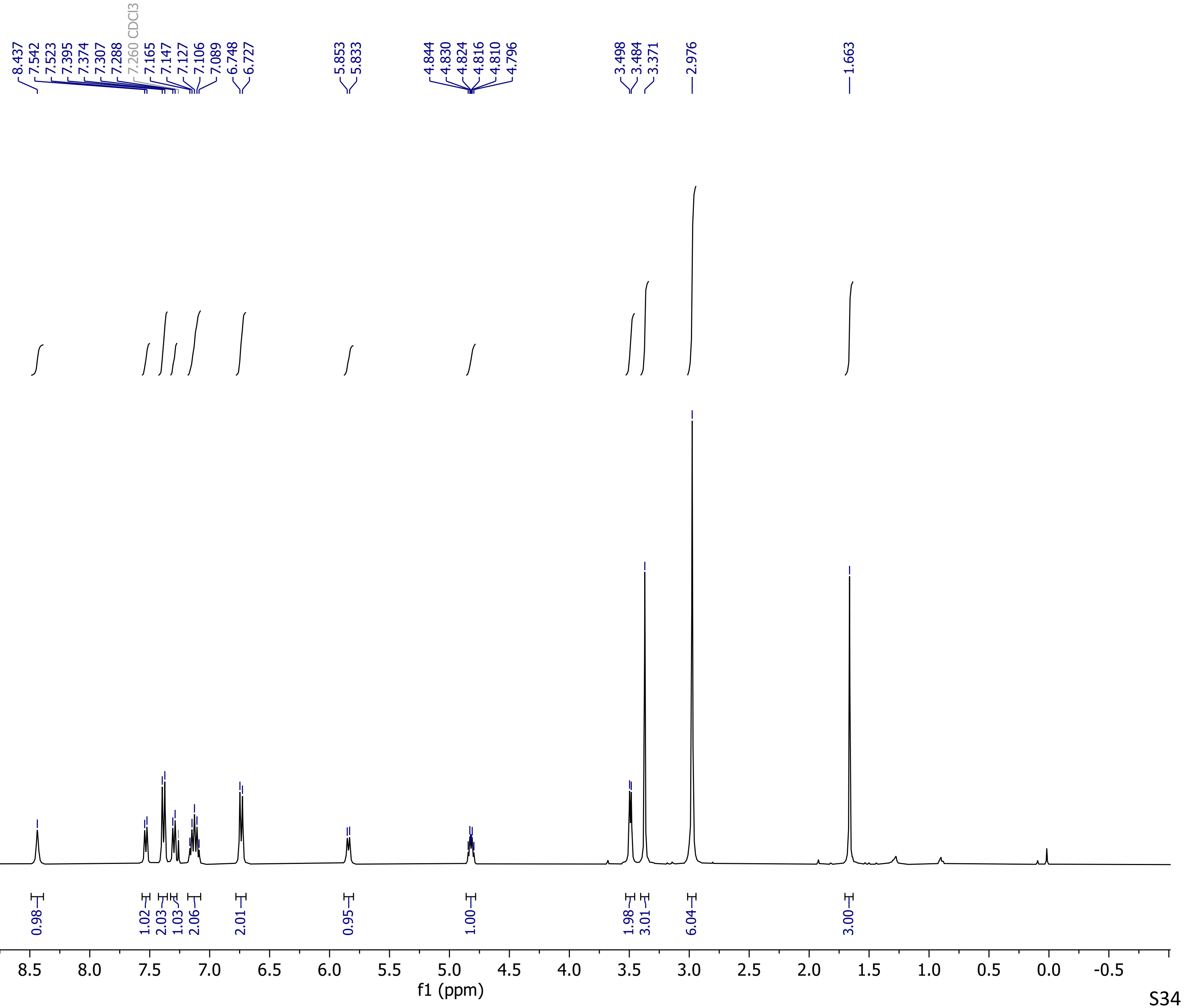

$^{13}C\{^1H\}$ NMR (101 MHz, $CDCl_3$)

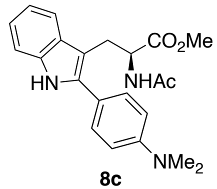

8c

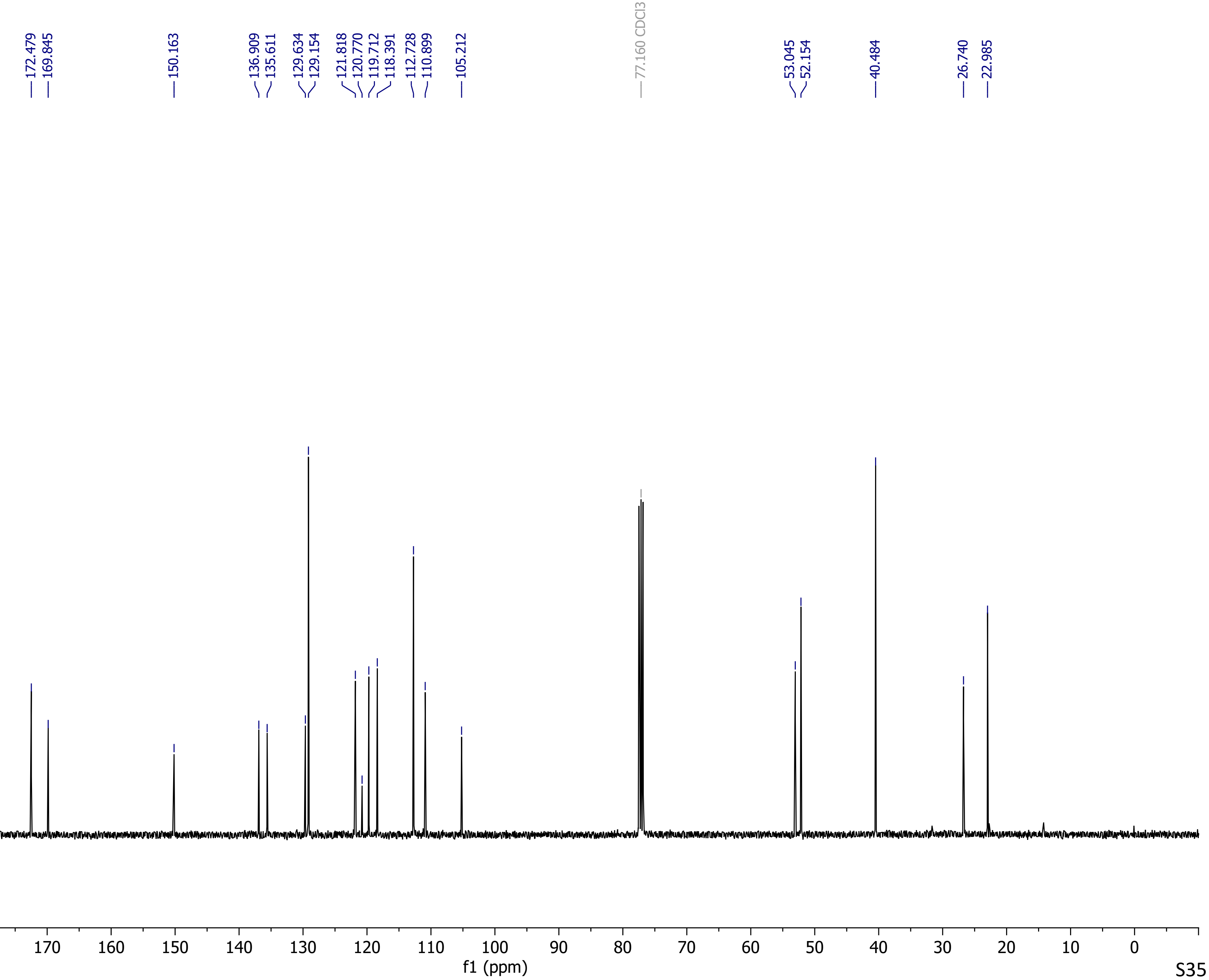

^{1}H NMR (400 MHz, $CDCl_3$)

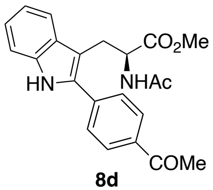

8d

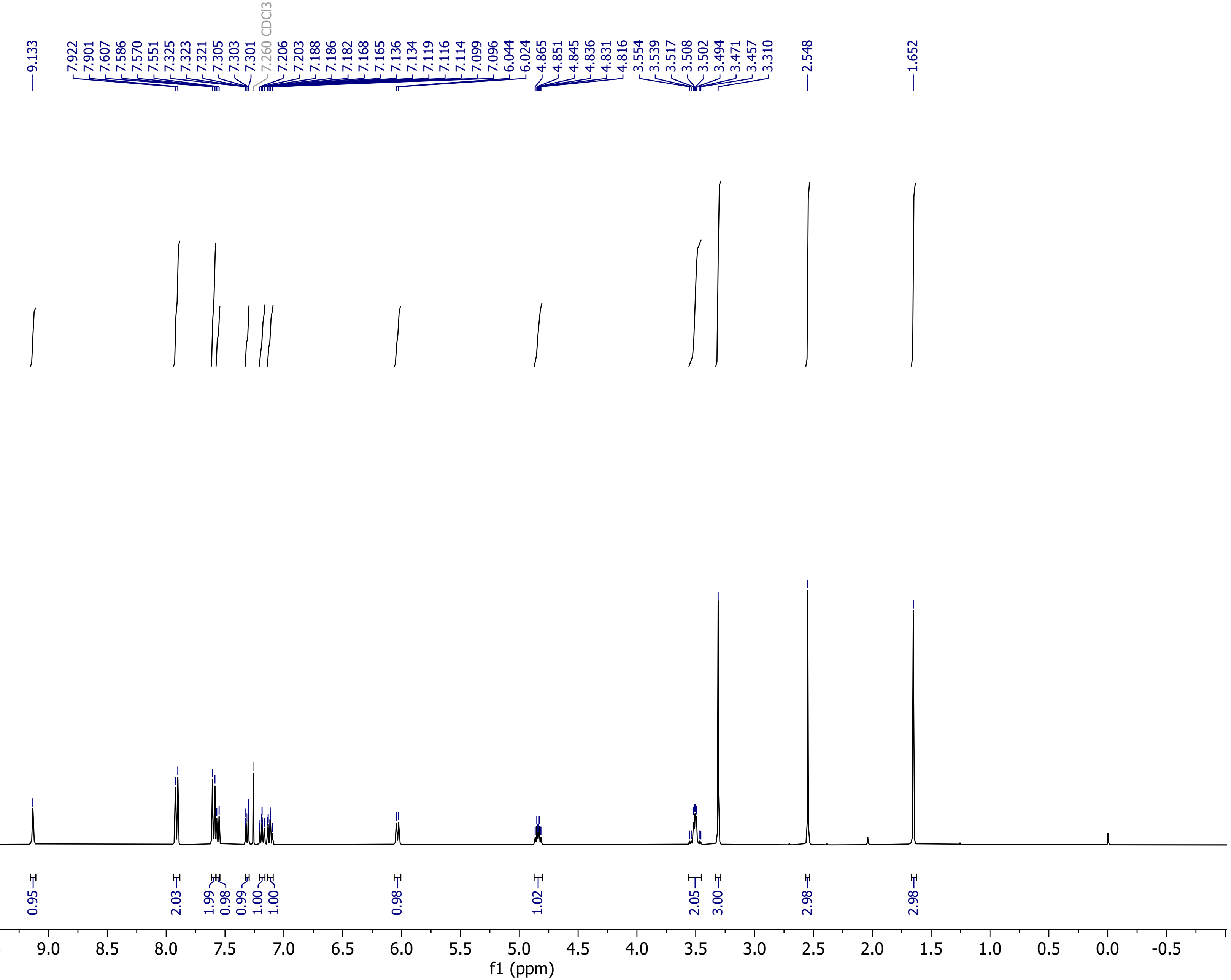

$^{13}C\{^{1}H\}$ NMR (101 MHz, $CDCl_3$)

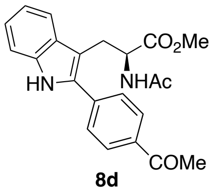

8d

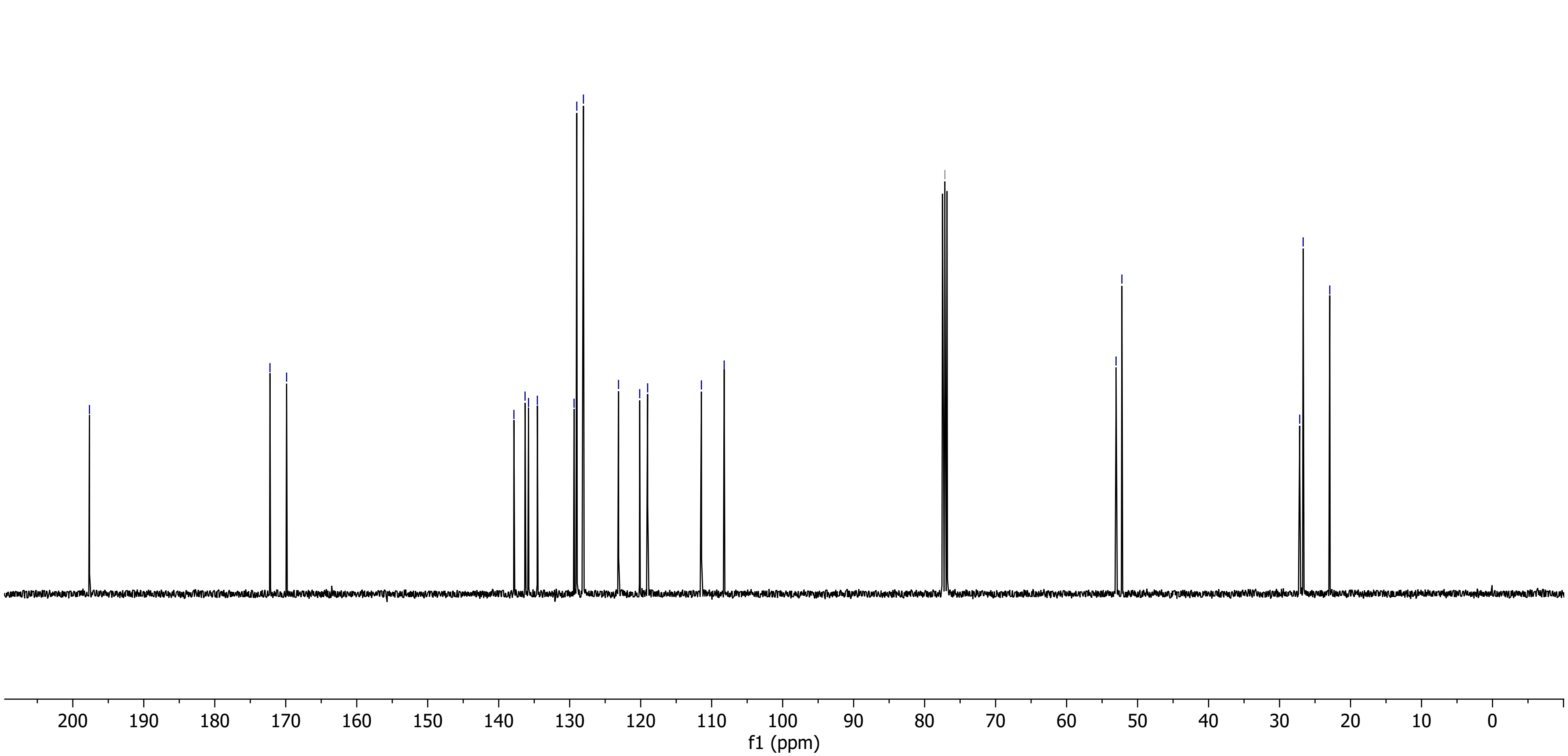

^{1}H NMR (400 MHz, $CDCl_3$)

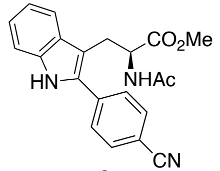

8e

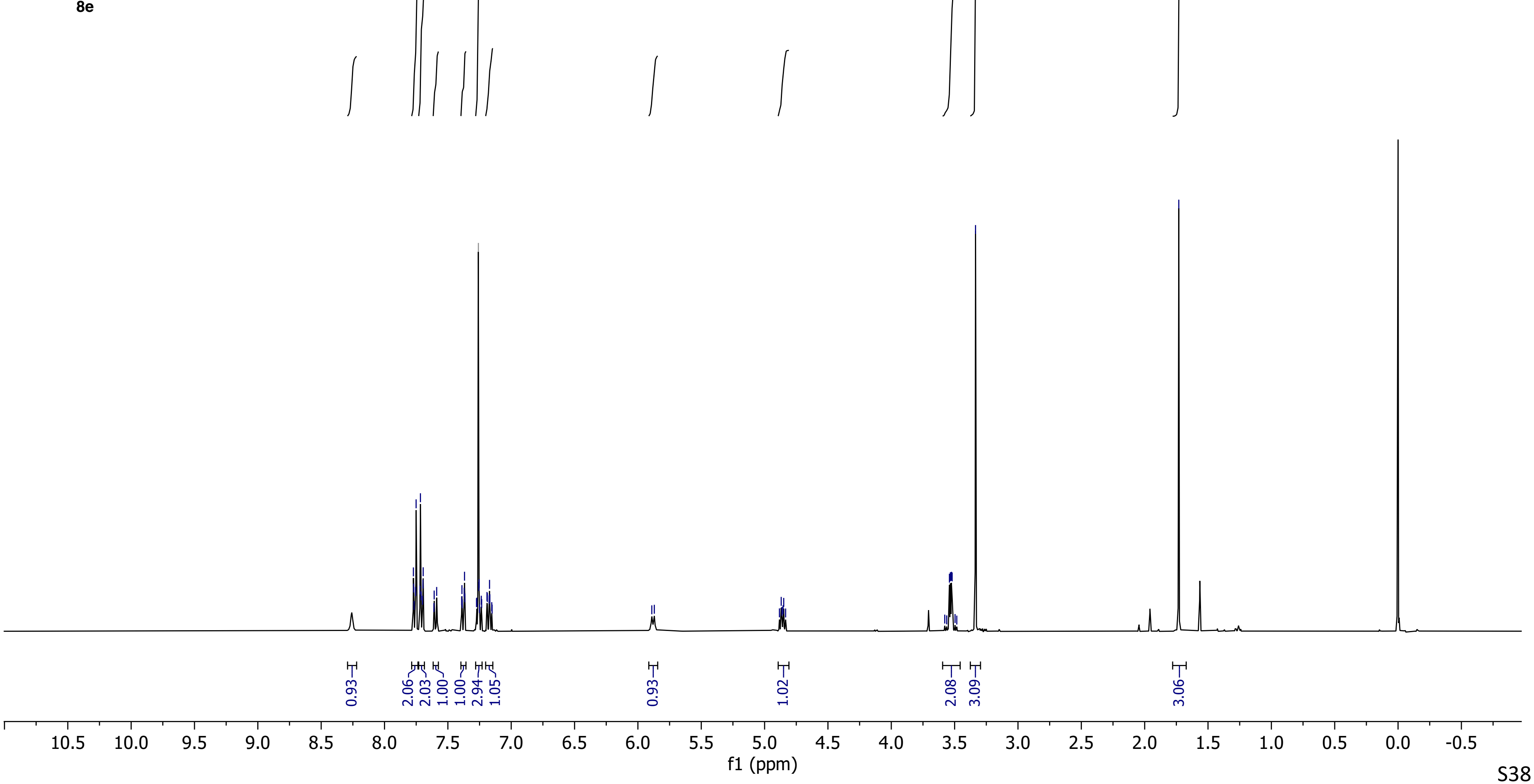

$^{13}C\{^1H\}$ **NMR (101 MHz, CDCl$_3$)**

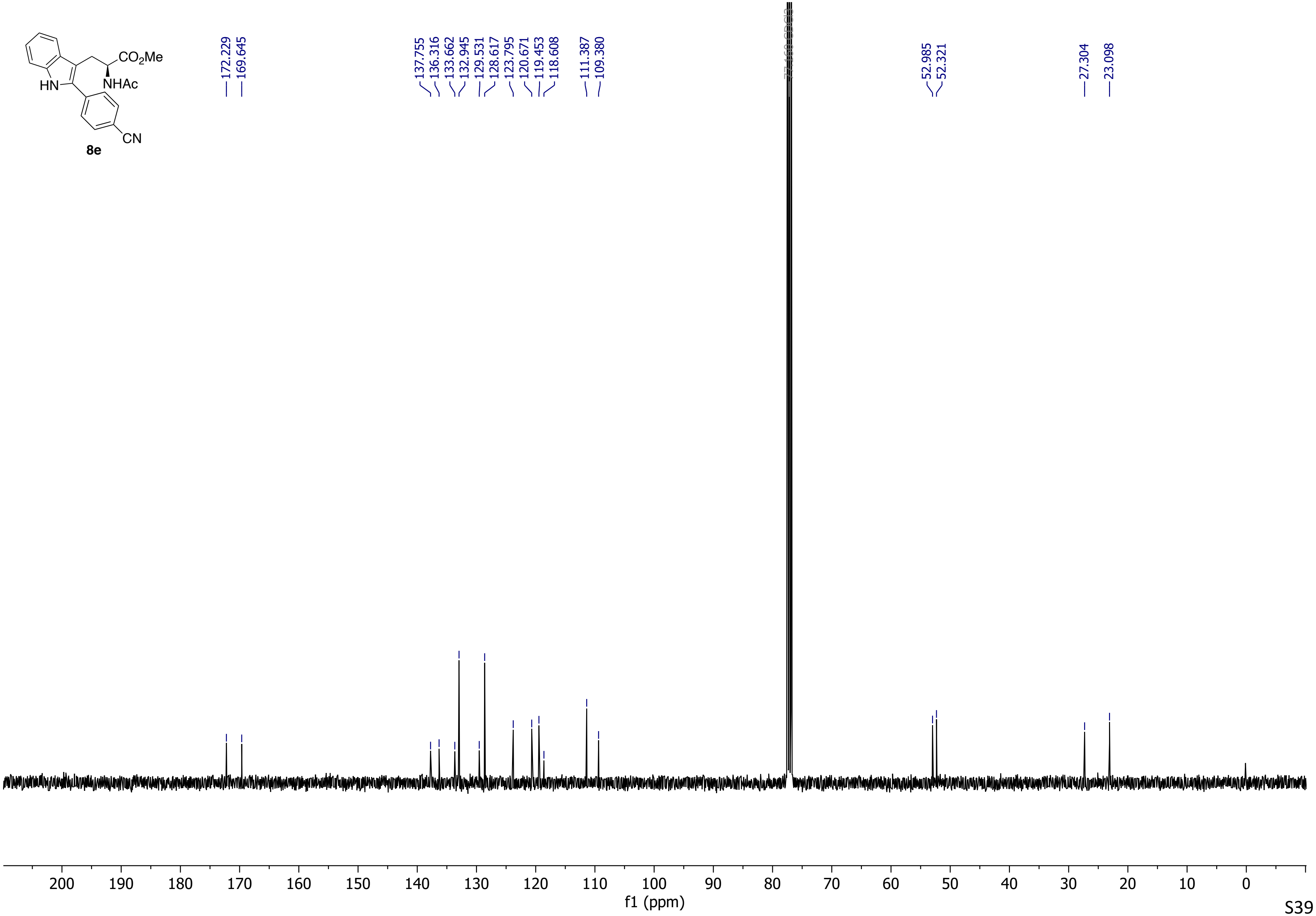

^{1}H NMR (400 MHz, $CDCl_3$)

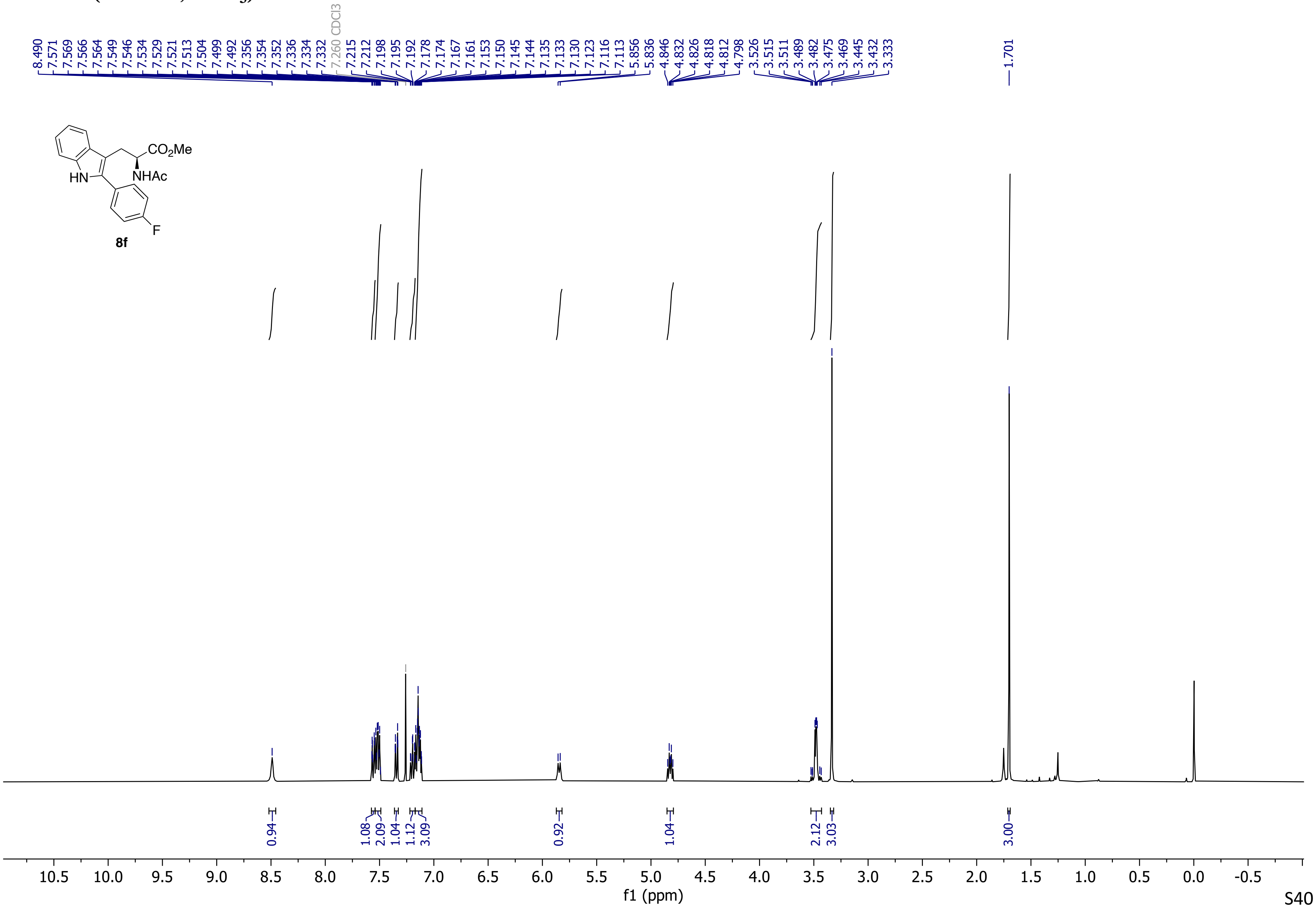

$^{13}C\{^1H\}$ NMR (101 MHz, $CDCl_3$)

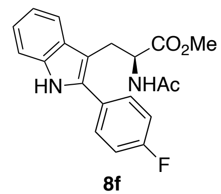

8f

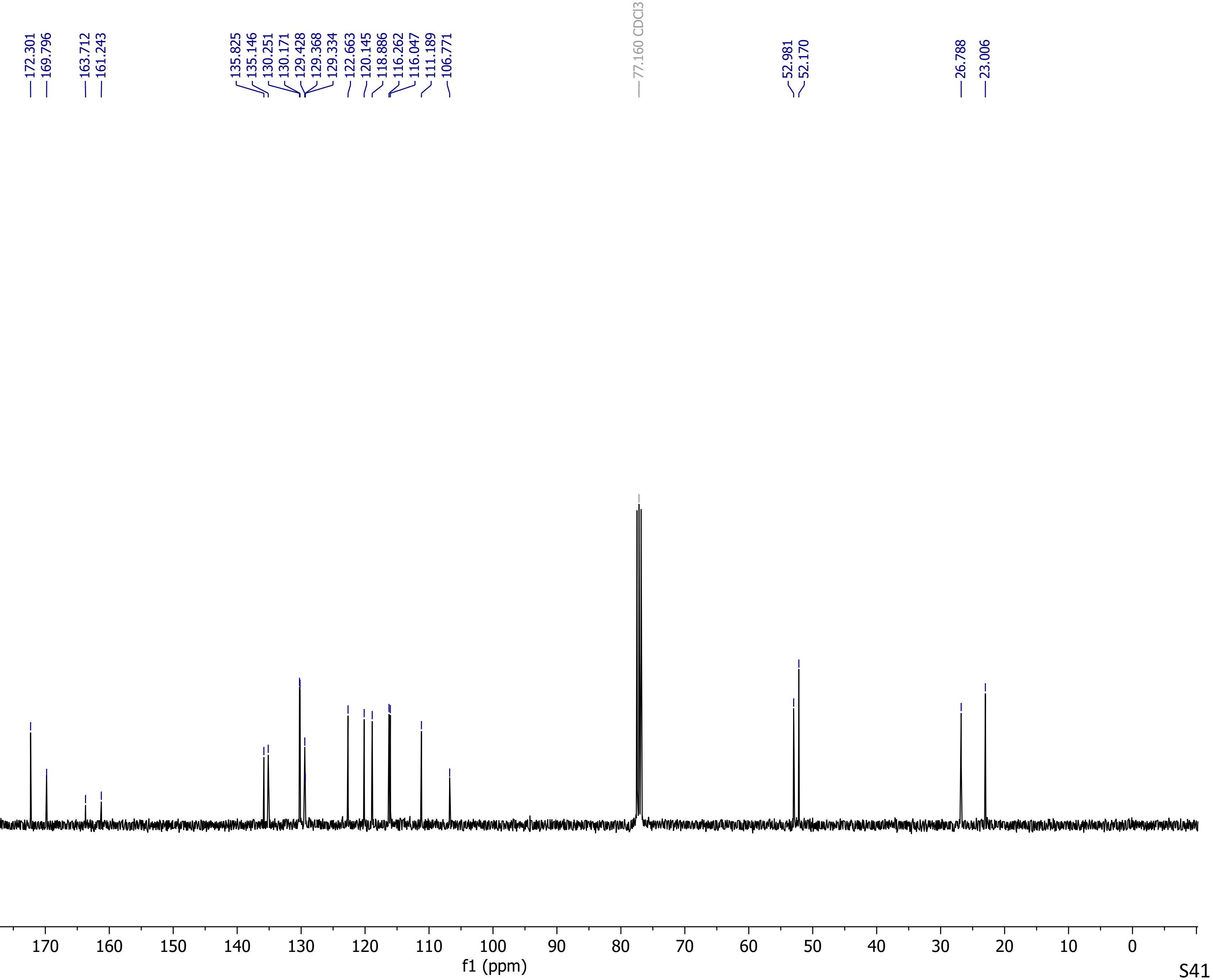

^{1}H NMR (400 MHz, $CDCl_3$)

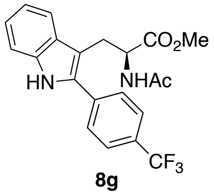

8g

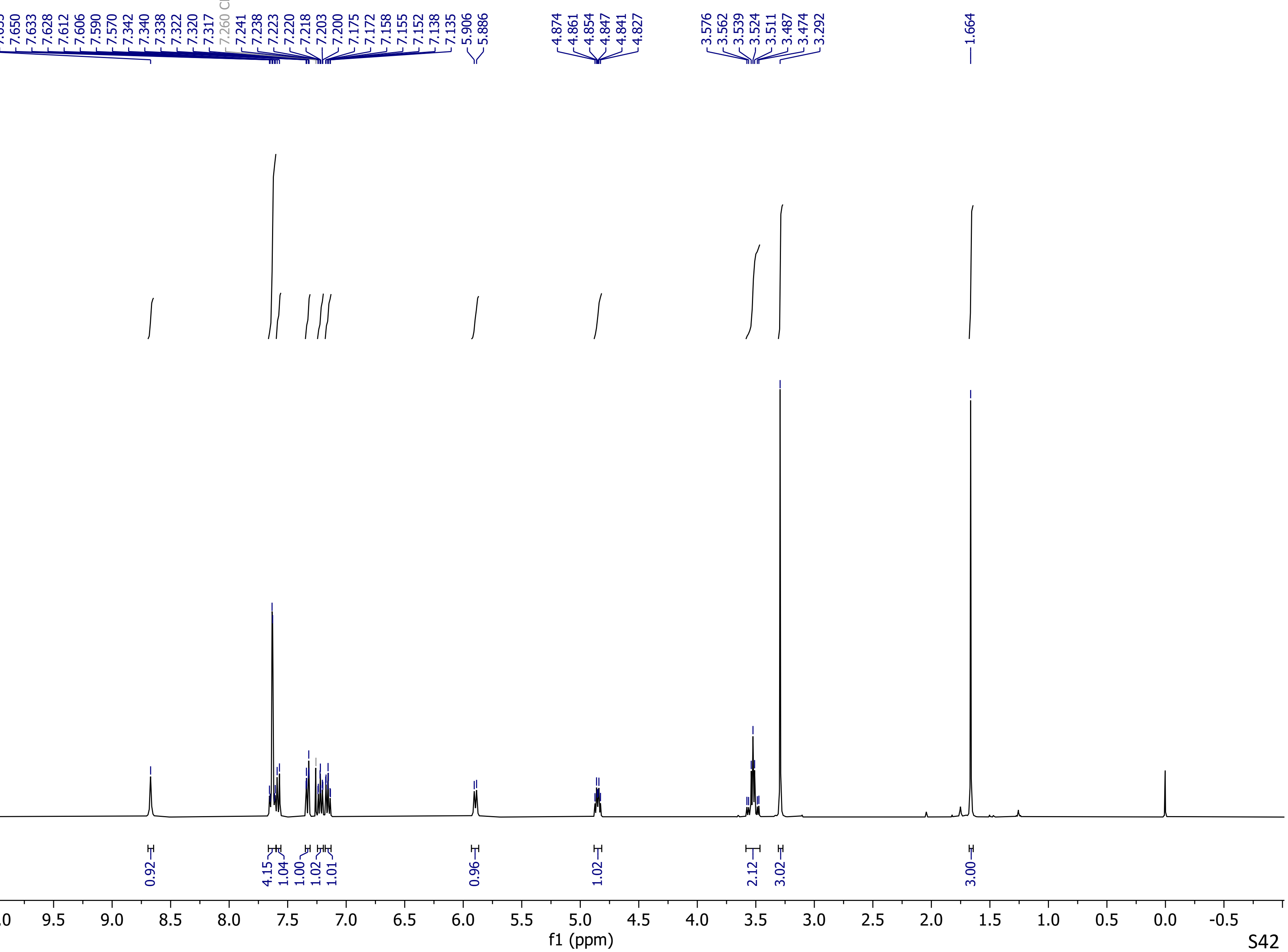

$^{13}C\{^{1}H\}$ NMR (101 MHz, $CDCl_3$)

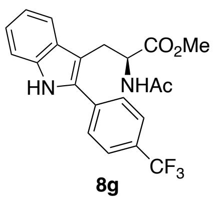

8g

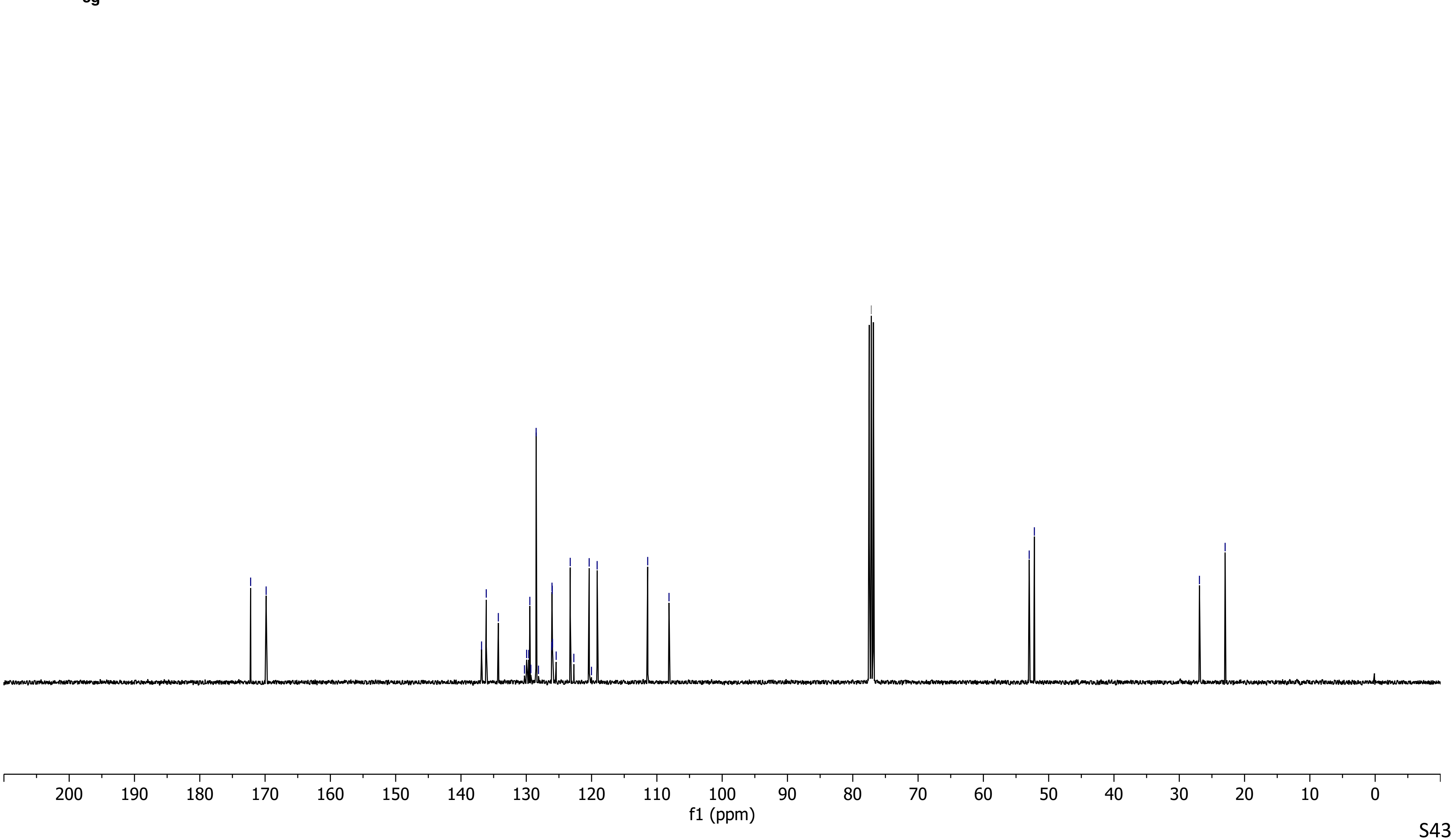

^{1}H NMR (400 MHz, $CDCl_3$)

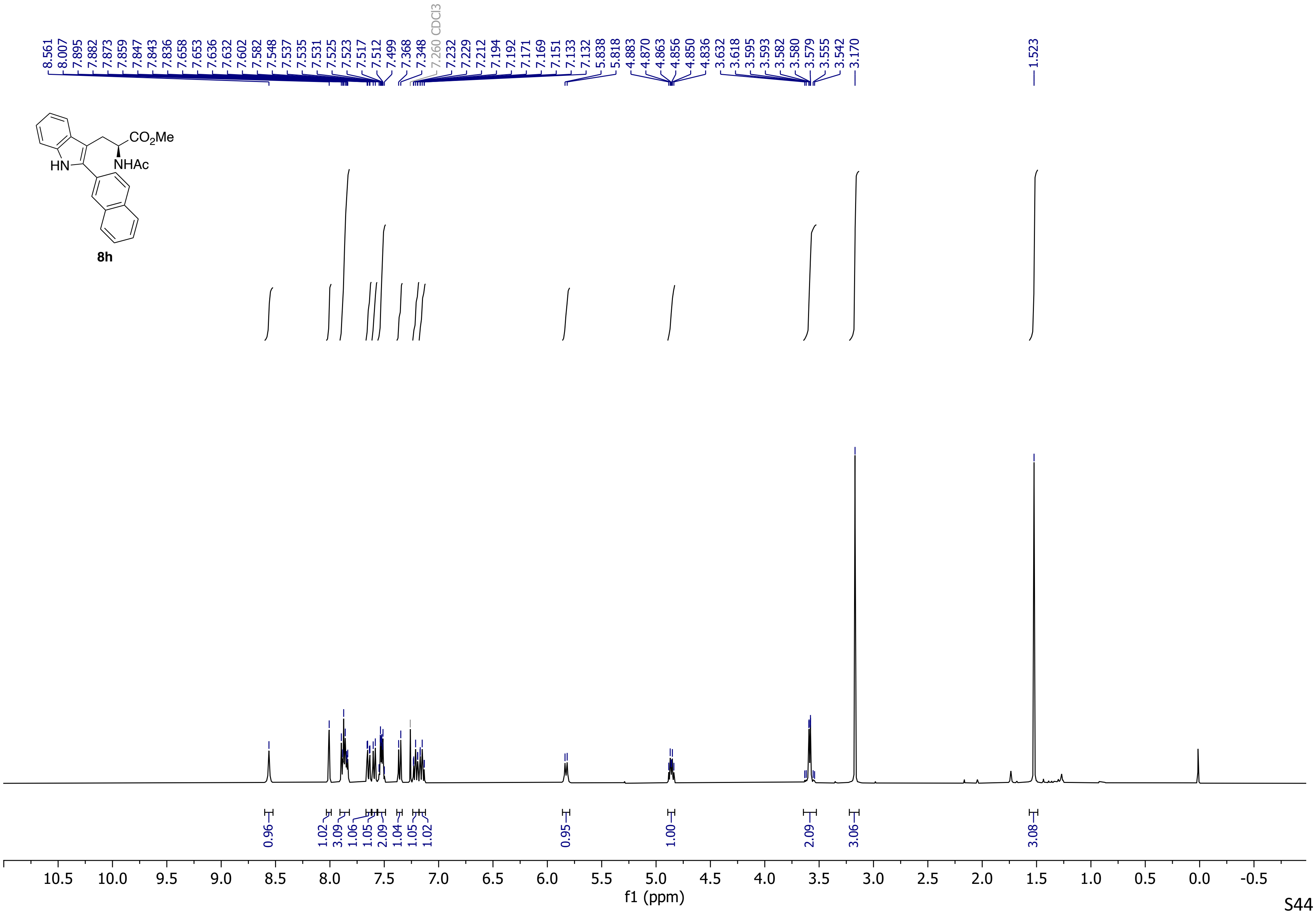

$^{13}C\{^{1}H\}$ NMR (101 MHz, $CDCl_3$)

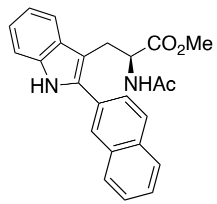

8h

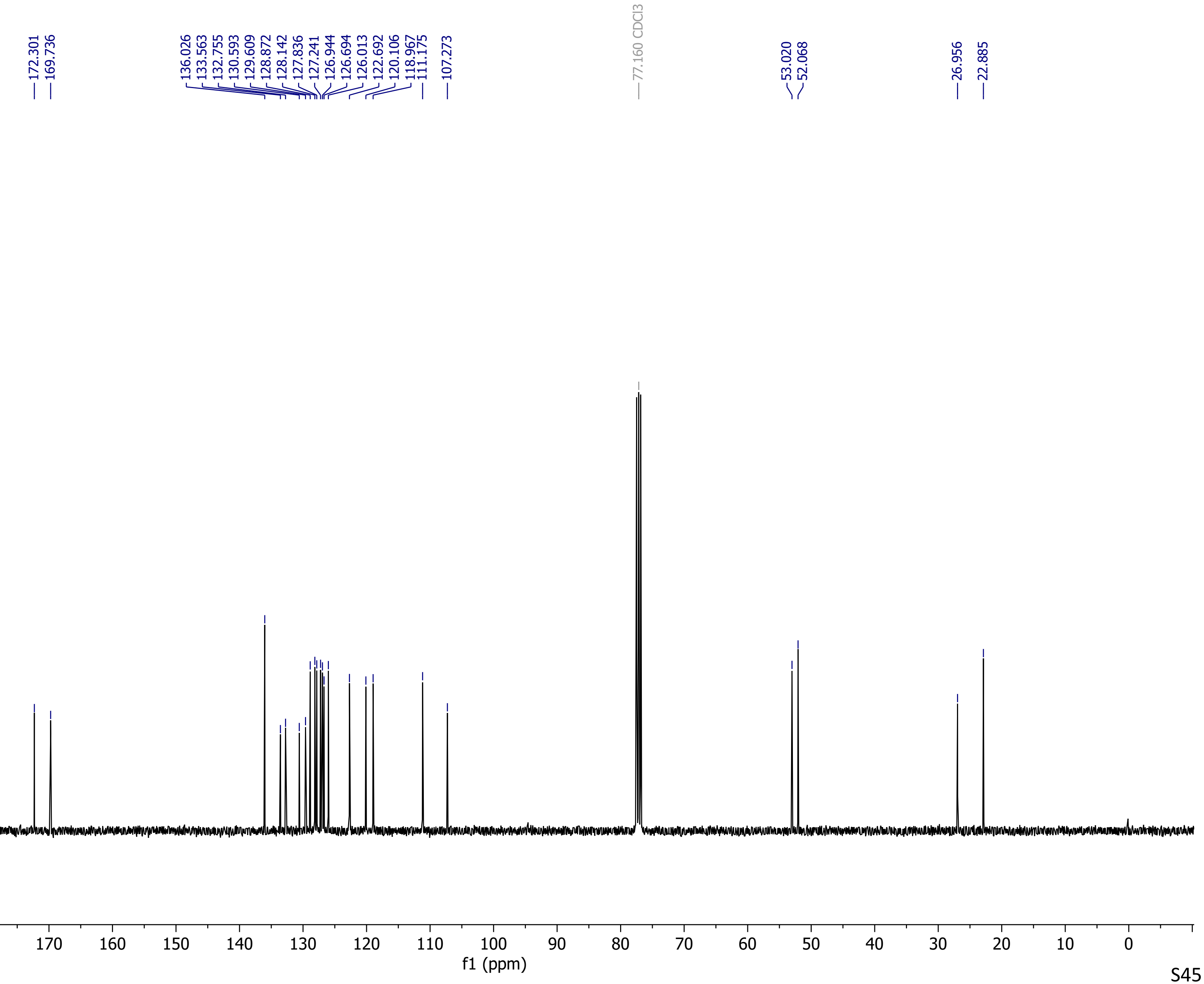

^{1}H NMR (400 MHz, $CDCl_3$)

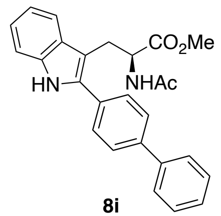

8i

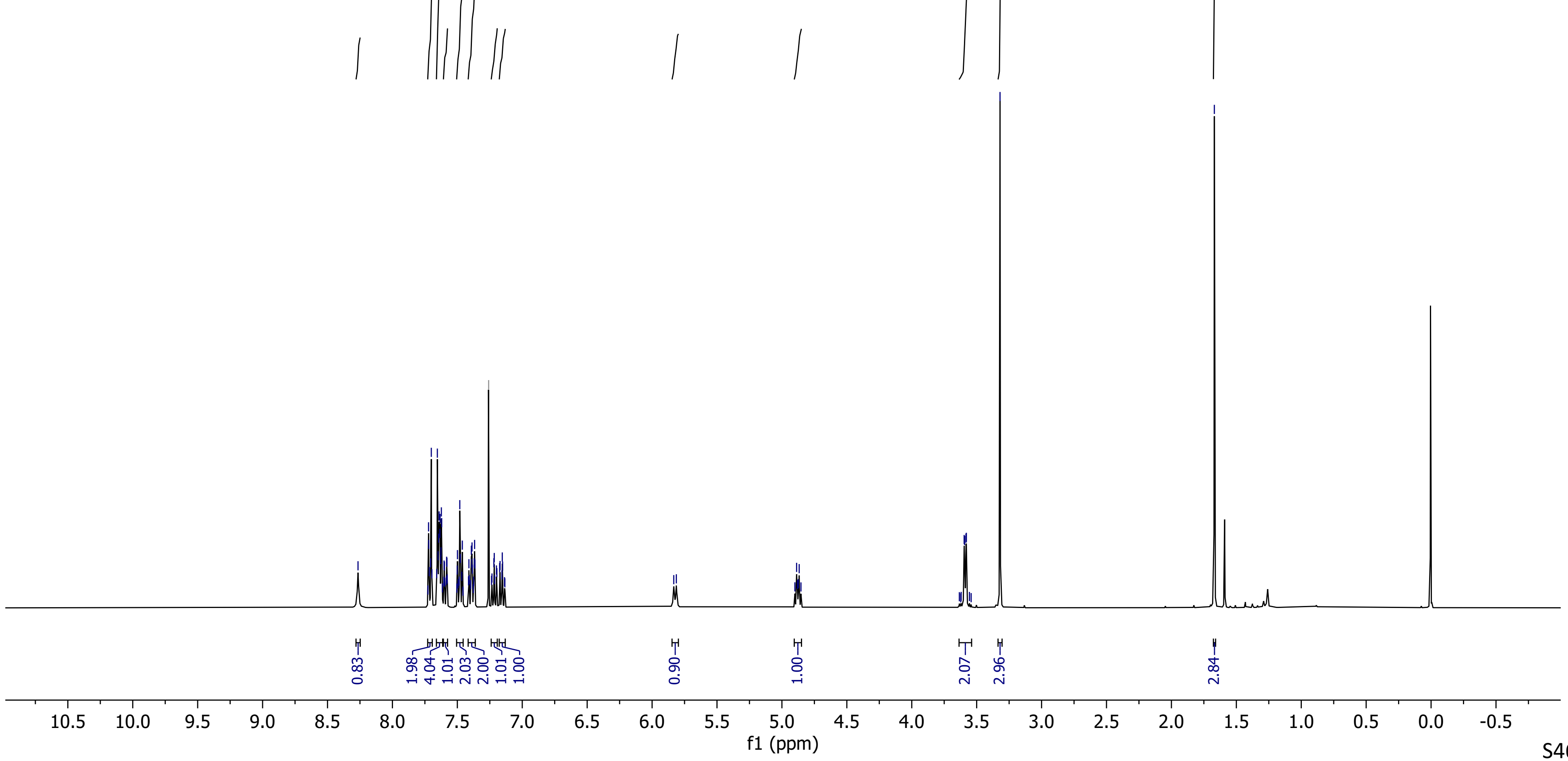

$^{13}C\{^{1}H\}$ NMR (101 MHz, $CDCl_3$)

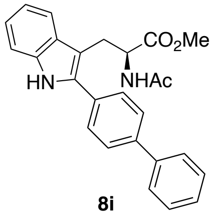

8i

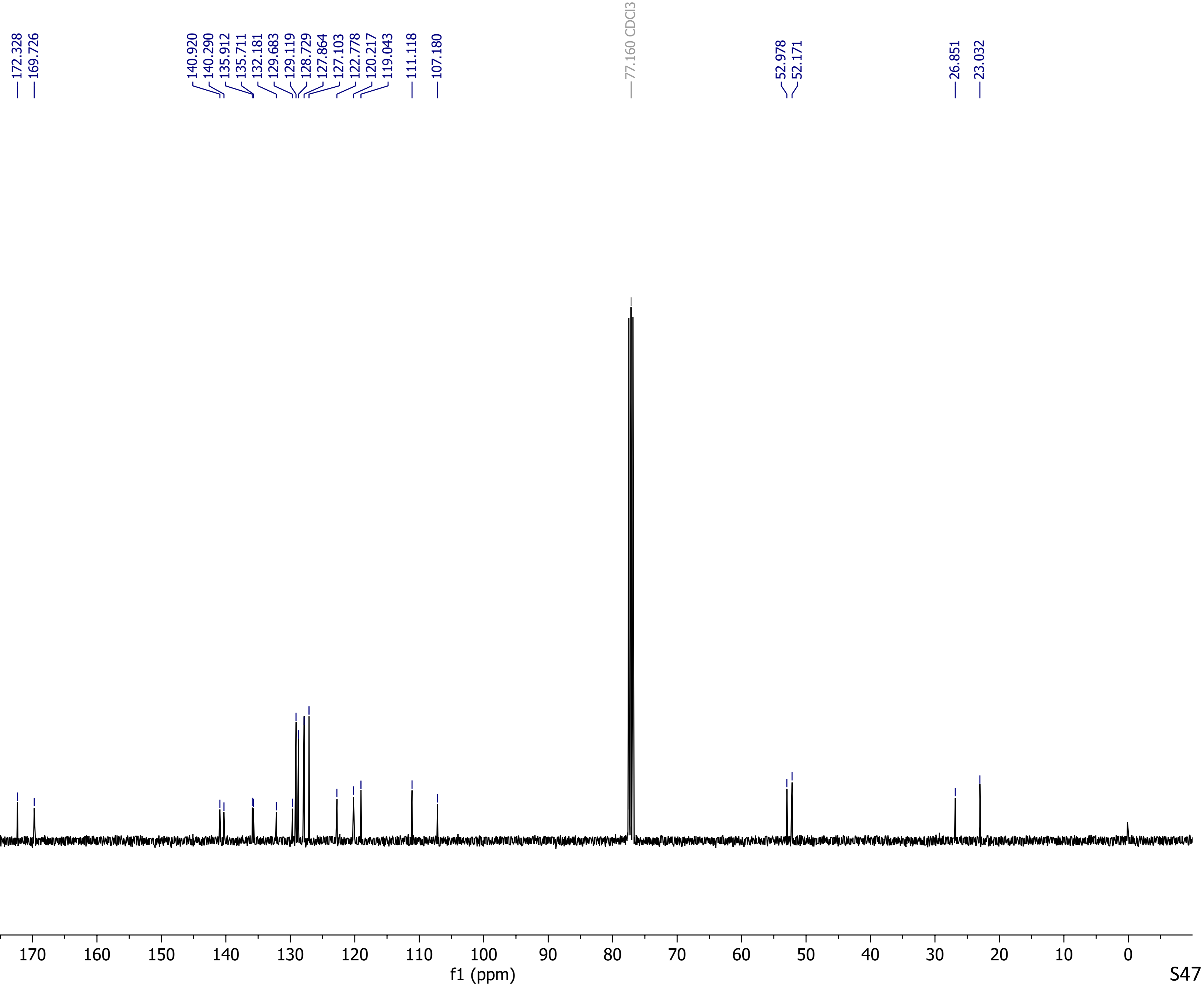

^{1}H NMR (400 MHz, CDCl$_3$)

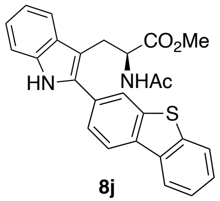

8j

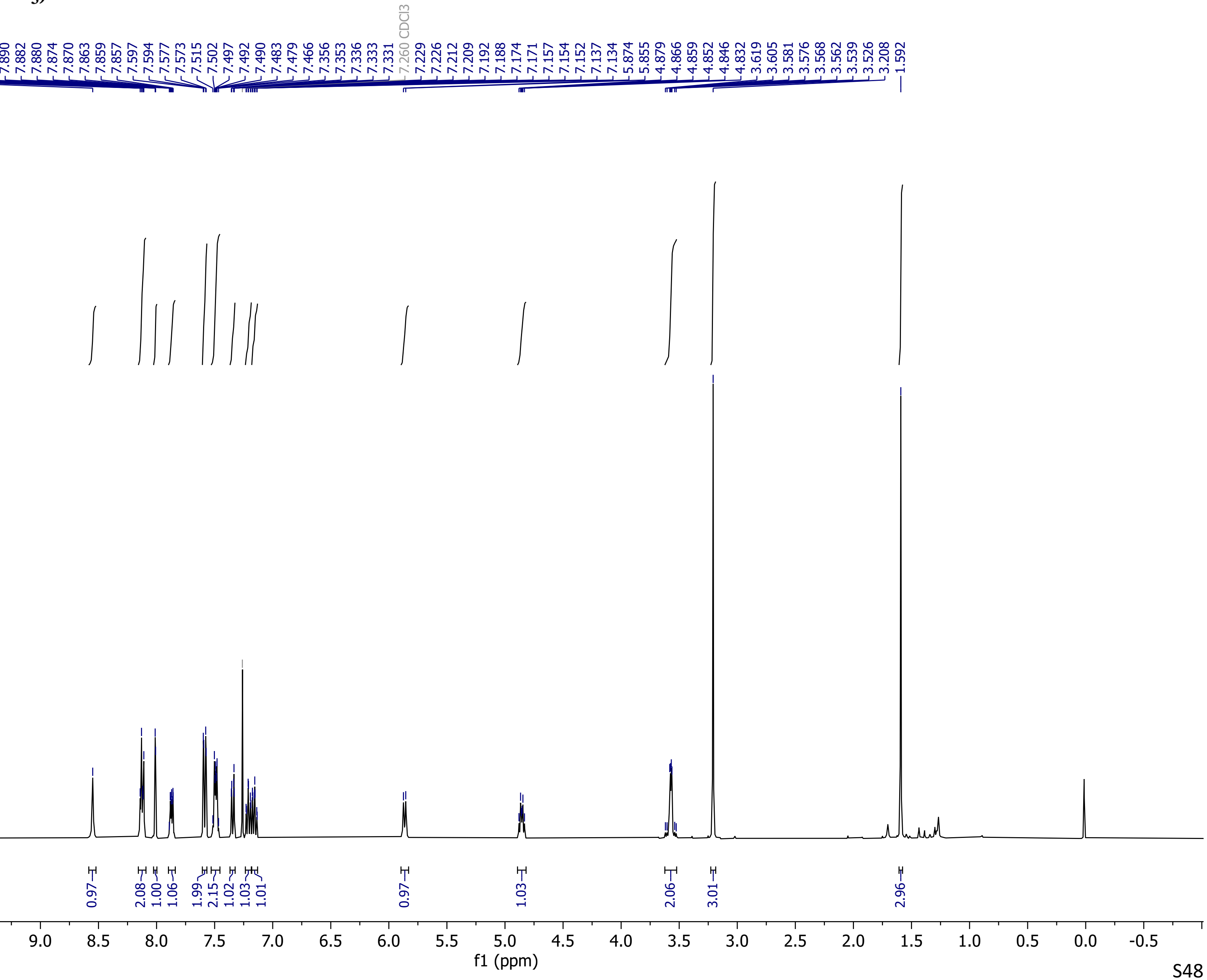

$^{13}C\{^{1}H\}$ NMR (101 MHz, $CDCl_3$)

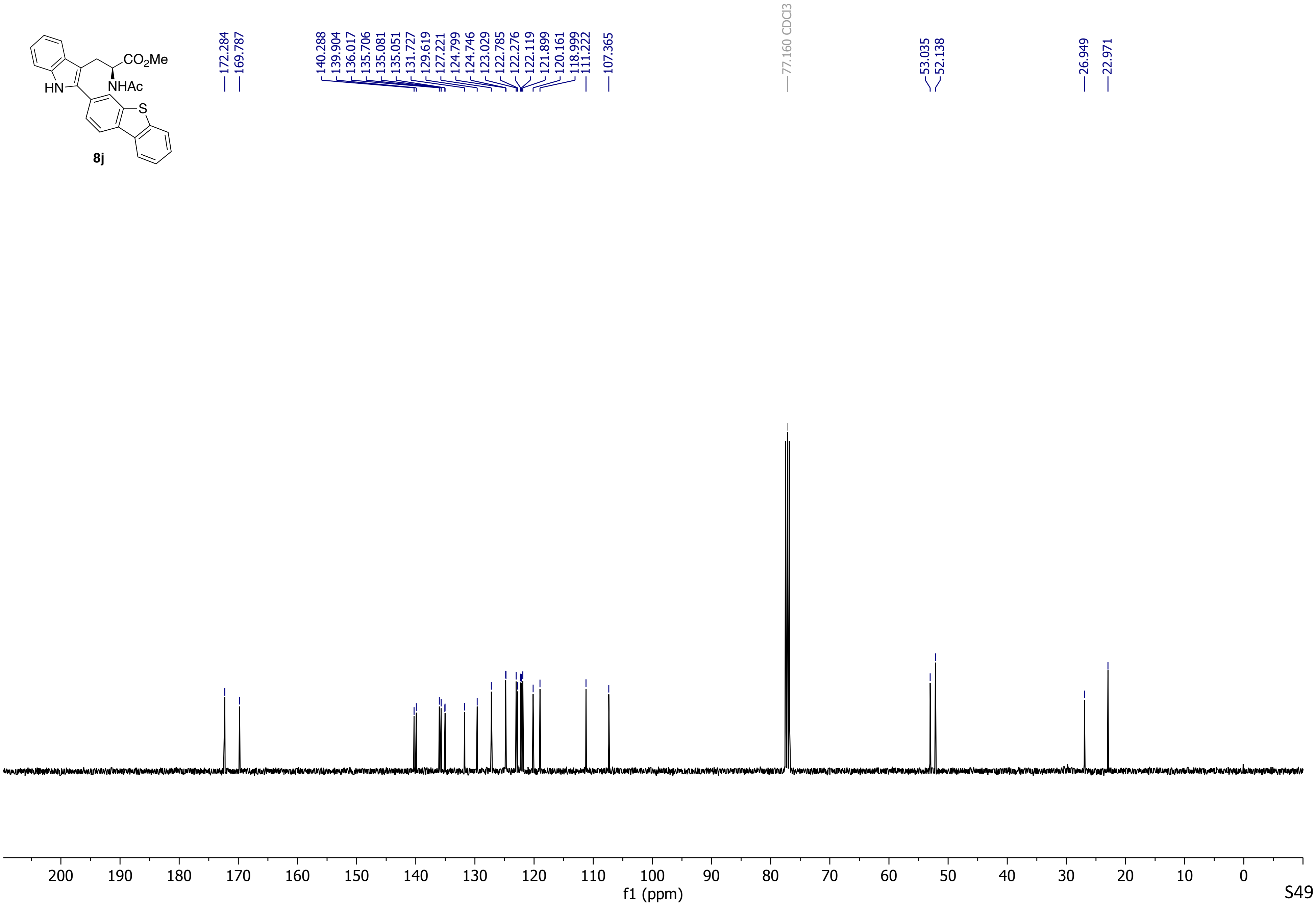

^{1}H NMR (400 MHz, CDCl$_3$)

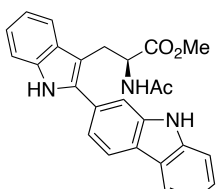

8k

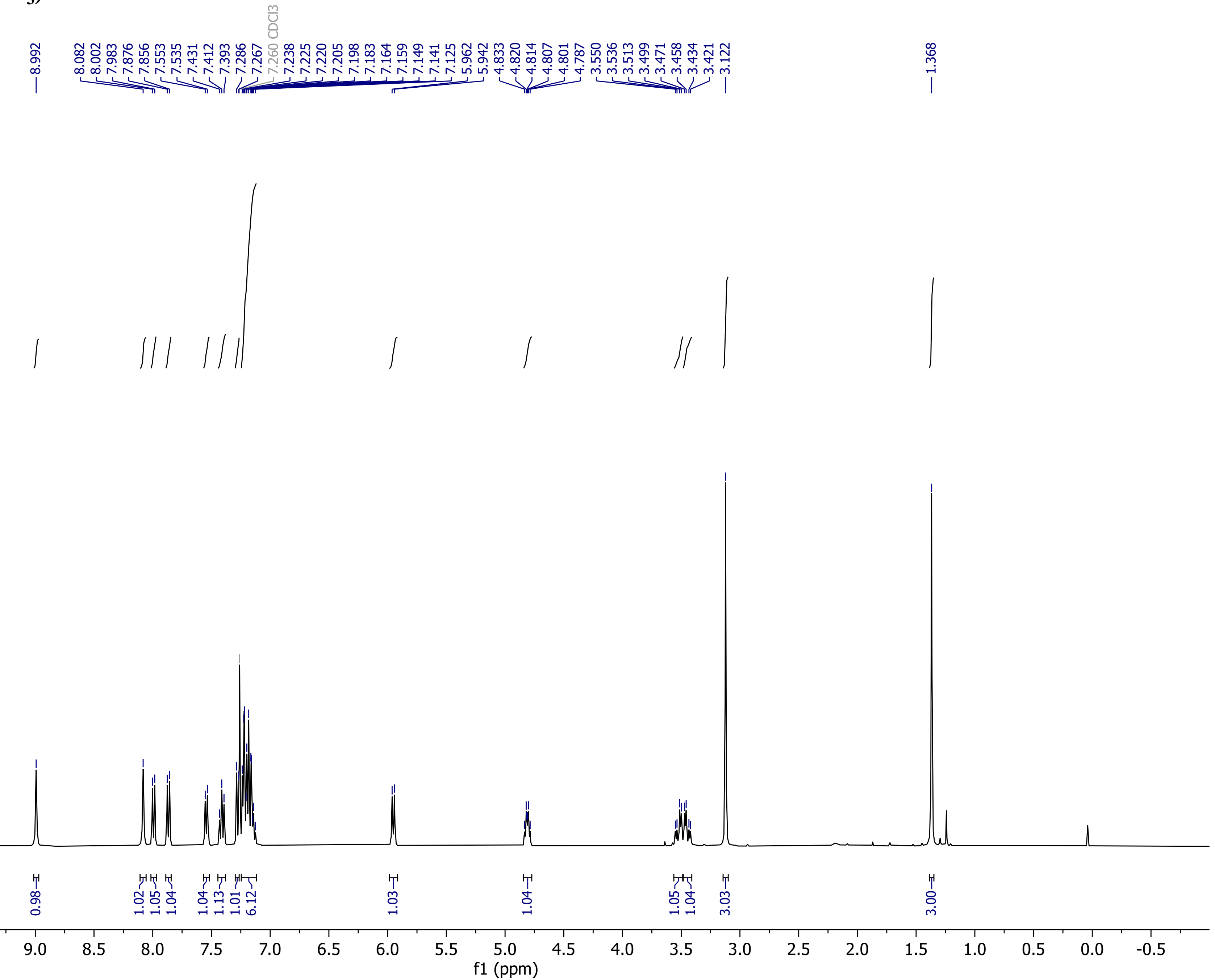

$^{13}C\{^{1}H\}$ NMR (101 MHz, $CDCl_3$)

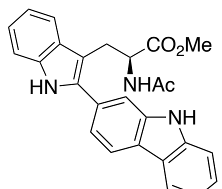

8k

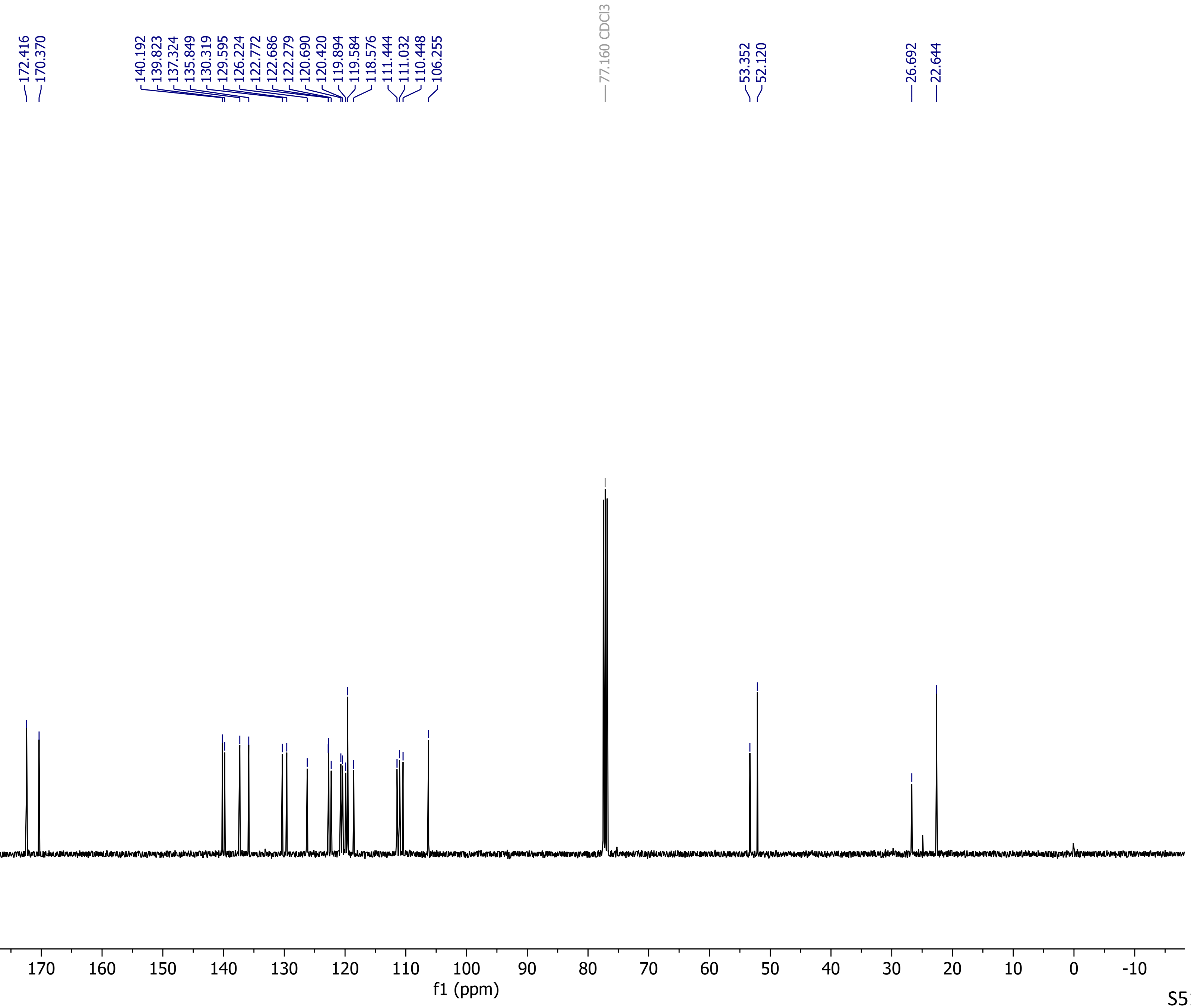

^{1}H NMR (400 MHz, CD_3OD)

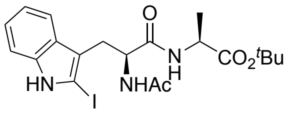

9

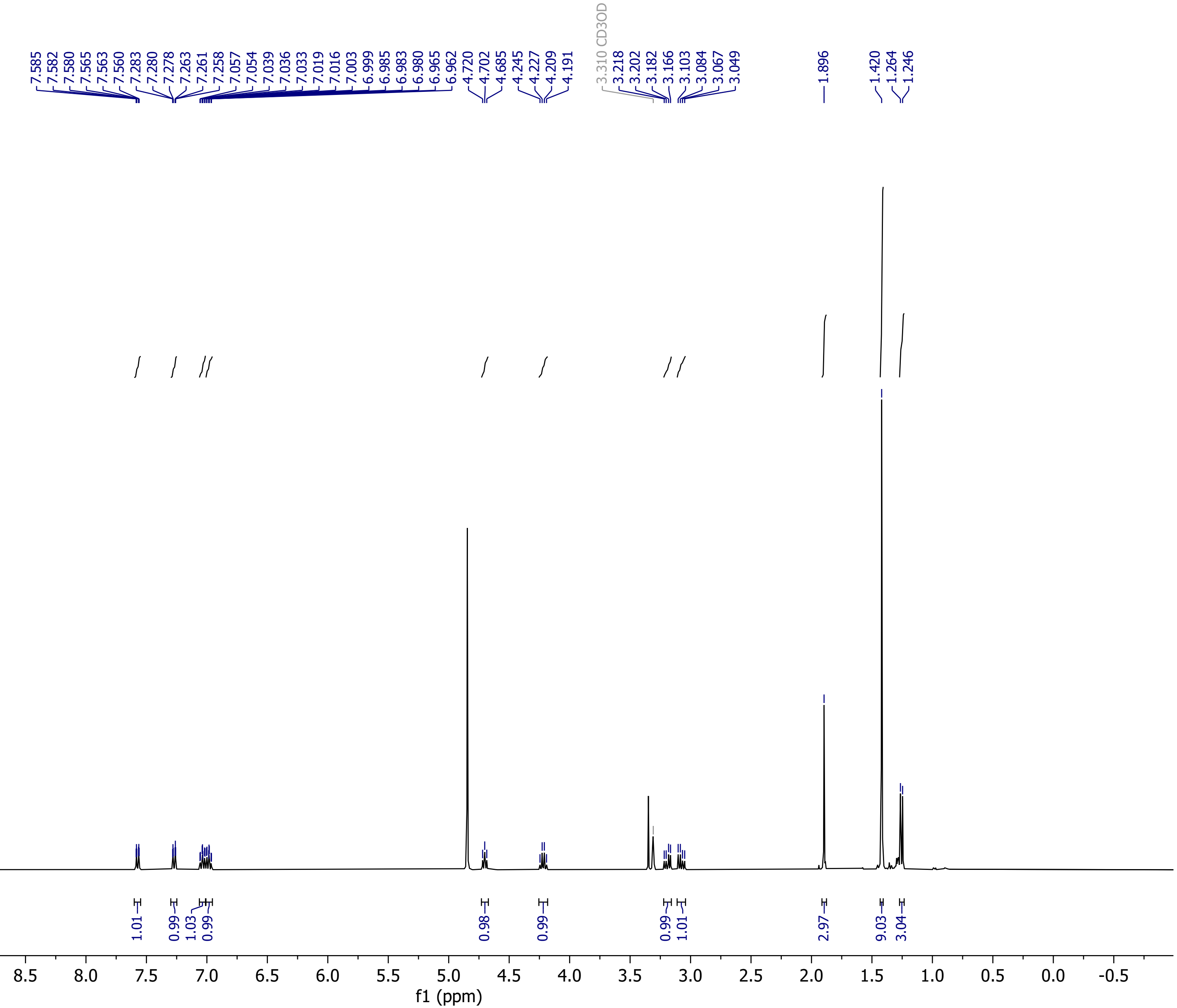

$^{13}C\{^1H\}$ NMR (101 MHz, CD_3OD)

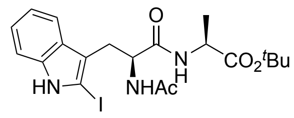

9

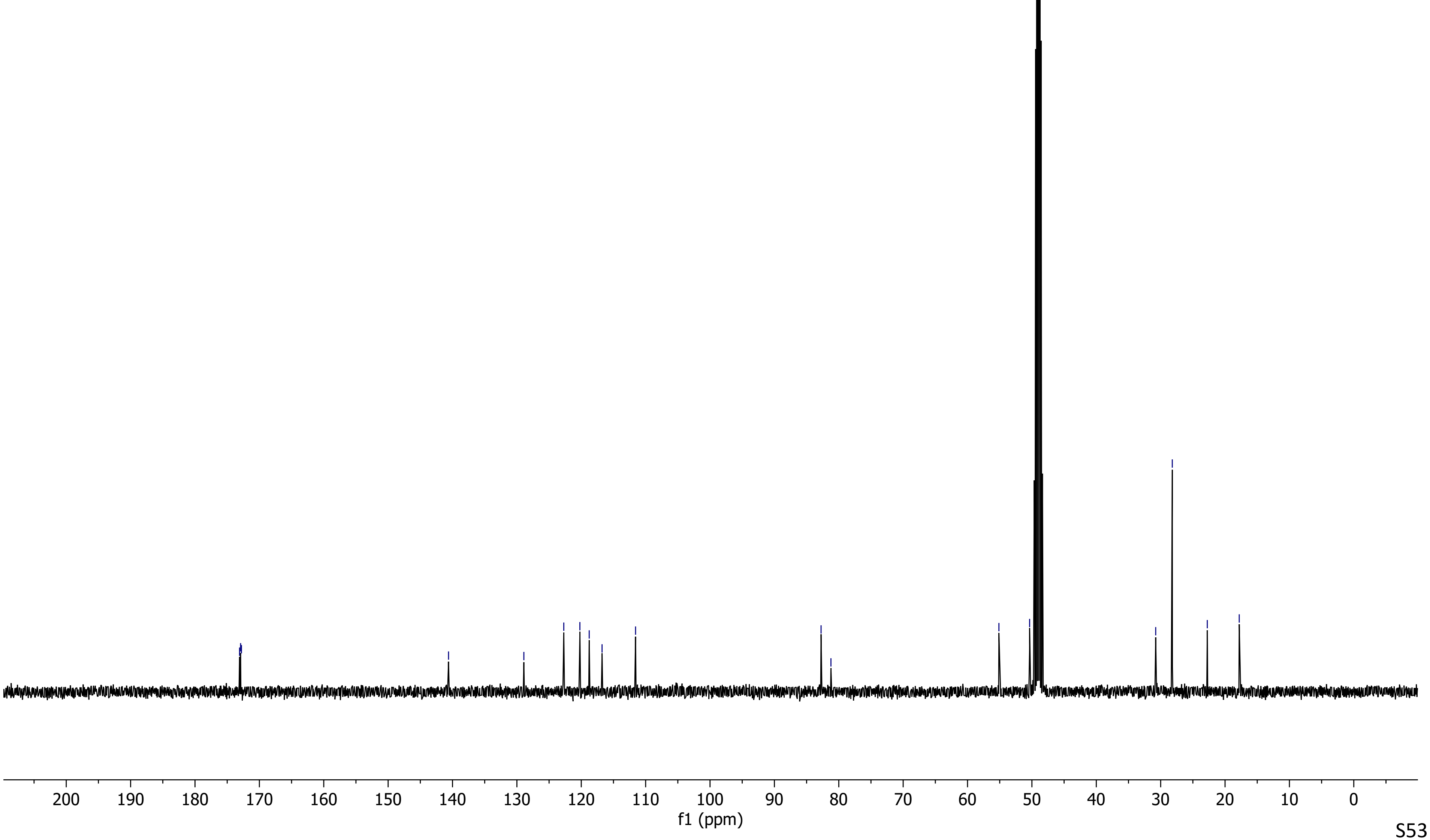

1H NMR (400 MHz, CDCl$_3$)

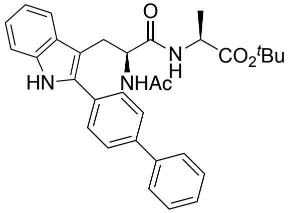

10

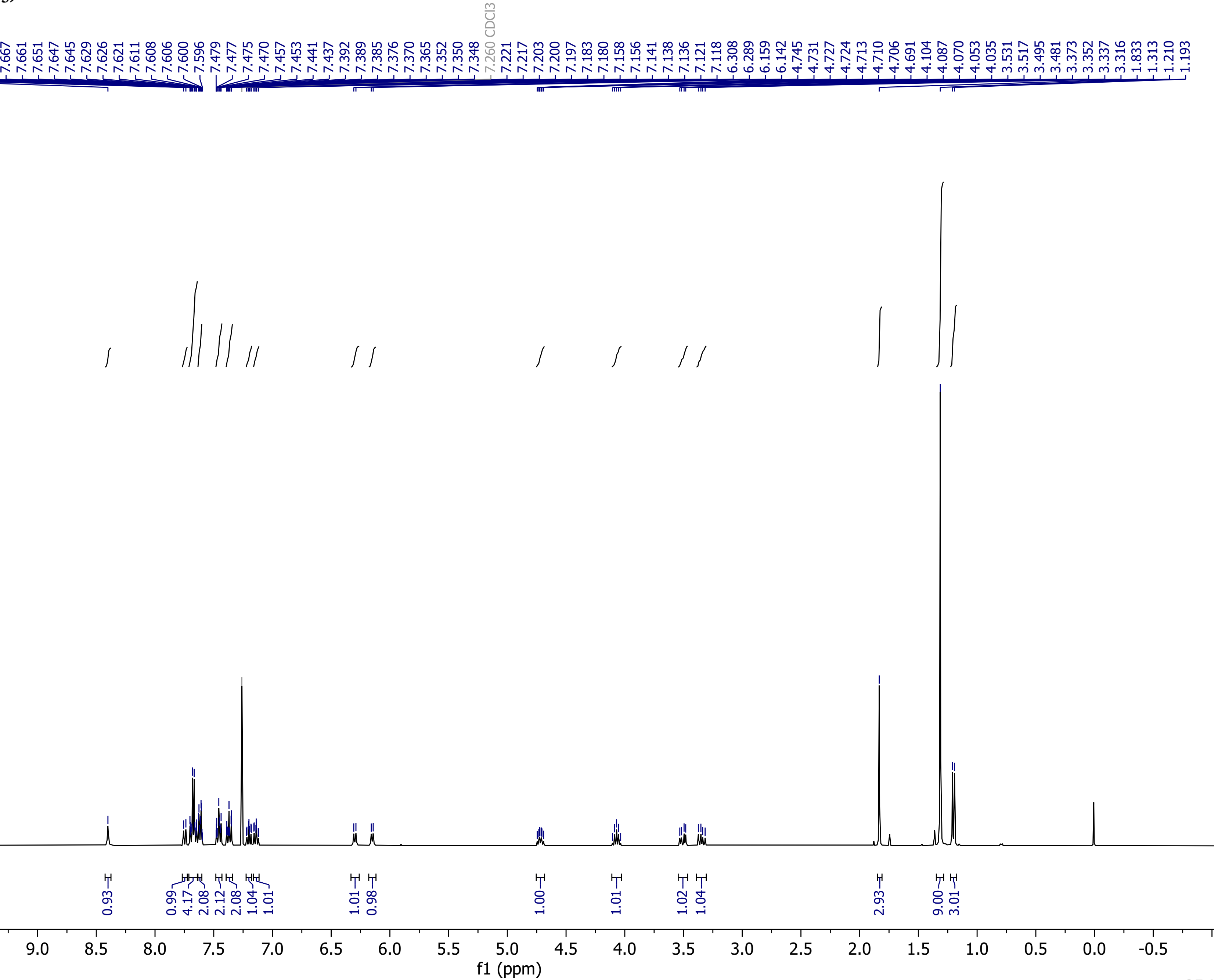

$^{13}C\{^{1}H\}$ NMR (101 MHz, $CDCl_3$)

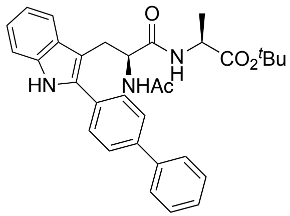

10

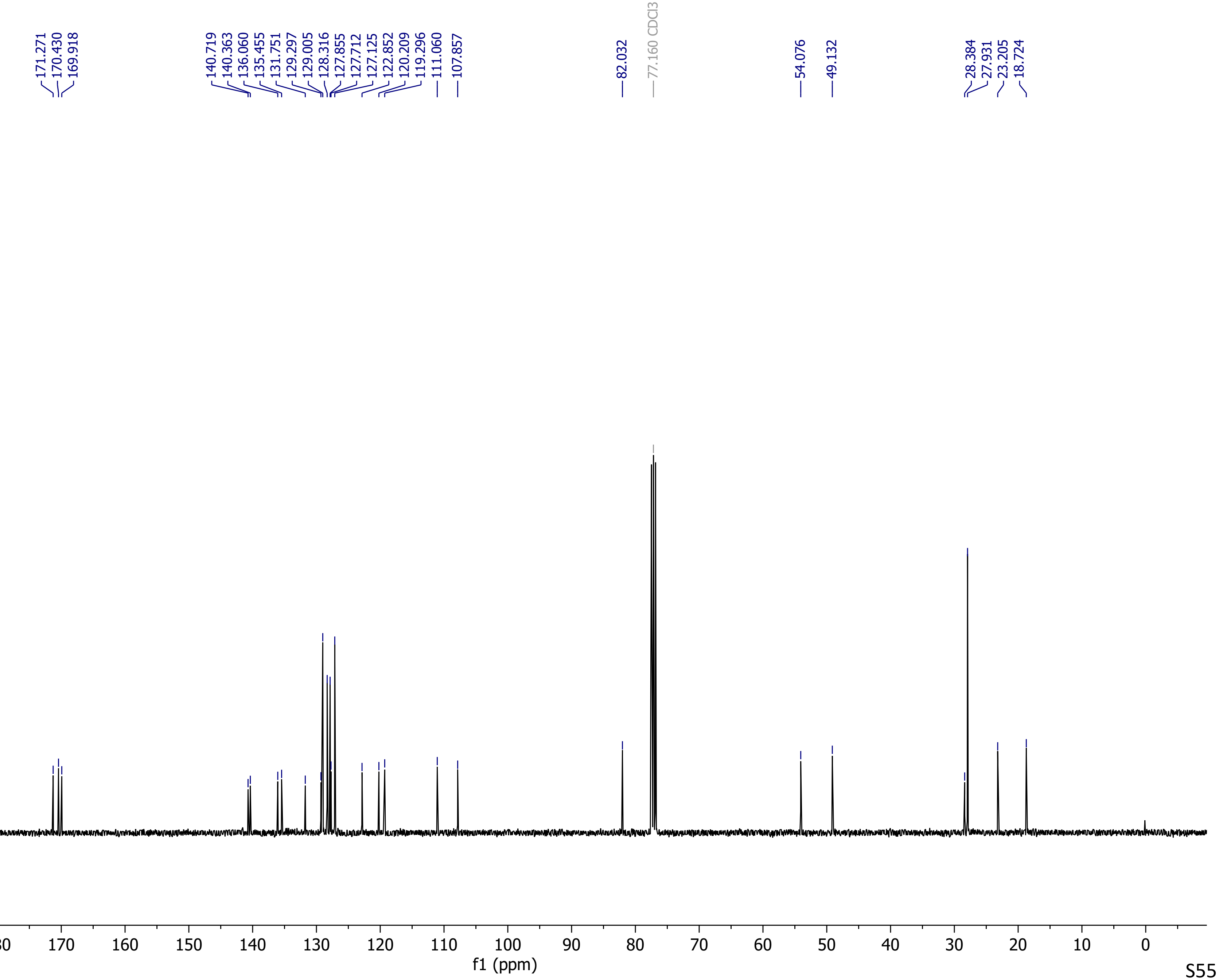

^{1}H NMR (400 MHz, CD_3OD)

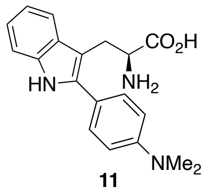

11

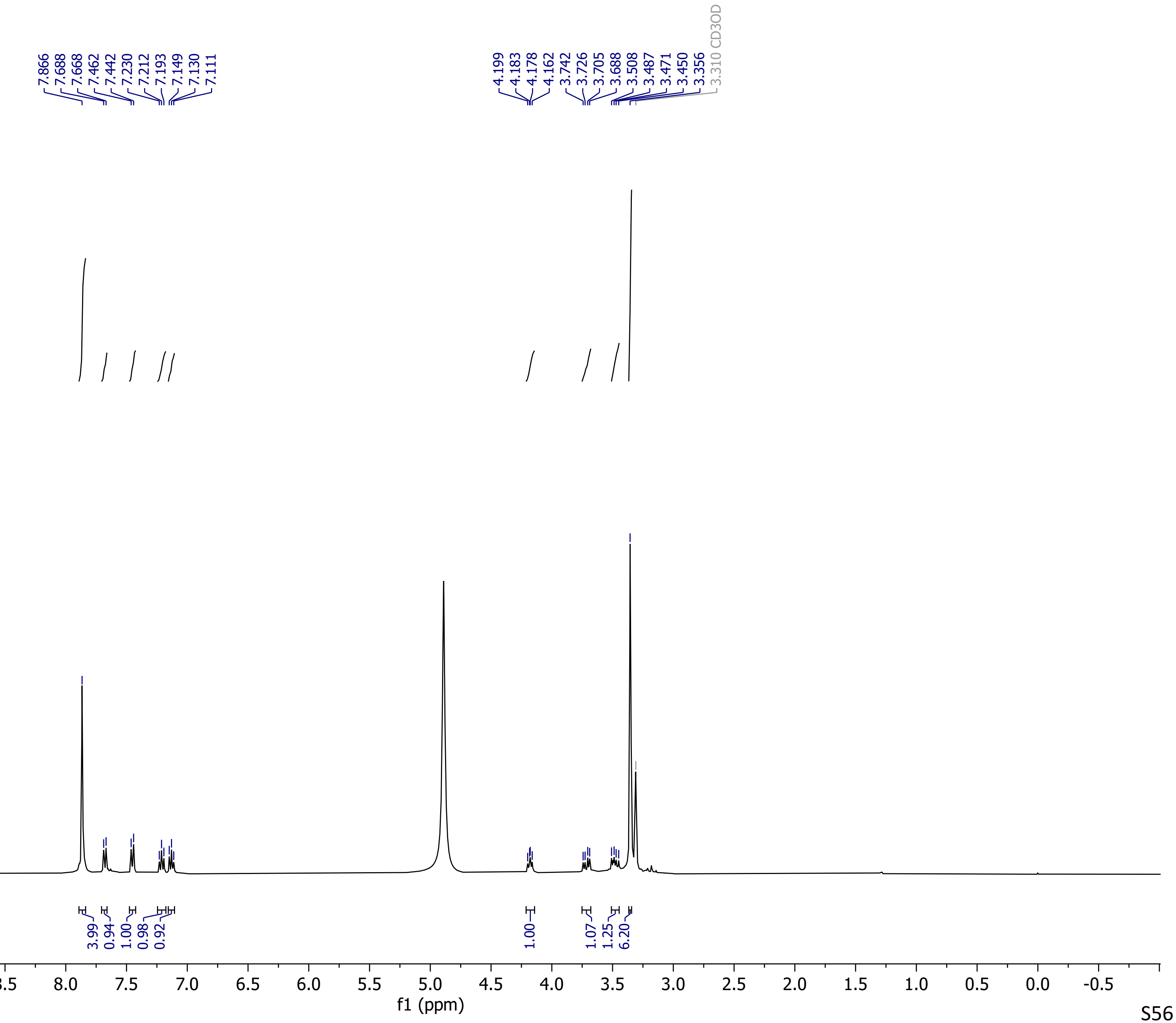

$^{13}C\{^1H\}$ NMR (101 MHz, CD_3OD)

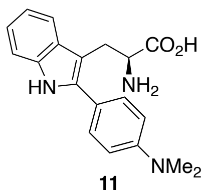

11

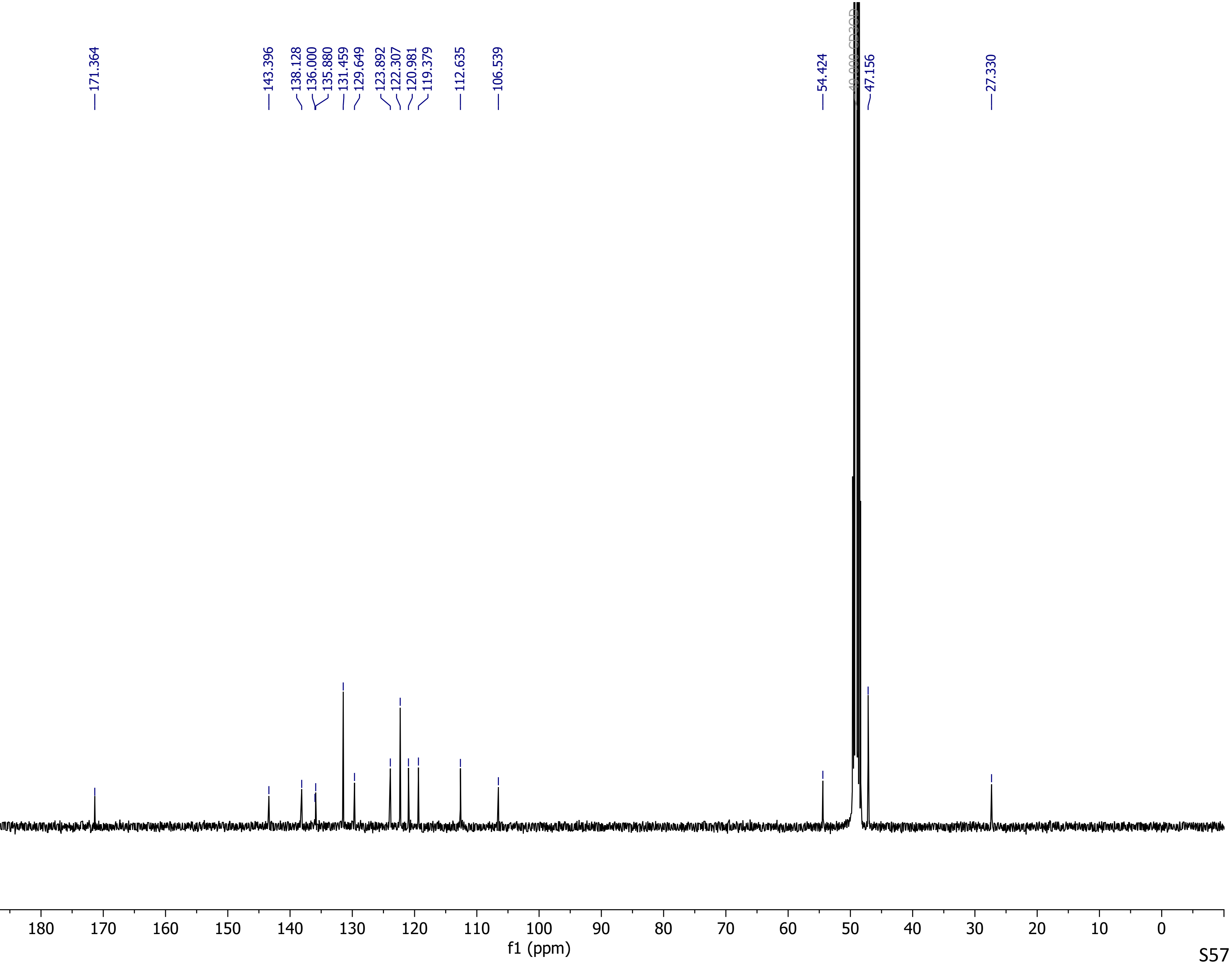

^1H NMR (400 MHz, $CDCl_3$)

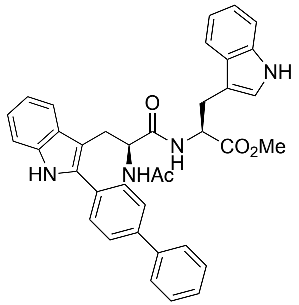

13

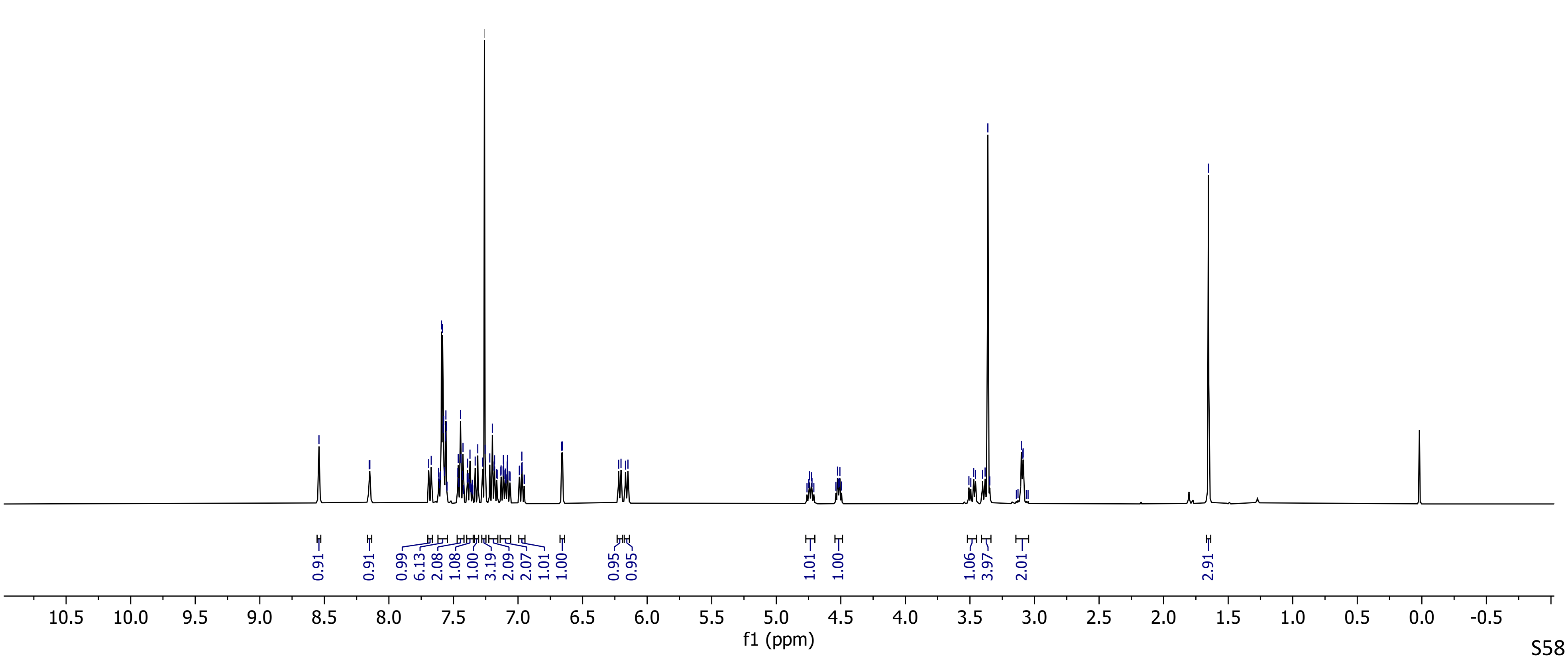

$^{13}C\{^1H\}$ NMR (101 MHz, $CDCl_3$)

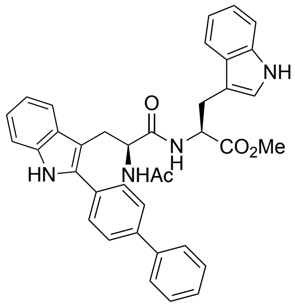

13

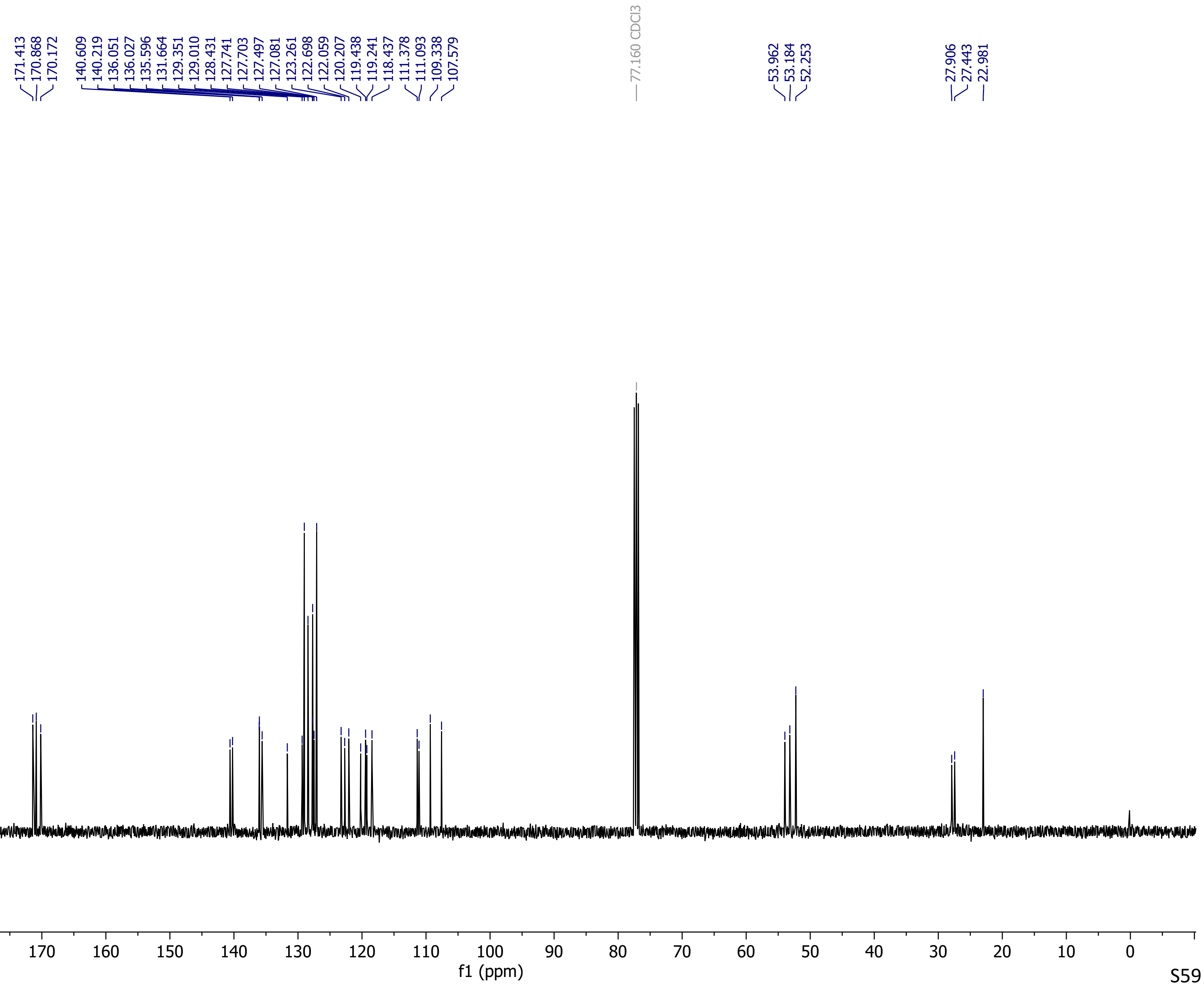

Supplement: Supplementary file 1 [file jo6c00381_si_001.pdf]
